# Exploration of alternative test methods to evaluate phototoxicity of ophthalmic agents by using Statens Seruminstitut Rabbit Cornea cell lines and 3D human reconstituted cornea models

**DOI:** 10.1371/journal.pone.0196735

**Published:** 2018-05-21

**Authors:** Soyoung Kim, Ki Hwan Choi, Jaesuk Yun

**Affiliations:** 1 National Institute of Food and Drug Safety Evaluation (NIFDS), Ministry of Food and Drug Safety (MFDS), OHTAC 187, Osongsaengmyong 2-ro, Cheongju-si, Chungbuk, Republic of Korea; 2 College of Pharmacy, Wonkwang University, Iksandaero, Iksan, Jeonbuk, Republic of Korea; University of Florida, UNITED STATES

## Abstract

Many chemicals have been reported to induce phototoxicity. The absorbance of light energy within the sunlight range is a common characteristic of phototoxicity. The 3T3 NRU phototoxicity test (PT) in 3T3 mouse skin fibroblasts has been used to identify the phototoxic potential induced by excited chemicals after exposure to ultra violet (UV). However, as phototoxicity may occur in ocular cells, it is necessary to develop a more suitable test for cornea-derived cells. In this study, we attempted to establish a new in vitro PT method in rabbit corneal cell lines (SIRC). We evaluated five ophthalmic agents, ciprofloxacin, levofloxacin, lomefloxacin, norfloxacin, and tetracycline, for their cytotoxic potential and in vitro phototoxicity. The results obtained using 3D human corneal models revealed that the UV-induced eye tissue toxicity by the test substances showed good correlation with those obtained using the in vitro phototoxicity test. However, the results from the 3D PT for ciprofloxacin, norfloxacin, and tetracycline in the 3D human cornea model were only partially comparable. Therefore, we suggest the SIRC cell line as a new phototoxicity test model; however, a sequential testing strategy, such as 3D PT, was also proposed to obtain relevant information for topical eye agents.

## Introduction

The phototoxic effects of cosmetics and pharmaceuticals is of interest to patients, toxicologists, and the relevant industries. The expanding market of medications and cosmetics, in combination with relatively high ultraviolet (UV) exposure, have potentiated this problem [1]. Predominantly, two categories of photoreaction occur in response to UVA: phototoxicity (photoirritation) and photoallergy [2, 3]. Exposure to several photosensitizers and light/UV radiation can elicit an acute toxic response [1] and chemical exposure after light/UV radiation induces an acute phototoxic reaction, called photoirritation [4]. Typical phototoxic effects include erythema, edema, vesiculation, and desquamation [5]. A wide range of pharmaceutical agents, such as chlorpromazine (CPZ), quinine, and NSAIDs such as fluoroquinolones and tetracyclines, are associated with phototoxicity [4]. The in vitro 3T3 NRU phototoxicity test (PT), which uses 3T3 mouse skin fibroblasts, measures cell viability and has been used to evaluate the phototoxicity induced by an excited chemical after exposure to ultraviolet (UV) irradiation [6, 7]. However, as adverse reactions in eyes should be considered, the development of more appropriate test methods for phototoxicity using cornea-derived cells is necessary. According to previous studies, toxicity results in primary corneal endothelial cells are comparable to those of rabbit corneal epithelial and SIRC cell lines [8, 9]. It has been reported that SIRC cells showed similar transcorneal absorption properties compared with excised cornea [10, 11]. Furthermore, in vitro cytotoxicity of SIRC cells correlated with eye irritation levels in vivo rabbit eye irritation assay [12]. In this study, we attempted to develop a new in vitro PT method using rabbit corneal cell lines, Statens Seruminstitut Rabbit Cornea (SIRC), through the evaluation of five ophthalmic agents, ciprofloxacin, levofloxacin, lomefloxacin, norfloxacin, and tetracycline. The results from the PT test in SIRC cells were comparable with those from 3D human cornea models.

## Materials and methods

### Reagents and preparation

Chlorpromazine (CAS. No. 69-09-0), ciprofloxacin (CAS. No. 85721-33-1), levofloxacin (CAS. No. 100986-85-4), lomefloxacin (CAS. No. 98079-52-8), norfloxacin (CAS. No. 70458-96-7), tetracycline (CAS. No. 64-75-5), L-histidine (CAS. No. 7006-35-1), and sodium lauryl sulfate (SLS, CAS. No. 151-21-3) were purchased from Sigma Chemical Co. (St. Louis, MO, USA). Ethanol and other standard chemicals were purchased from Sigma Chemical Co. (St. Louis, MO, USA), unless otherwise mentioned. Chlorpromazine was dissolved in ethanol, tetracycline was dissolved in DMSO (Sigma), L-histidine and sodium lauryl sulfate were dissolved in phosphate-buffered saline (PBS, Gibco BRL, NY, USA), and ciprofloxacin and norfloxacin were dissolved in PBS with 0.1 N NaOH (pH 7.5~8.0).

### Cell culture and UV irradiation

SIRC (ATCC, VA, USA) were cultured in Eagle's Minimum Essential Medium (EMEM) supplemented with 10% FBS and 1% penicillin–streptomycin (Gibco BRL), and maintained under a completely humidified atmosphere of 95% air/5% CO_2_ at 37°C. UV irradiation was performed by using a UV-Bio-Spectra (Vilber Lourmat, Marne-La-Vallee, France).

### In vitro phototoxicity test

The in vitro phototoxicity test was conducted in accordance with the modified OECD 3T3 NRU phototoxicity test guideline (TG) No. 432 (13). Briefly, 96-well tissue culture plates were seeded with 1×10^4^ SIRC cells and incubated at 37°C in a humidified 5% CO_2_ incubator for 24 h. The cells were then exposed to various dilutions of the test materials in Earle’s Balanced Salt Solution (EBSS) for 60 min. The test substances were applied up to a maximum concentration of 1000 μg/mL with three-fold serial dilutions (a total of eight concentrations), both with and without UV irradiation. Chlorpromazine (CPZ) was used as a positive control [13]. One set of plates was irradiated with a non-toxic dose (5 J/cm^2^) of UVA (+Irr, as measured in the UVA range), and the other set was maintained in the dark (-Irr). After irradiation, all solutions were removed from the plates, the cells were washed with EBSS, reincubated in culture medium, and incubated overnight. Cell viability was measured by Neutral Red uptake methods. Phototox software (ZEBET, Germany) was used to determine the probability (p-value), Photo Irritation Factor (PIF) and Mean Photo Effect (MPE) of the test chemical being phototoxic according to previous study [14]. Probability was obtained by using PIF/MPE values and the computer-generated concentration-response data [15]. We performed experiments (n = 6) and determined the phototoxic risk based on the criteria (Table 1.) [15]. Phototoxicity was predicted using the cut-off value of PIF (2) or MPE (0.1).

**Table 1:** Phototoxicity criteria in NRU test.

PIF	MPE	PPH
PIF <2	<0.1	No phototoxicity
2< PIF <5	0.1< MPE <0.15	Probable phototoxicity
5< PIF	0.15 < MPE	Phototoxicity

The potential phototoxicity hazard of a substance was determined based on above criteria. Phototoxicity is predicted using the cut-off value of photo irritation factor (PIF, 2) or mean photo effect (MPE, 0.1). PPH; predicted phototoxic hazard.

### Immunostaining

3D-cultured primary human corneal epithelial tissues (HCE model) were purchased from MCTT (Seoul, Korea). The HCE model cells were fixed with 10% formaldehyde, embedded in paraffin, and sliced into 4-μm sections by using a RM2255 Microtome (Leica, Wetzlar, Germany). The paraffin sections were deparaffinized in xylene and rehydrated in a series of decreasing concentrations of ethanol. Hematoxylin and eosin staining was performed for histological analysis of tissues was measured by using DP2-BSW software (Olympus).

### 3D phototoxicity test

The 3D PT for chlorpromazine, ciprofloxacin, norfloxacin, tetracycline, L-histidine, and sodium lauryl sulfate was conducted according to previous study [16]. The 3D human cornea model, HCE model, was supplied by MCTT, South Korea. MCTT 3D epithelium model present corneal phenotypes such as columnar basal cell layer, wing cells and superficial squamous cells, and a cornea-specific keratin pair, cytokeratin 3/12 protein [16]. Prior to dosing, the tissues were pre-incubated in fresh medium for 1 h (37°C, 5% CO_2_). The test materials were diluted in distilled water and applied for 24 h in a volume of 50 μL per tissue. One set of tissues was irradiated with a non-toxic dose (6 J/cm^2^) of UVA (+Irr, as measured in the UVA range), and the other set was maintained in the dark (-Irr). After overnight incubation at 37°C with < 5% CO_2_, cytotoxicity was detected by Neutral Red uptake methods. Phototox software (ZEBET, Germany) was used to determine the probability (p-value) of the test chemical being phototoxic according to previous study [14]. We performed experiments (n = 6) and determined the phototoxic risk as above-mentioned methods.

## Results

**Fig 1:**
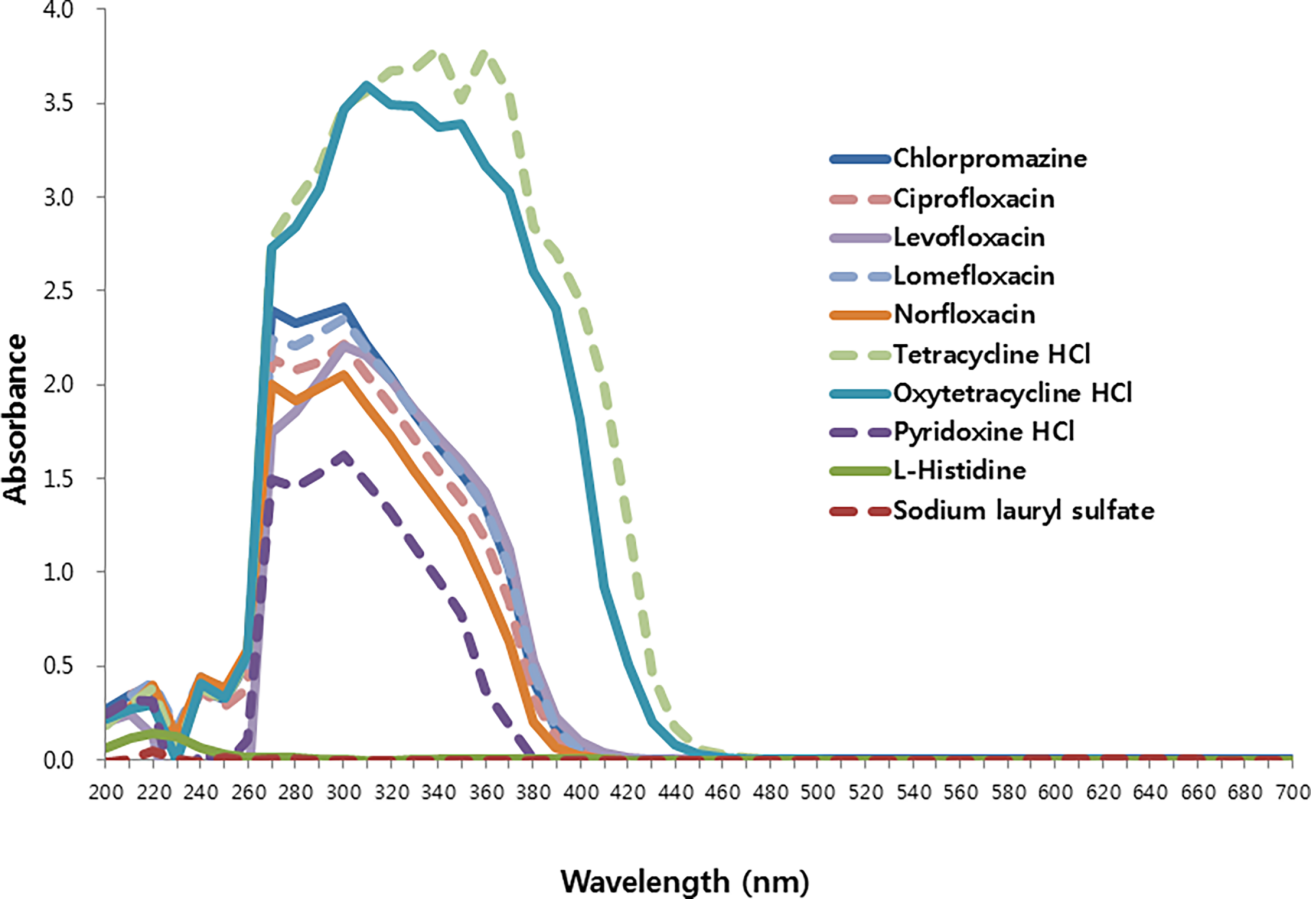
Spectral properties of chlorpromazine (positive control), ciprofloxacin, levofloxacin, lomefloxacin, norfloxacin, and tetracycline (test substances), and L-histidine and sodium lauryl sulfate (negative controls).

The UV absorption ranges of chlorpromazine, L-histidine, and sodium lauryl sulfate are shown in Fig 1 and S1 File. Ciprofloxacin, levofloxacin, lomefloxacin, norfloxacin, and tetracycline have a broad absorption spectrum in the UVA range, whereas the negative controls, L-histidine and sodium lauryl sulfate, exhibited low or no absorption at the same UV wavelengths.

**Fig 2:**
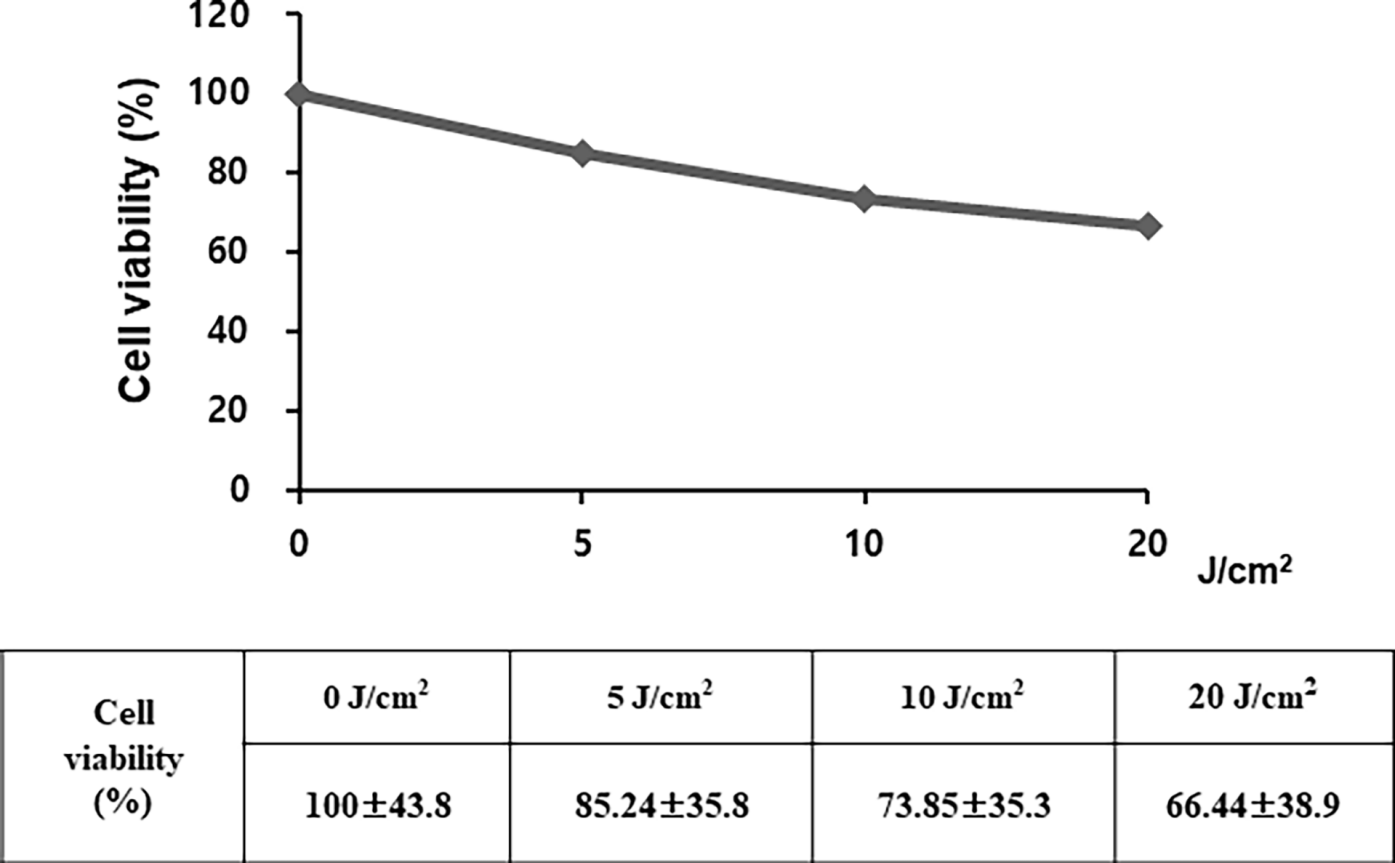
Irradiation sensitivity of SIRC cells. Cell viability showed a dose-dependent response to irradiation, with 85.2%±4.9% after irradiation at 5 J/cm^2^, 73.8%±3.4% after irradiation at 10 J/cm^2^, and 66.4%±2.9% after irradiation at 20 J/cm^2^. The data are expressed as the mean ± S.E. (n = 5).

**Fig 3:**
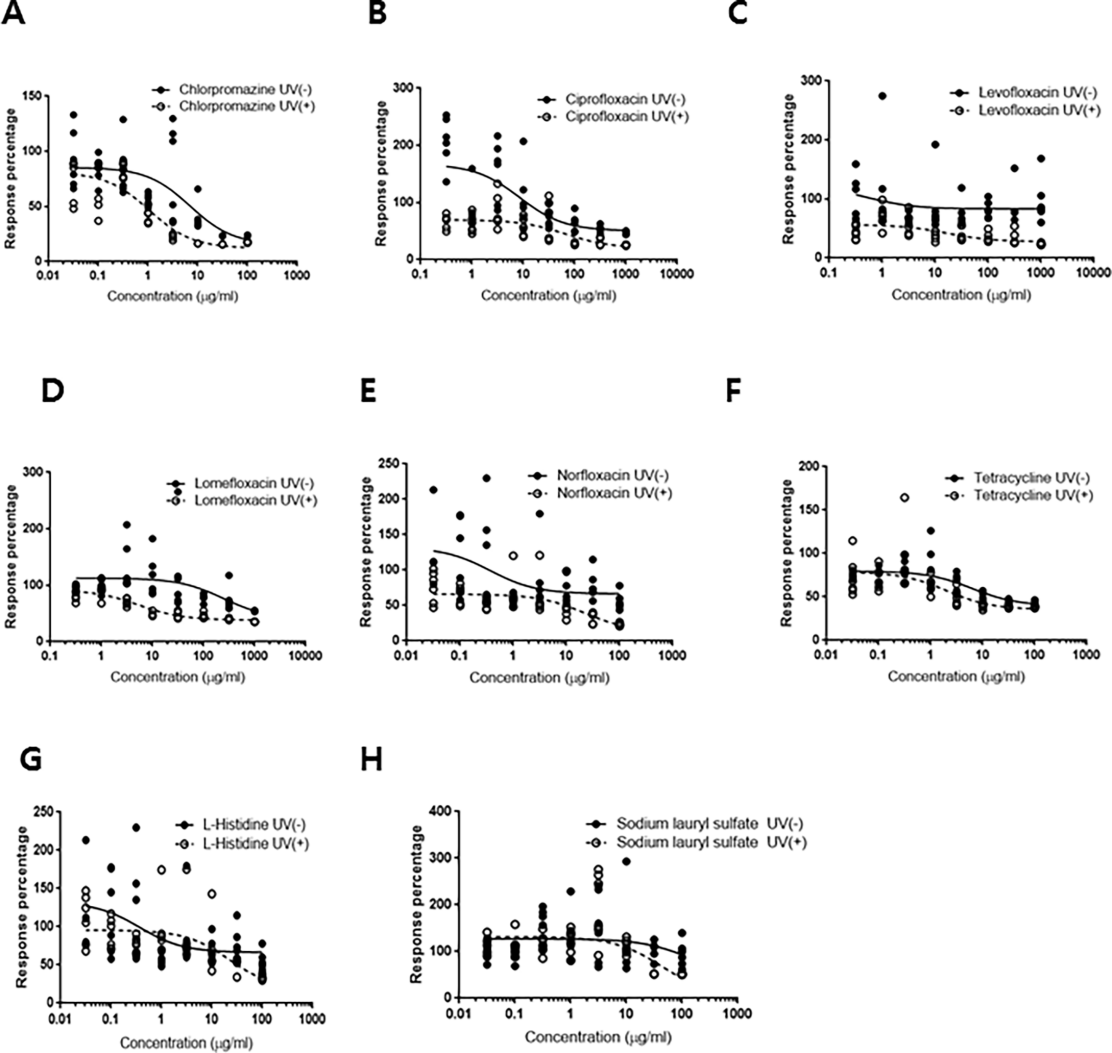
Phototoxicity evaluation of the ophthalmic agents in SIRC cells: A) chlorpromazine (positive control), B) ciprofloxacin, C) levofloxacin, D) lomefloxacin, E) norfloxacin, F) tetracycline, G) L-histidine (negative control), and H) sodium lauryl sulfate (negative control). The closed circle and open circle represent data from the nonirradiated groups and UV-irradiated groups, respectively. Phototoxic chemicals induced dose-response curve shift with UV-irradiation. Fitting of the curve to the data was performed by a non-linear regression method (n = 6).

The sensitivity of SIRC cells to UV irradiation was evaluated (Fig 2 and S2 File). The cells exhibited a dose-dependent response to irradiation, with viabilities of 85.2%±4.9% at 5 J/cm^2^, 73.8%±3.4% at 10 J/cm^2^, and 66.4%±2.9% at 20 J/cm^2^. Therefore, a UVA dosage of 5 J/cm^2^ was irradiated in compliance with OECD TG 432 [17]. The results from the SIRC phototoxicity test with the test materials are presented in Fig 3, S3 File, and summarized in Table 2. The dose–response curves were constructed for each experiment, and the effective concentration of test materials that resulted in a 50% reduction of viability (IC_50_ value) was calculated as the ratio of toxicity for each substance, with and without UV light. The experiments were considered acceptable based on the recommendation of OECD TG 432 with the following ranges: the IC_50_ values for CPZ were in the ranges of 0.1–2.0 μg/mL with irradiation and 7.0–90.0 μg/mL without irradiation, and the photoirritation factor (PIF) for CPZ should be higher than 14.4 [15]. The potential phototoxicity hazard of a substance was determined based on the following criteria: PIF < 2 or Mean Photo Effect (MPE) < 0.1 predicts “no phototoxicity”; 2 < PIF < 5 or 0.1 < MPE < 0.15 predicts “probable phototoxicity”; and PIF > 5 or MPE > 0.15 predicts “phototoxicity” (Table 1.) [17]. The PIF value of 9.651 was a clear indication of the phototoxic nature of CPZ, with acceptable results in the phototoxicity assessment. Ciprofloxacin, levofloxacin, lomefloxacin, and norfloxacin were found to be phototoxic, with MPE values of 0.326, 0.293, 0.332, and 0.177, respectively. The test results for tetracycline predicted the substance to have probable phototoxicity, with a borderline MPE value of 0.15. As expected, the two negative controls (L-histidine and sodium lauryl sulfate) were classified as non-phototoxic substances, with MPE values of 0.009 and 0.008, respectively.

**Table 2:** Comparison of phototoxicity test results from in vitro SIRC, 3D human model and 3T3 NRU phototoxicity test.

Test substances	SIRC			3D human model			3T3 NRU			
	MPE	PIF	PPH	MPE	PIF	PPH	MPE	PIF	PPH	Ref.
Chlorpromazine	0.425	9.651	PT	0.158	8.393	PT	0.33–0.63	>14.4	PT	[15, 32, 33]
Ciprofloxacin	0.326	1	PT	0.068	1	NPT	0.49	15.9	PT	[23]
Levofloxacin	0.293	1	PT	NT	NT	NT	0.76	32.2	PT	[23]
Lomefloxacin	0.332	1	PT	NT	NT	NT	0.57	46.6	PT	[23]
Norfloxacin	0.203	1	PT	0.152	1	PT	0.34–0.90	>71.6	PT	[15, 32]
Tetracycline	0.150	3.389	PT	0.020	1	NPT	0.67	86.4	PT	[23]
L-histidine	0.009	1	NPT	-0.030	1	NPT	0.05–0.10	No PIF	NPT	[15, 32]
Sodium lauryl sulfate	0.008	1	NPT	0.043	0.946	NPT	0.00–0.05	1.0–1.9	NPT	[15, 32]

The experiments were performed in triplicate and the results presented are representative of six independent experiment: MPE; mean photo effect, PIF; photo-irritation factor, PPH; predicted phototoxic hazard, PT; phototoxic, NPT; non-phototoxic, NT; not tested.

**Fig 4:**
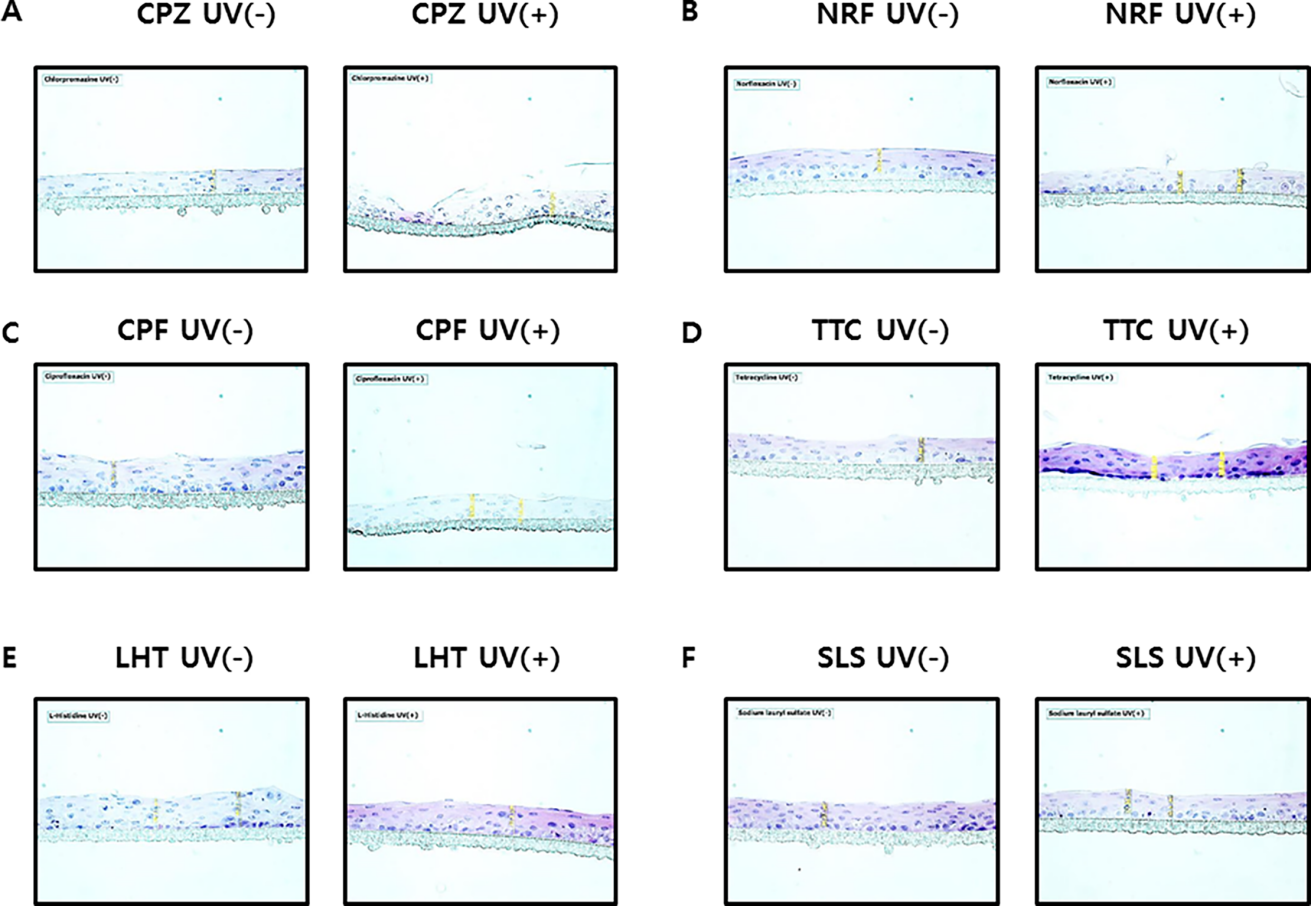
Hematoxylin and eosin staining of 3D human cornea models (HCE) of the ophthalmic agent, with and without UV irradiation of 6 J/cm^2^: A) chlorpromazine (positive control), B) ciprofloxacin, C) norfloxacin, D) tetracycline, E) L-histidine (negative control), and F) sodium lauryl sulfate (negative control).

Next, we compared the phototoxicity results from the SIRC cell lines and the HCE model. The immunohistochemical staining patterns of the HCE model cells treated with chlorpromazine, ciprofloxacin, norfloxacin, and tetracycline and exposed to UV are presented in Fig 4 and S1 Fig. In the groups treated with chlorpromazine and norfloxacin, the 3D tissues were affected by UV irradiation, whereas the ciprofloxacin and tetracycline-treated groups showed only slight tissue damage. L-Histidine and sodium lauryl sulfate, which showed almost no UV absorption, did not exhibit phototoxic effects after UV irradiation. The test results for phototoxicity obtained from Phototox software (ZEBET, Germany) were consistent with the results of the immunohistochemical analyses (Table 2): CPZ and norfloxacin were phototoxic, whereas ciprofloxacin, tetracycline, L-histidine, and sodium lauryl sulfate were not phototoxic.

## Discussion

The 3T3 NRU Phototoxicity Test is a validated testing method for the identification of phototoxicity hazard [4, 6, 7]. However, there are some limitations: (1) the assay lacks in vivo–in vitro correlation, as bioavailability and biokinetics cannot be modeled; (2) the assay is only reasonably predictive of photoirritation and does not predict in vivo photoirritation and photoallergy [18]; (3) the assay is not specifically validated for pharmaceuticals [19] and has a much lower specificity for drug substances [20, 21]. In this study, the test substances were topically applied ophthalmic agents with known phototoxicity [20, 22], which were evaluated in SIRC rabbit corneal cell lines. The phototoxic potential was predicted for ciprofloxacin, levofloxacin, lomefloxacin, norfloxacin, and tetracycline by using the MPE and/or PIF values. The PIF is based on a comparison of two equally effective cytotoxic chemical concentrations (IC_50_ values) obtained from concurrently performed experiments in the presence (+UV) and absence (-UV) of UVA irradiation [7]. As the PIF could be only calculated in cases in which both IC_50_ values exist, it cannot be calculated if a chemical is cytotoxic in the presence of UV and not in the absence of UV. In this study, PIF values were not calculated for ciprofloxacin, levofloxacin, lomefloxacin, and norfloxacin (Table 2., assigned as 1 [7]), because no IC_50_ values could be determined in the absence of UV, which indicated that these compounds had low cytotoxic effects in the absence of UV at concentrations up to 1,000 μg/mL. Under these conditions, MPE values were used to evaluate the phototoxicity. MPE values are based on the comparison of the +UV and–UV concentration–response curves from the concentrations obtained from experiments conducted in the dark and the presence of light [7, 15, 17]. A comparison of the results from the 3T3 NRU PT [23] and OECD TG 432 assays revealed that the test results in SIRC PT showed comparable predictivity with 100% of the eight test substances, but yielded overall lower scores for the MPE and/or PIF values (Table 2.).

Nevertheless, the phototoxicity test results in cell lines without additional information on target organ penetration has only limited value for substances topically applied to the skin or eyes. As UVA penetrates deep into the eye, the 3D human cornea system would be a better model for the measurement of phototoxicity in human eyes, owing to the increased bioavailability of the phototoxic substance [24, 25]. Norfloxacin induced phototoxicity in the 3D human corneal models; however, ciprofloxacin did not cause any phototoxic effects. The borderline positive result in the SIRC PT for tetracycline (MPE = 0.15) was not confirmed in the 3D human cornea models. These results provide an explanation for the absence of the reported clinical phototoxicity of ciprofloxacin and tetracycline, despite their reported in vitro and/or in vivo phototoxicity [4, 26–29].

The results of this study are well correlated with findings of previous studies [1, 30]. Not all chemicals with photoactivation and phototoxic properties in the 3T3 NRU PT are hazardous to human eyes. Therefore, phototoxicity assessment in SIRC corneal cell lines might present an advantageous method for screening a large number of substances, especially those applied topically to the eye, for their potential phototoxicity. The 3D human cornea models can simulate real exposure conditions, including the parameters of eye penetration in humans. By using stepwise sequential screening tests, more relevant information on the potential phototoxicity of substances for topical eye use has to be obtained, as suggested by the OECD Guideline [31] for testing acute eye irritation.

## Supporting information

S1 File Cell viability. (XLSX)

S2 File UV spectrum. (XLS)

S3 File Phototox data. (ZIP)

S1 Fig HCM(H&E) staining. (ZIP)
