# Supplementary figures and images for: Exploration of alternative test methods to evaluate phototoxicity of ophthalmic agents by using Statens Seruminstitut Rabbit Cornea cell lines and 3D human reconstituted cornea models
Source: PLoS One. 2018 May 21;13(5):e0196735. doi: 10.1371/journal.pone.0196735 (PMC5962060; doi:10.1371/journal.pone.0196735)

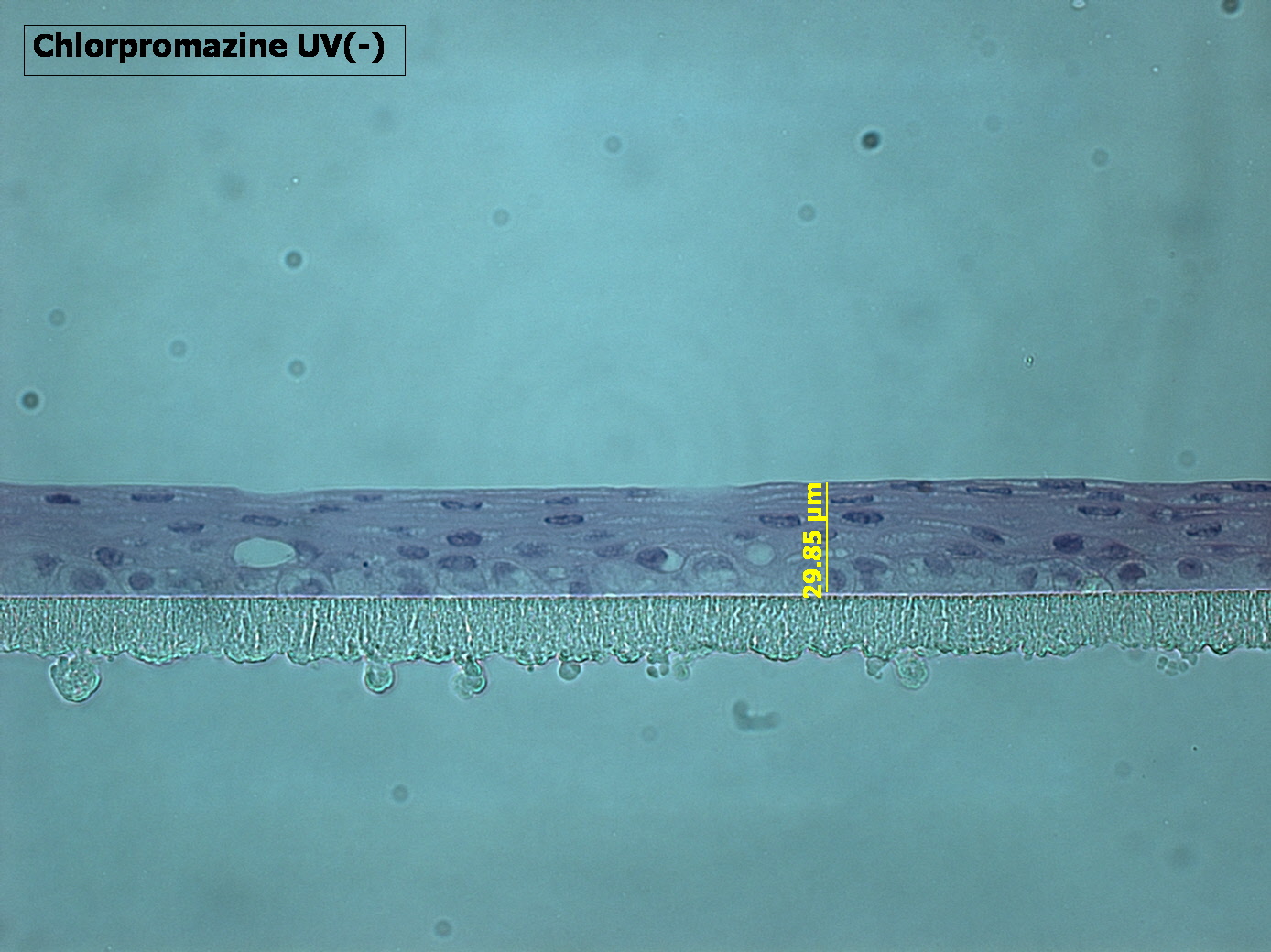

Supplement: S1 Fig — (ZIP) [file pone.0196735.s004.zip › HCM(H&E) staning raw data/HCM/Cornea_C-/SNAP-103430-0050_1.jpg]

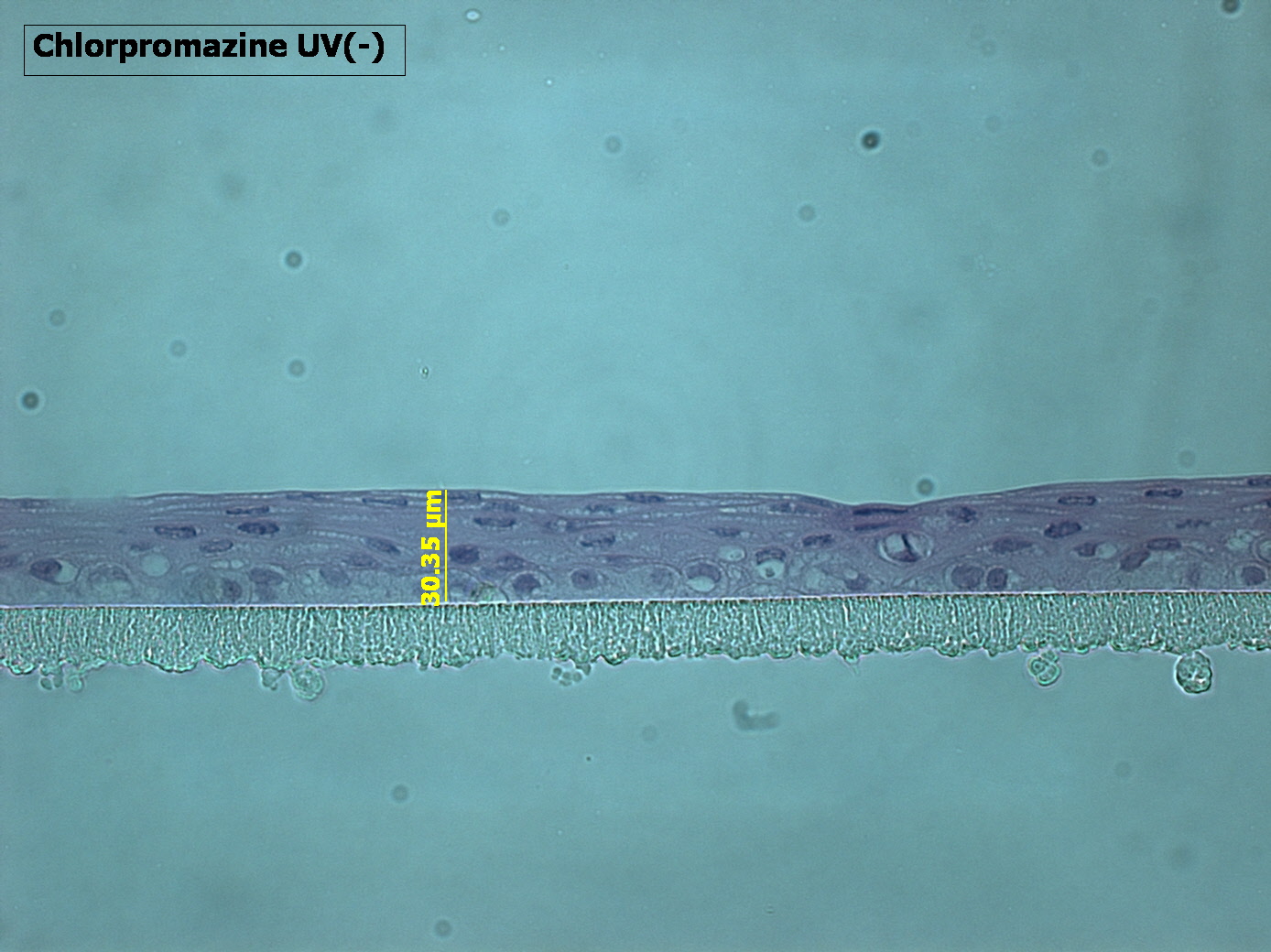

Supplement: S1 Fig — (ZIP) [file pone.0196735.s004.zip › HCM(H&E) staning raw data/HCM/Cornea_C-/SNAP-103435-0051_1.jpg]

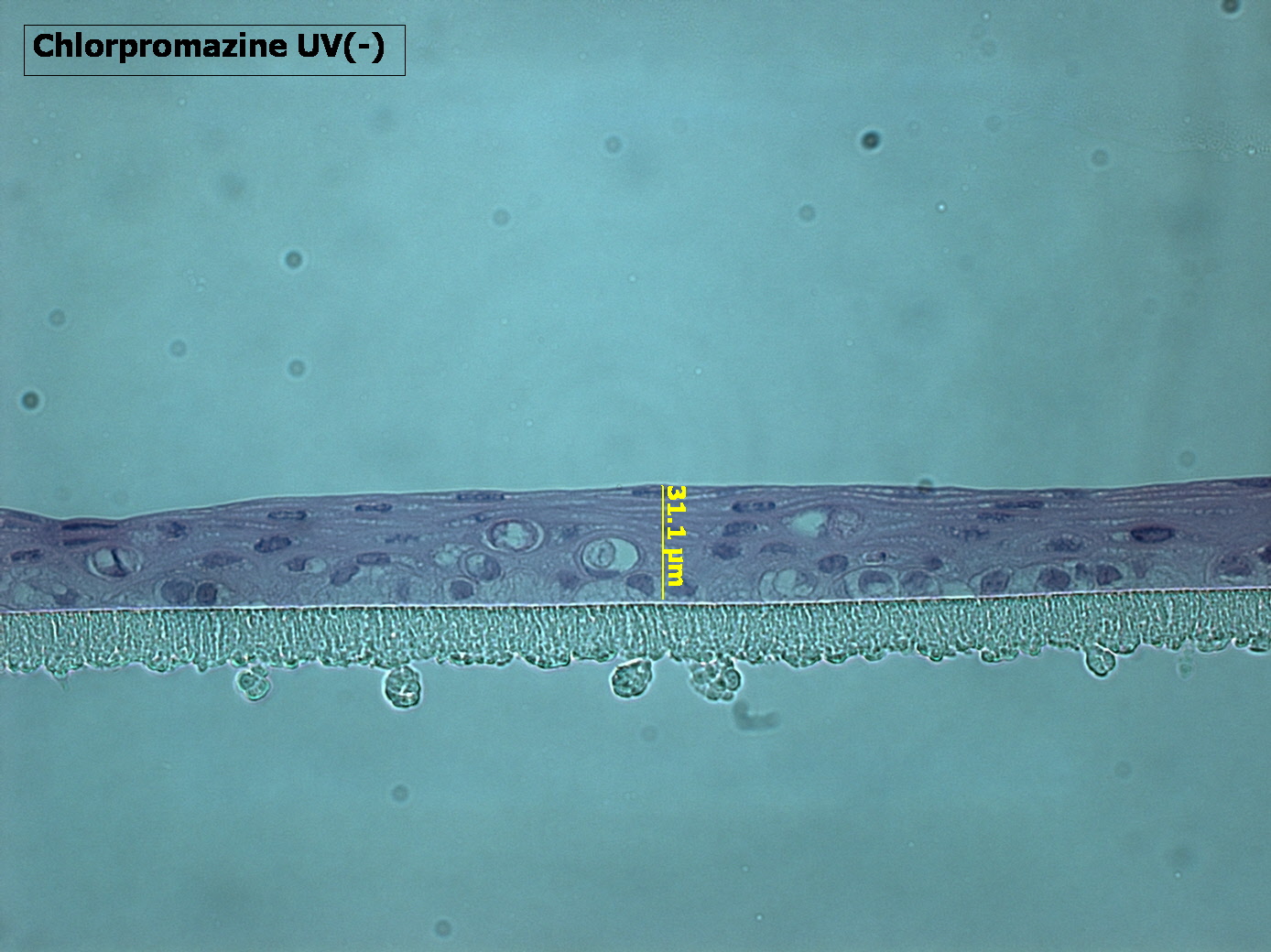

Supplement: S1 Fig — (ZIP) [file pone.0196735.s004.zip › HCM(H&E) staning raw data/HCM/Cornea_C-/SNAP-103442-0053_1.jpg]

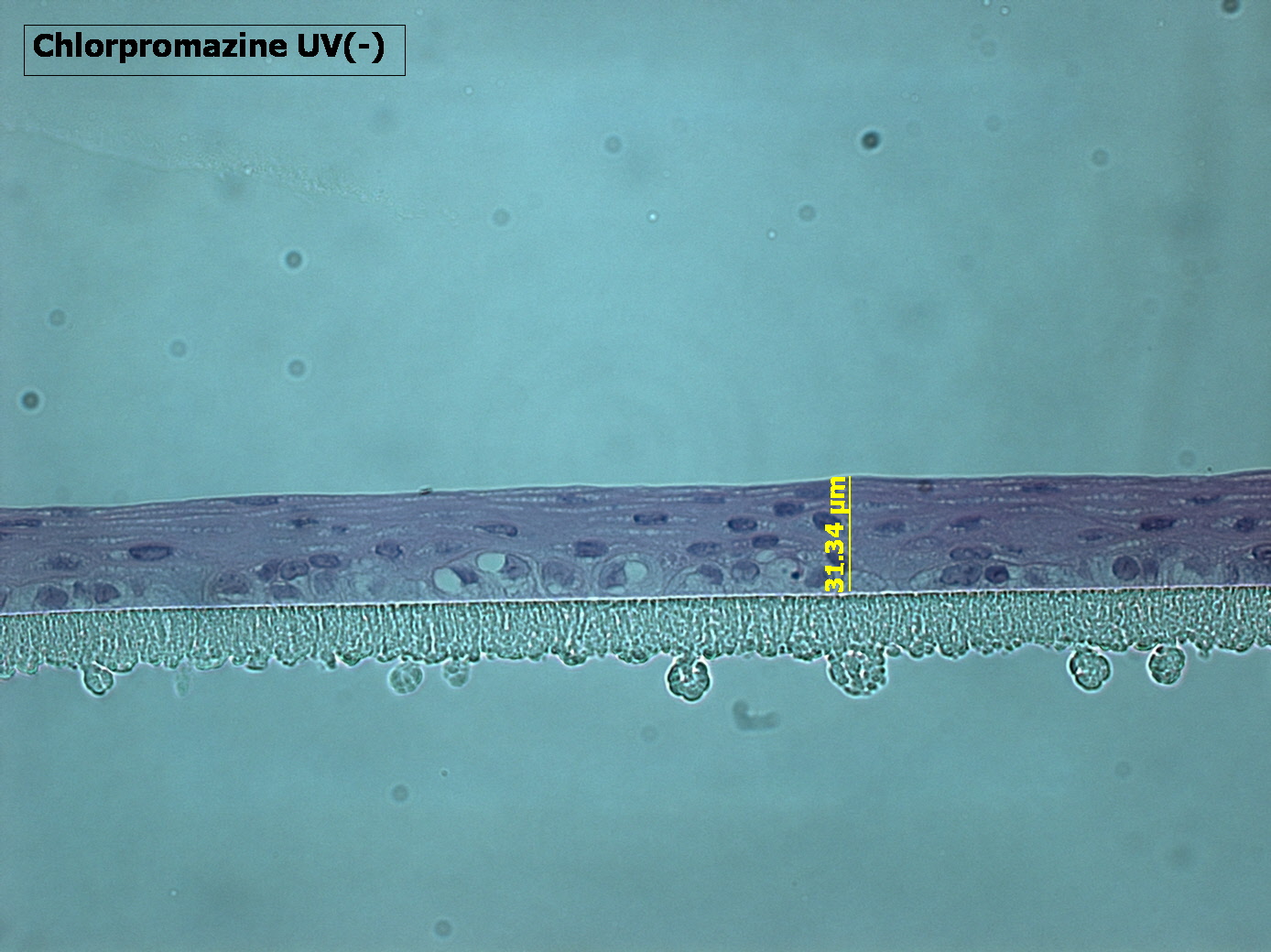

Supplement: S1 Fig — (ZIP) [file pone.0196735.s004.zip › HCM(H&E) staning raw data/HCM/Cornea_C-/SNAP-103459-0055_1.jpg]

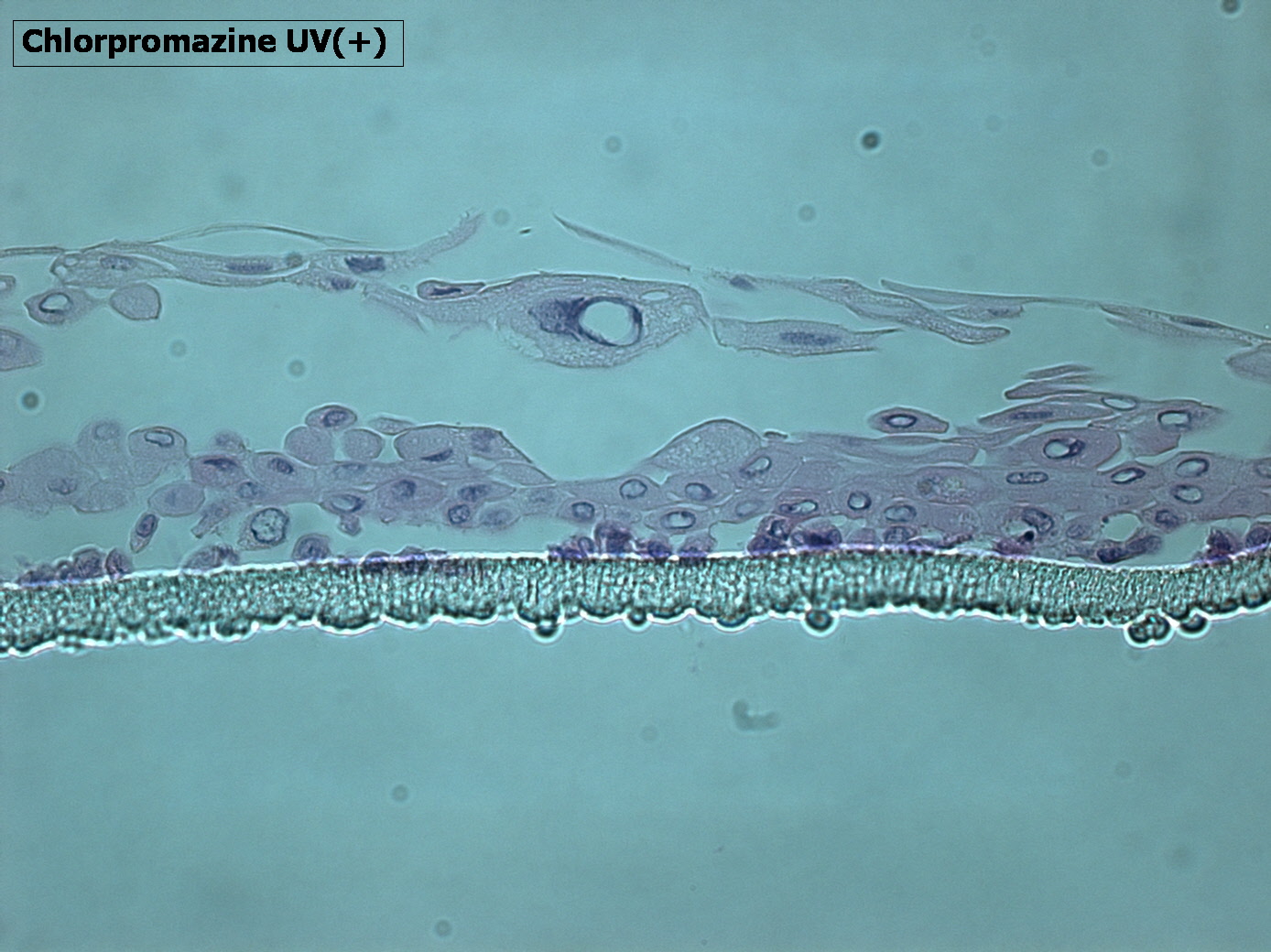

Supplement: S1 Fig — (ZIP) [file pone.0196735.s004.zip › HCM(H&E) staning raw data/HCM/Cornea_C+/SNAP-104225-0058_1.jpg]

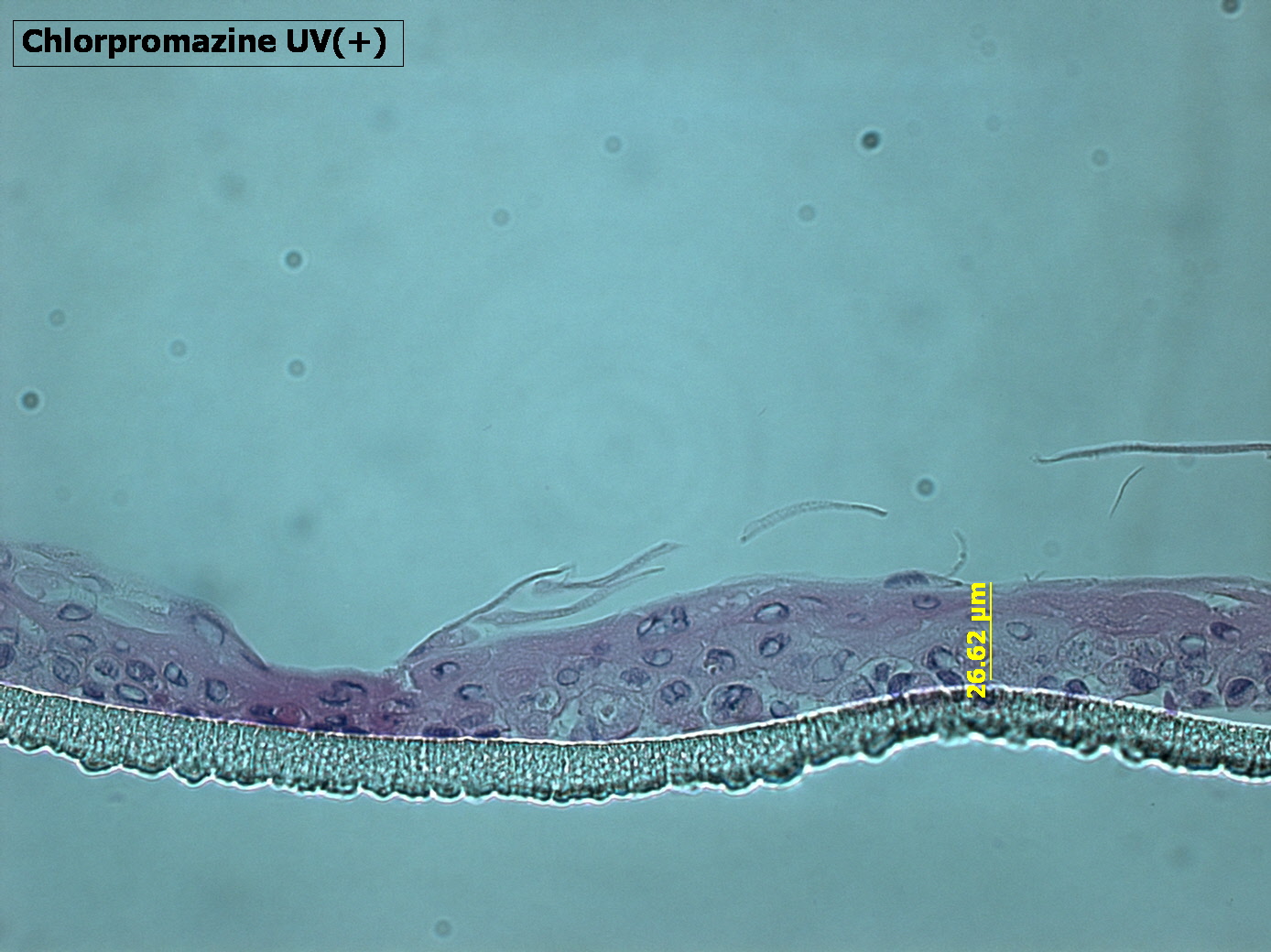

Supplement: S1 Fig — (ZIP) [file pone.0196735.s004.zip › HCM(H&E) staning raw data/HCM/Cornea_C+/SNAP-104523-0062_1.jpg]

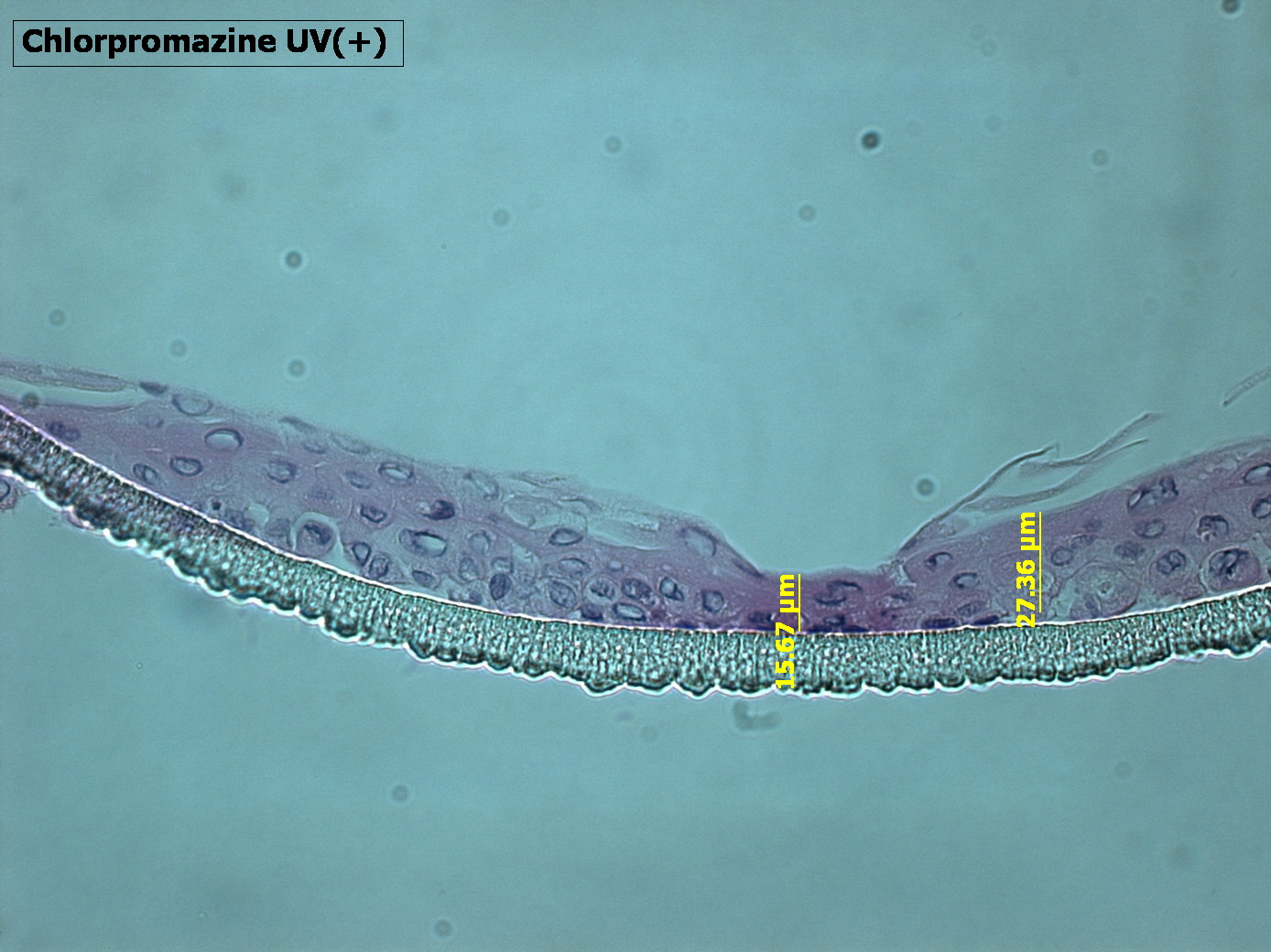

Supplement: S1 Fig — (ZIP) [file pone.0196735.s004.zip › HCM(H&E) staning raw data/HCM/Cornea_C+/SNAP-104710-0063-1.jpg]

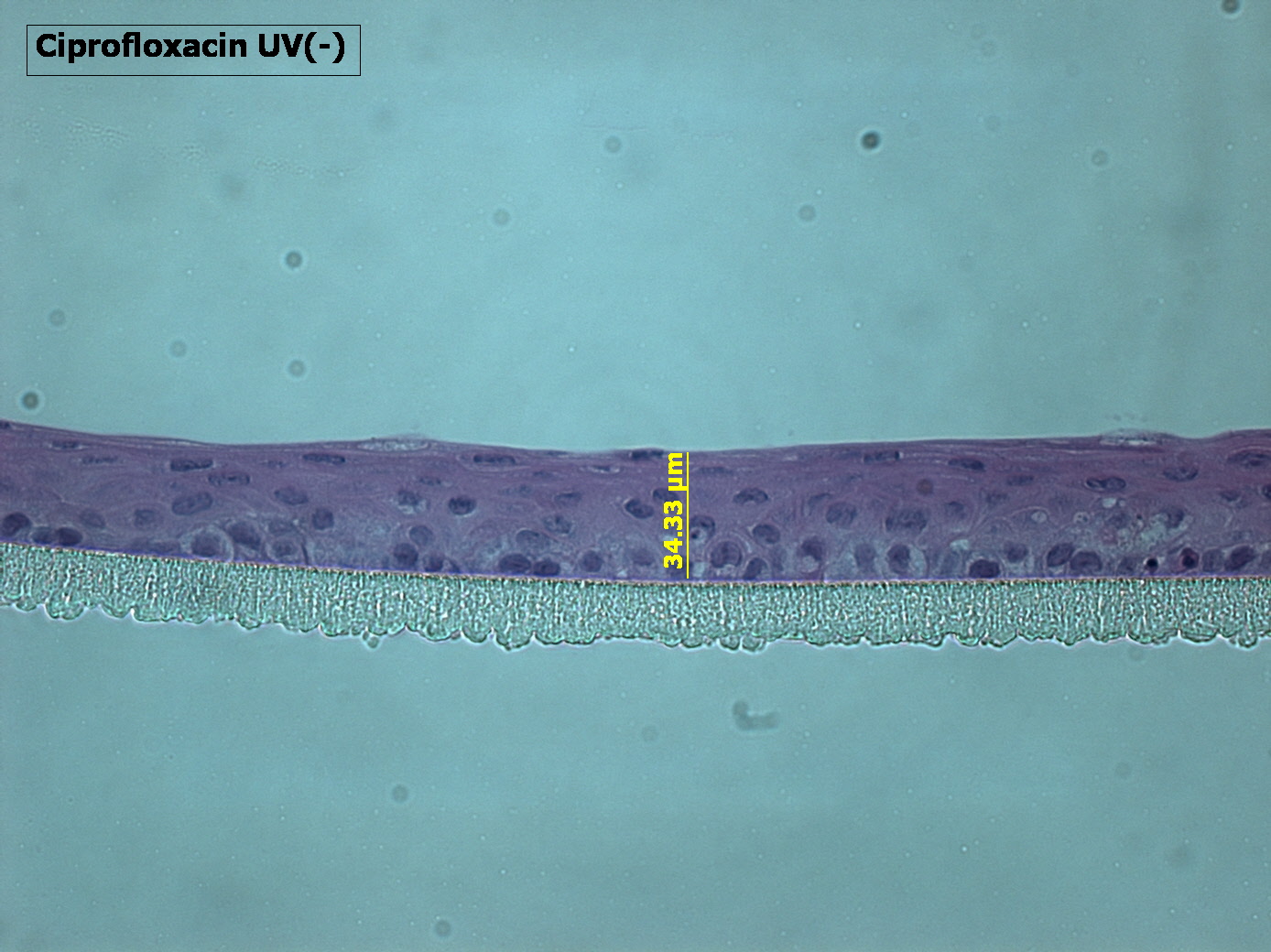

Supplement: S1 Fig — (ZIP) [file pone.0196735.s004.zip › HCM(H&E) staning raw data/HCM/Cornea_Ci-/SNAP-104924-0066_1.jpg]

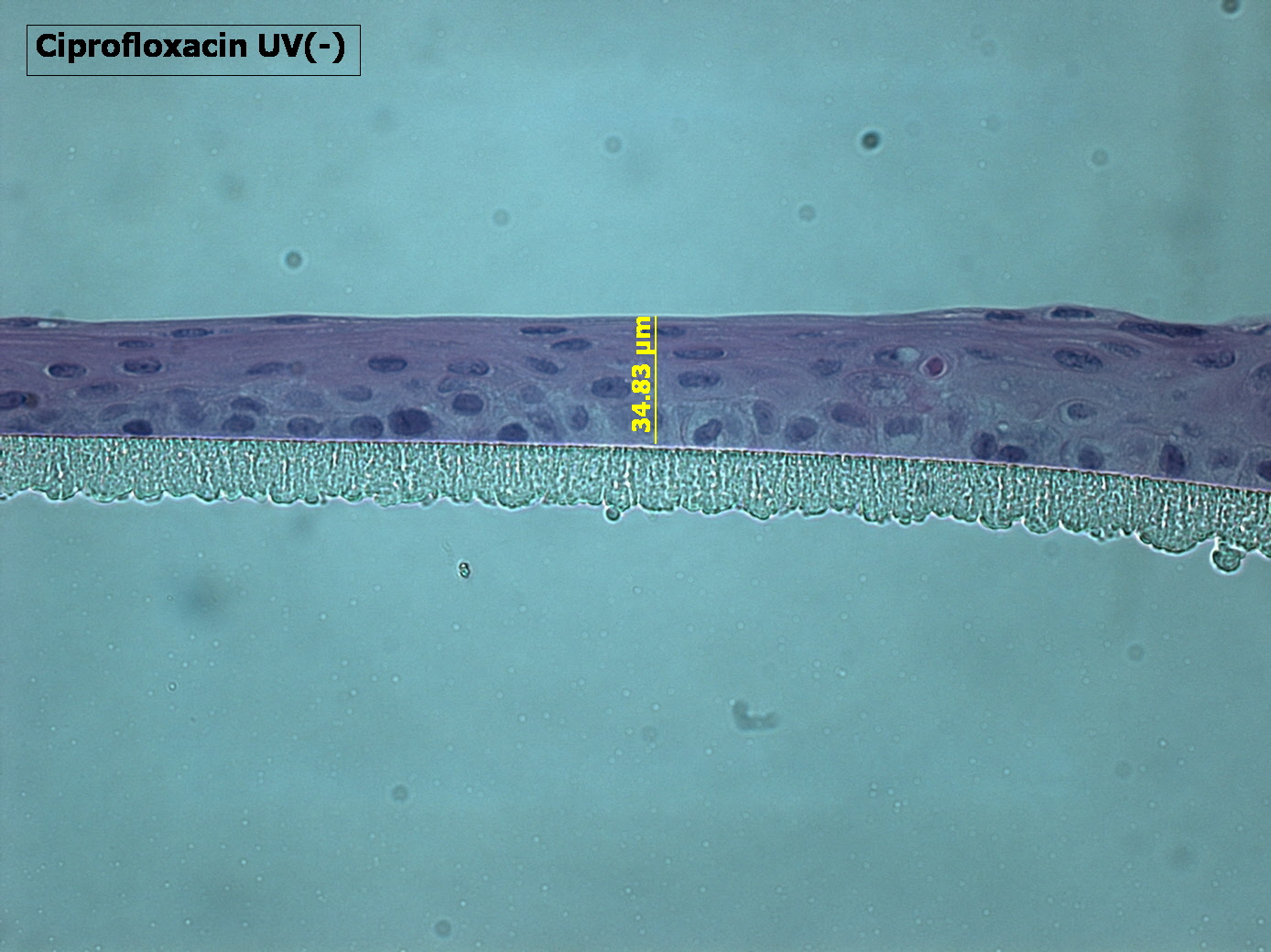

Supplement: S1 Fig — (ZIP) [file pone.0196735.s004.zip › HCM(H&E) staning raw data/HCM/Cornea_Ci-/SNAP-105221-0073_1.jpg]

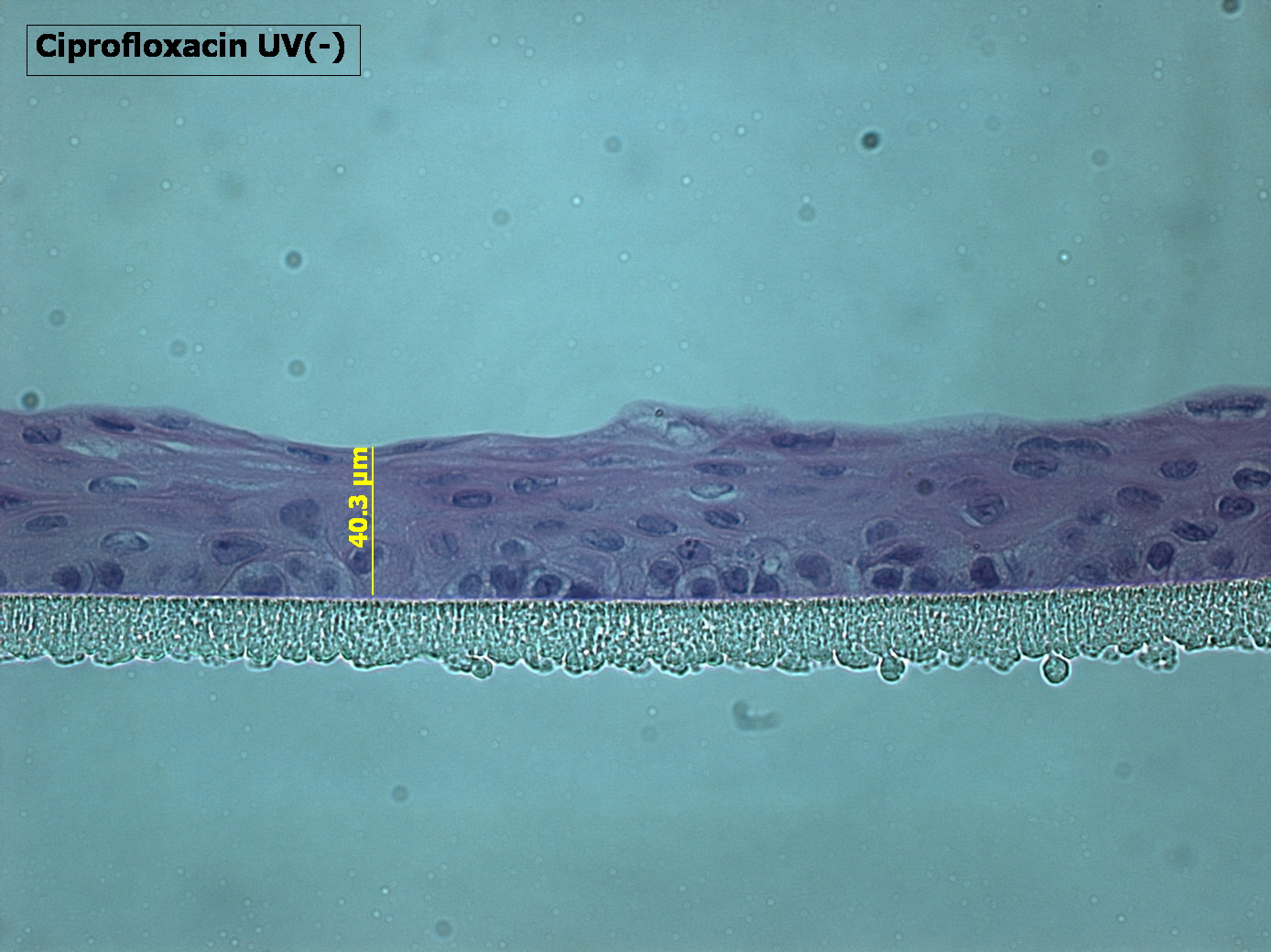

Supplement: S1 Fig — (ZIP) [file pone.0196735.s004.zip › HCM(H&E) staning raw data/HCM/Cornea_Ci-/SNAP-105505-0076_1.jpg]

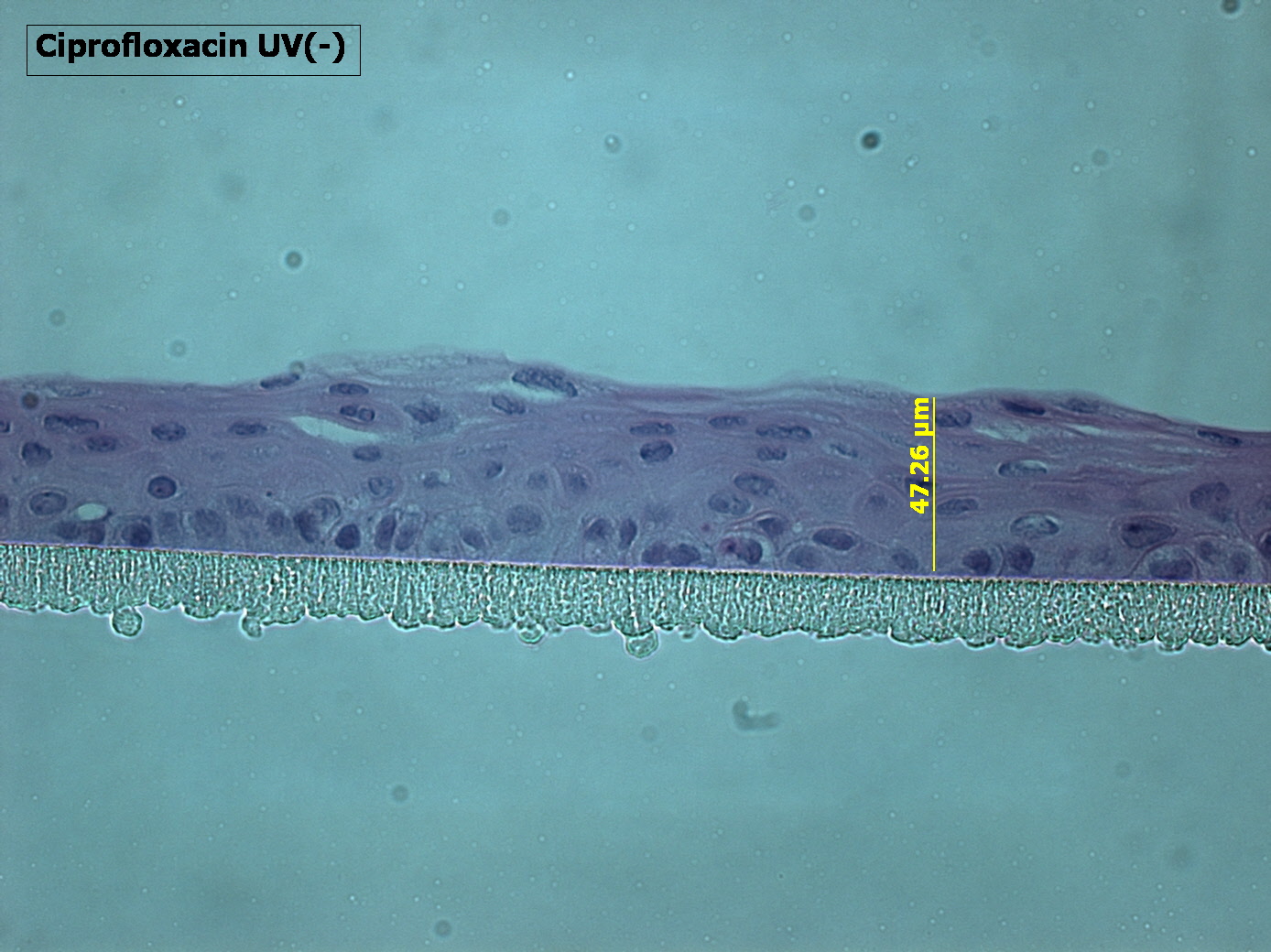

Supplement: S1 Fig — (ZIP) [file pone.0196735.s004.zip › HCM(H&E) staning raw data/HCM/Cornea_Ci-/SNAP-105515-0077_1.jpg]

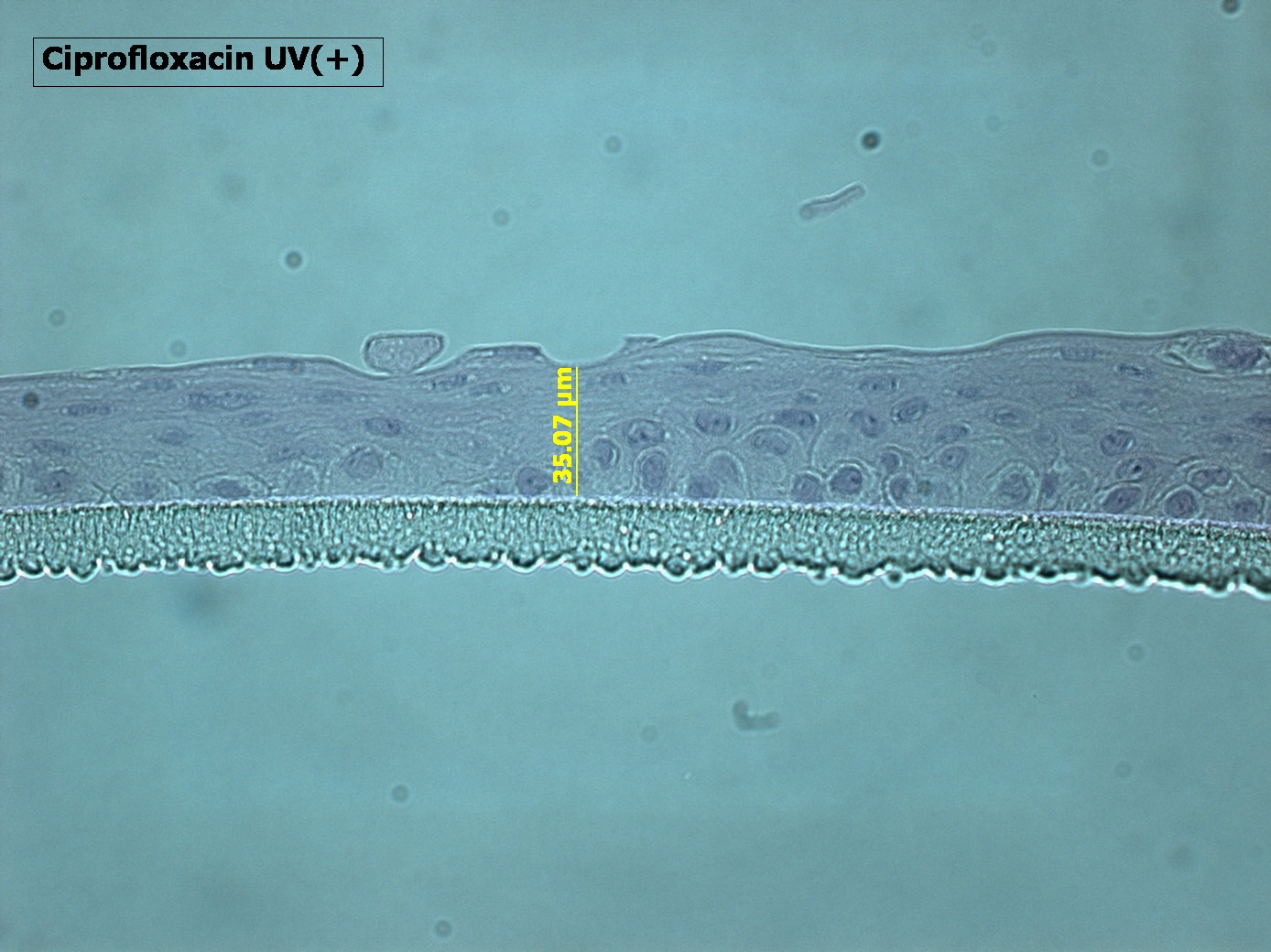

Supplement: S1 Fig — (ZIP) [file pone.0196735.s004.zip › HCM(H&E) staning raw data/HCM/Cornea_Ci+/SNAP-110915-0094_1.jpg]

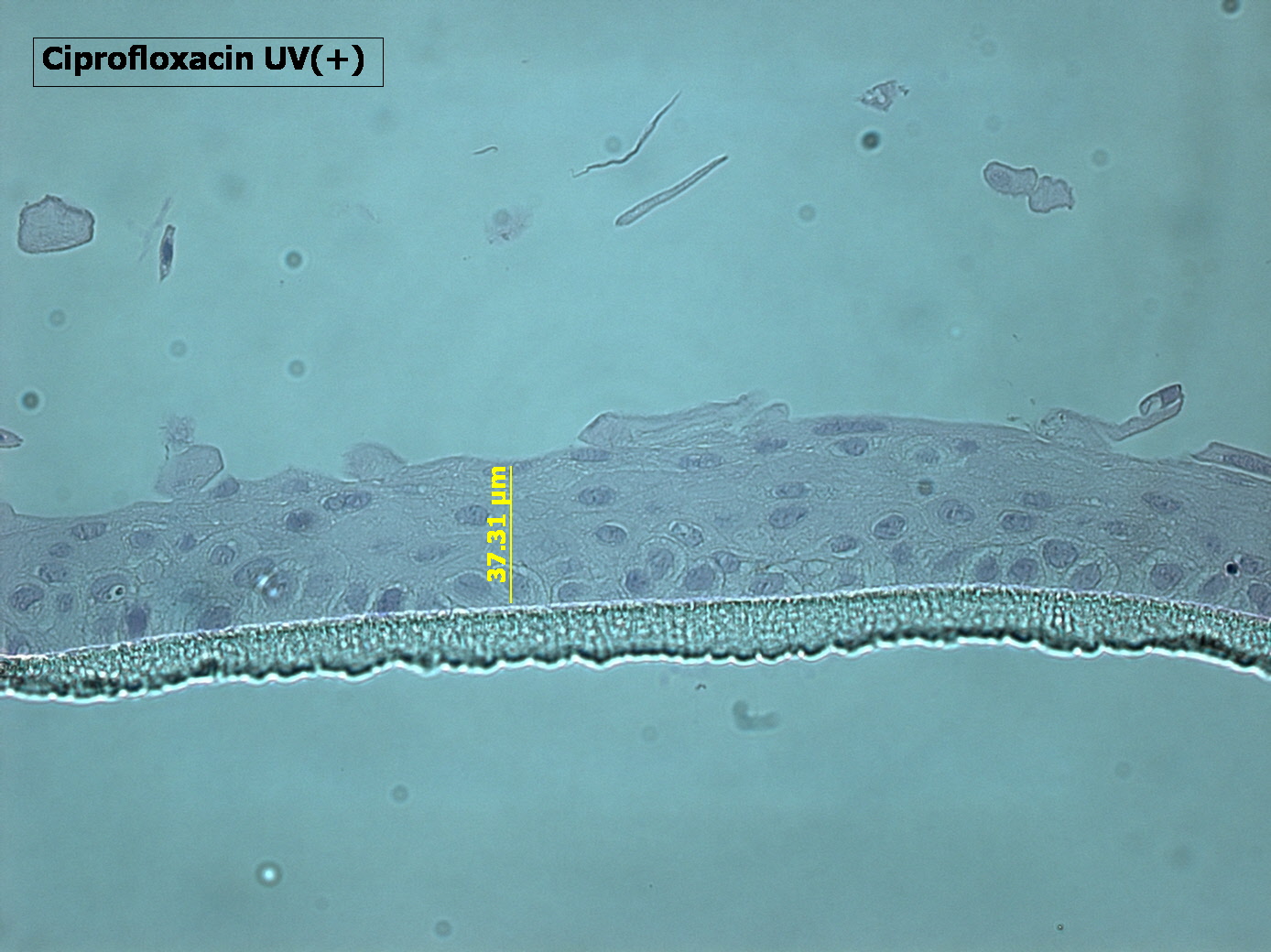

Supplement: S1 Fig — (ZIP) [file pone.0196735.s004.zip › HCM(H&E) staning raw data/HCM/Cornea_Ci+/SNAP-111131-0096_1.jpg]

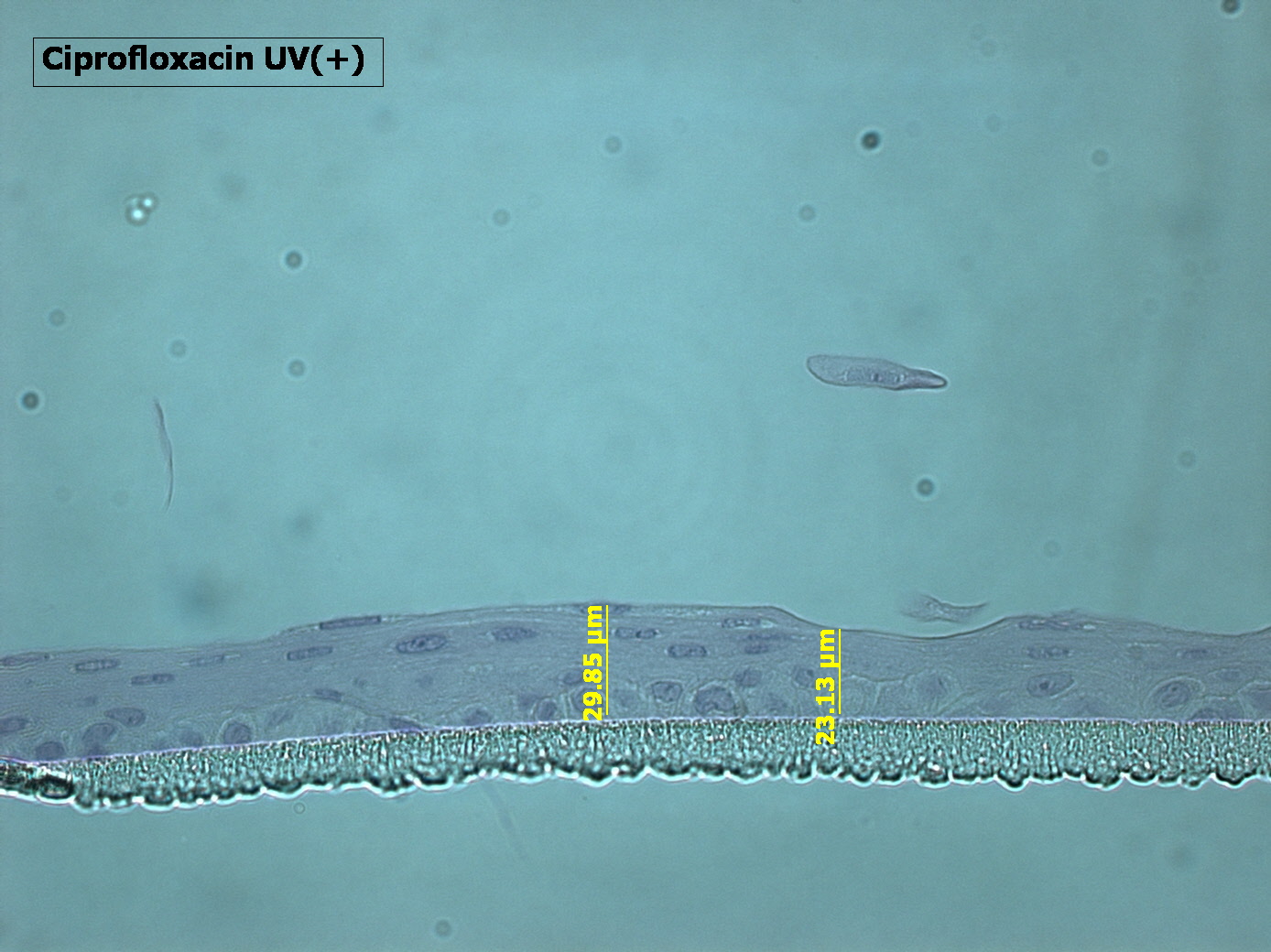

Supplement: S1 Fig — (ZIP) [file pone.0196735.s004.zip › HCM(H&E) staning raw data/HCM/Cornea_Ci+/SNAP-111322-0099_1.jpg]

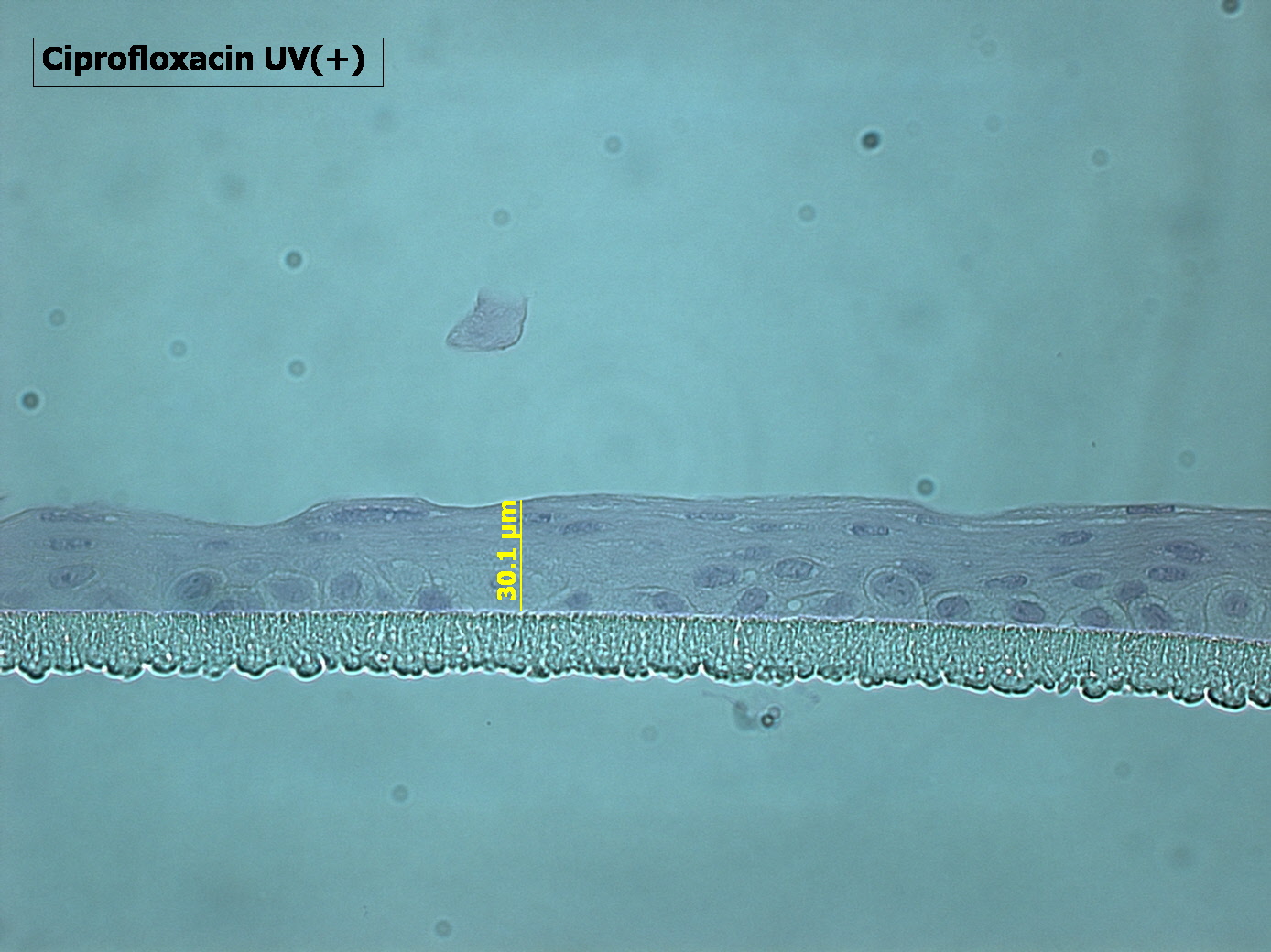

Supplement: S1 Fig — (ZIP) [file pone.0196735.s004.zip › HCM(H&E) staning raw data/HCM/Cornea_Ci+/SNAP-111331-0100_1.jpg]

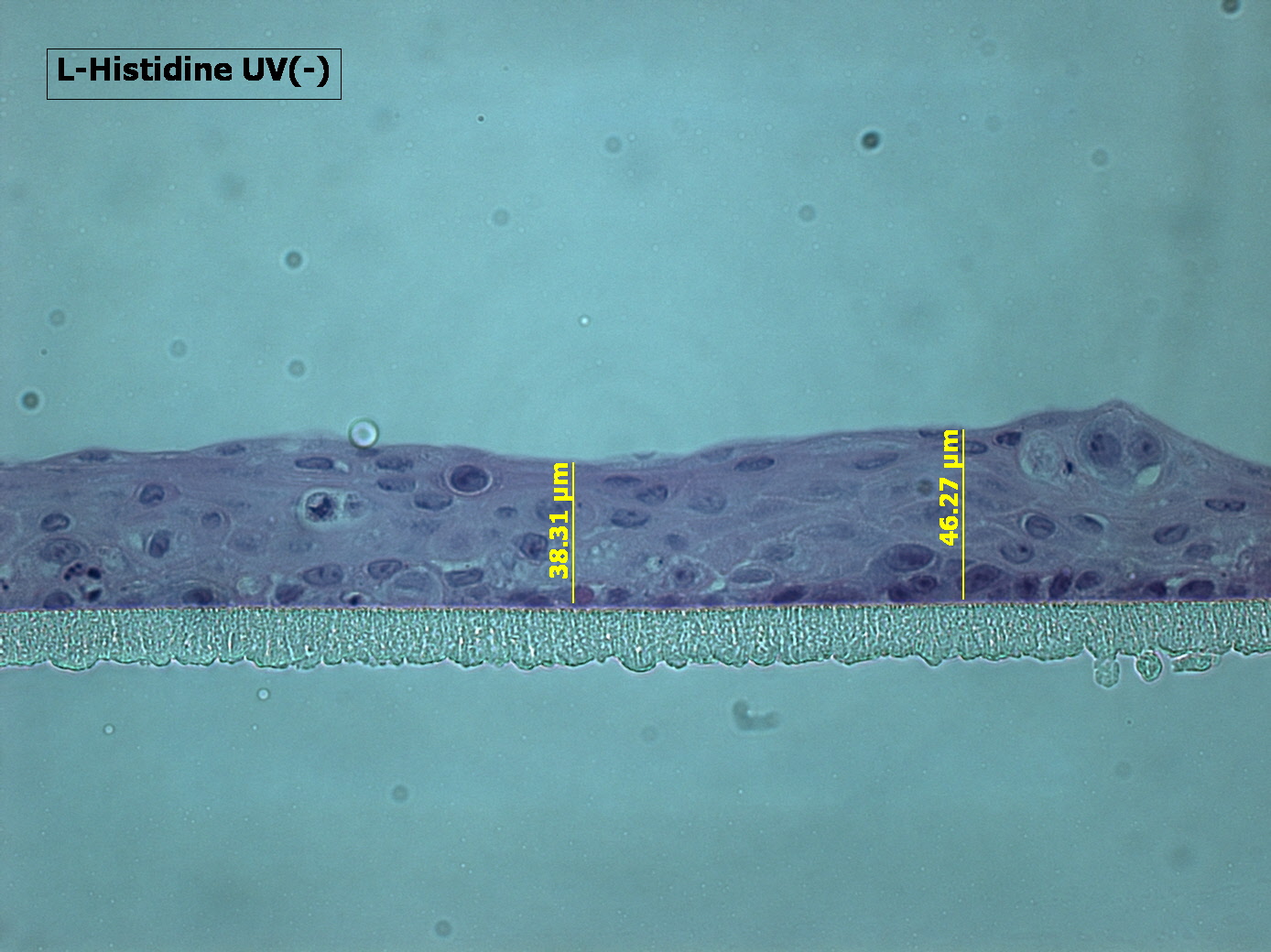

Supplement: S1 Fig — (ZIP) [file pone.0196735.s004.zip › HCM(H&E) staning raw data/HCM/Cornea_L-/SNAP-112619-0107_1.jpg]

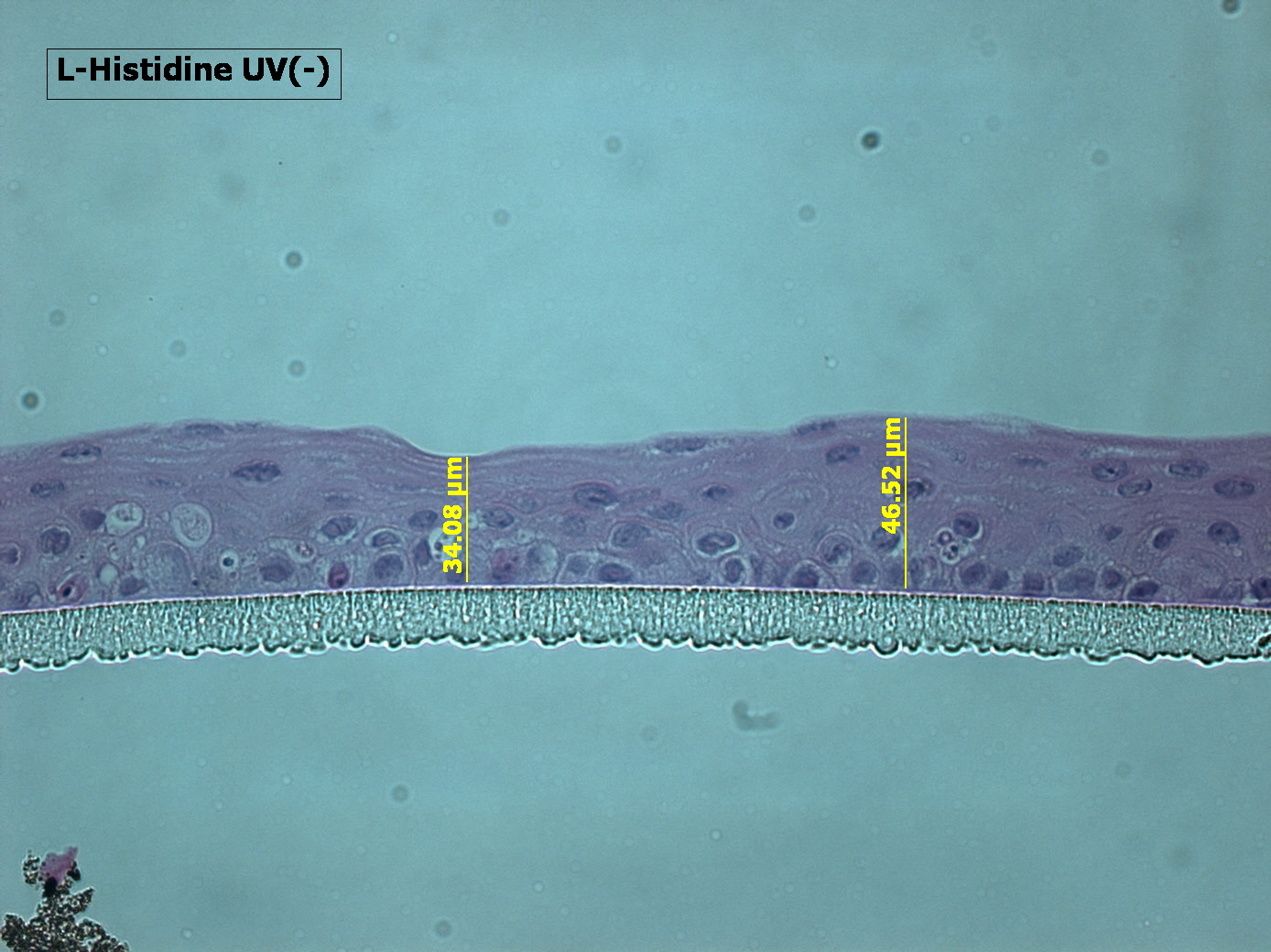

Supplement: S1 Fig — (ZIP) [file pone.0196735.s004.zip › HCM(H&E) staning raw data/HCM/Cornea_L-/SNAP-113140-0109_1.jpg]

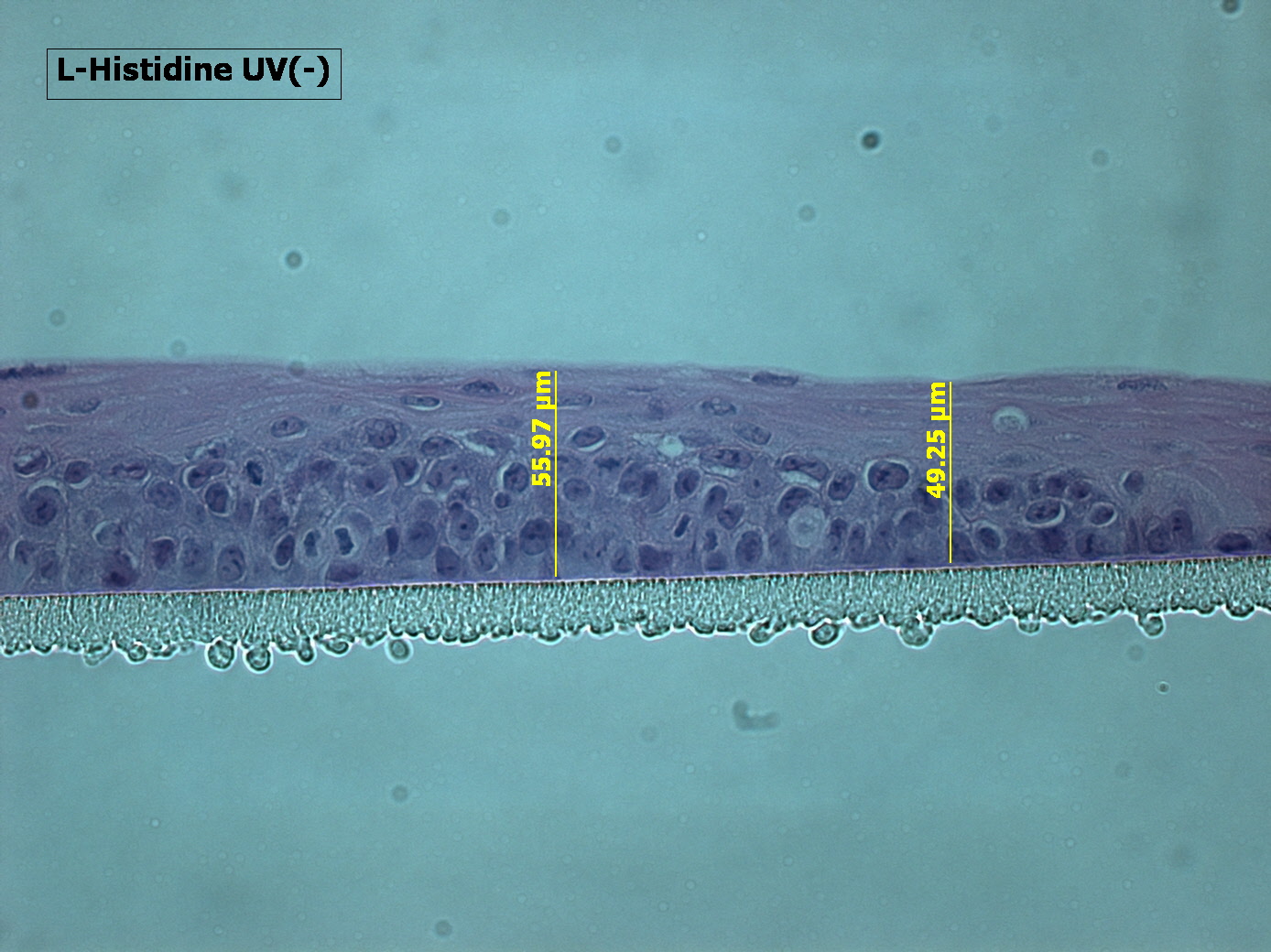

Supplement: S1 Fig — (ZIP) [file pone.0196735.s004.zip › HCM(H&E) staning raw data/HCM/Cornea_L-/SNAP-113243-0111_1.jpg]

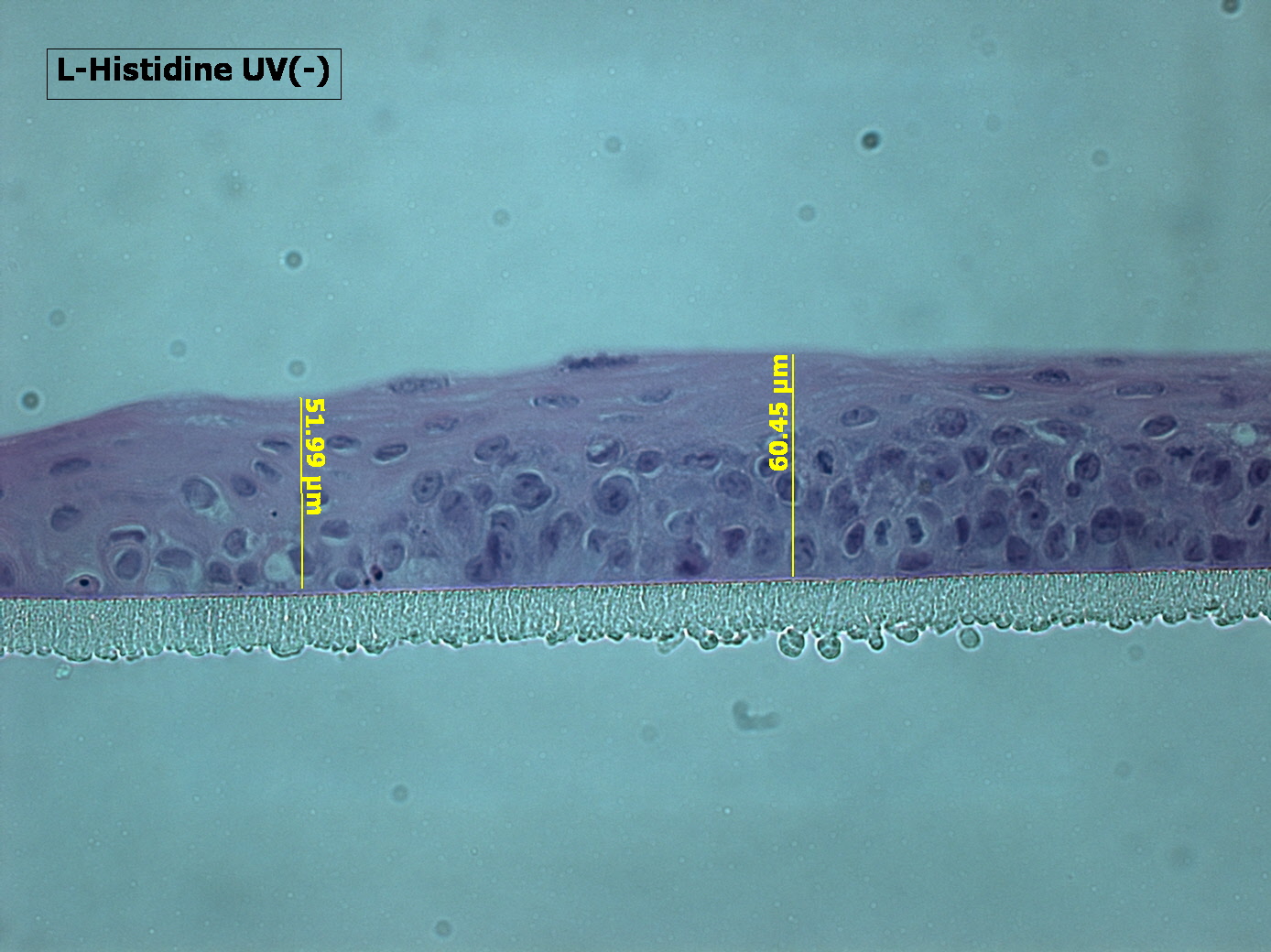

Supplement: S1 Fig — (ZIP) [file pone.0196735.s004.zip › HCM(H&E) staning raw data/HCM/Cornea_L-/SNAP-113248-0112_1.jpg]

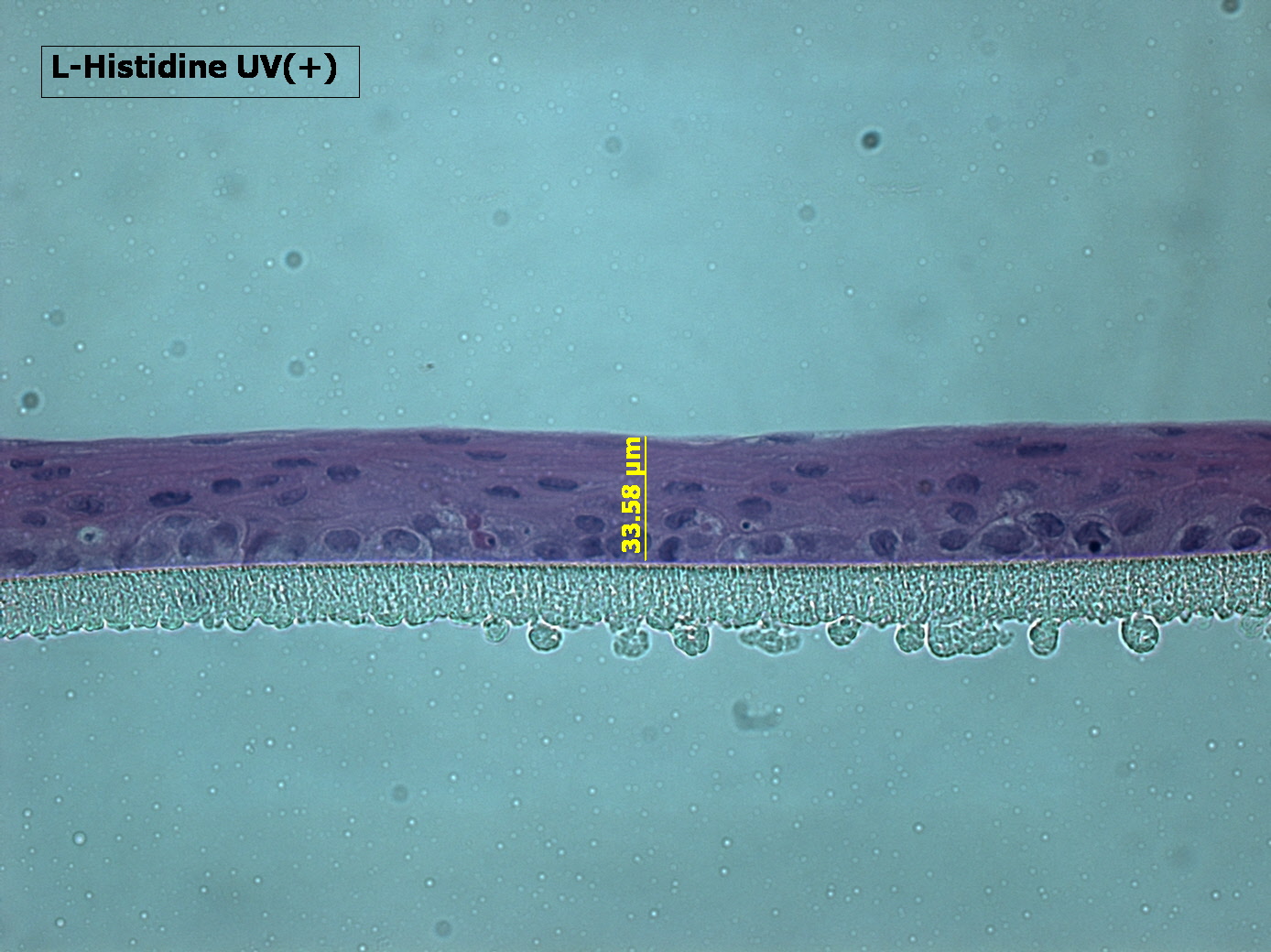

Supplement: S1 Fig — (ZIP) [file pone.0196735.s004.zip › HCM(H&E) staning raw data/HCM/Cornea_L+/SNAP-113743-0117_1.jpg]

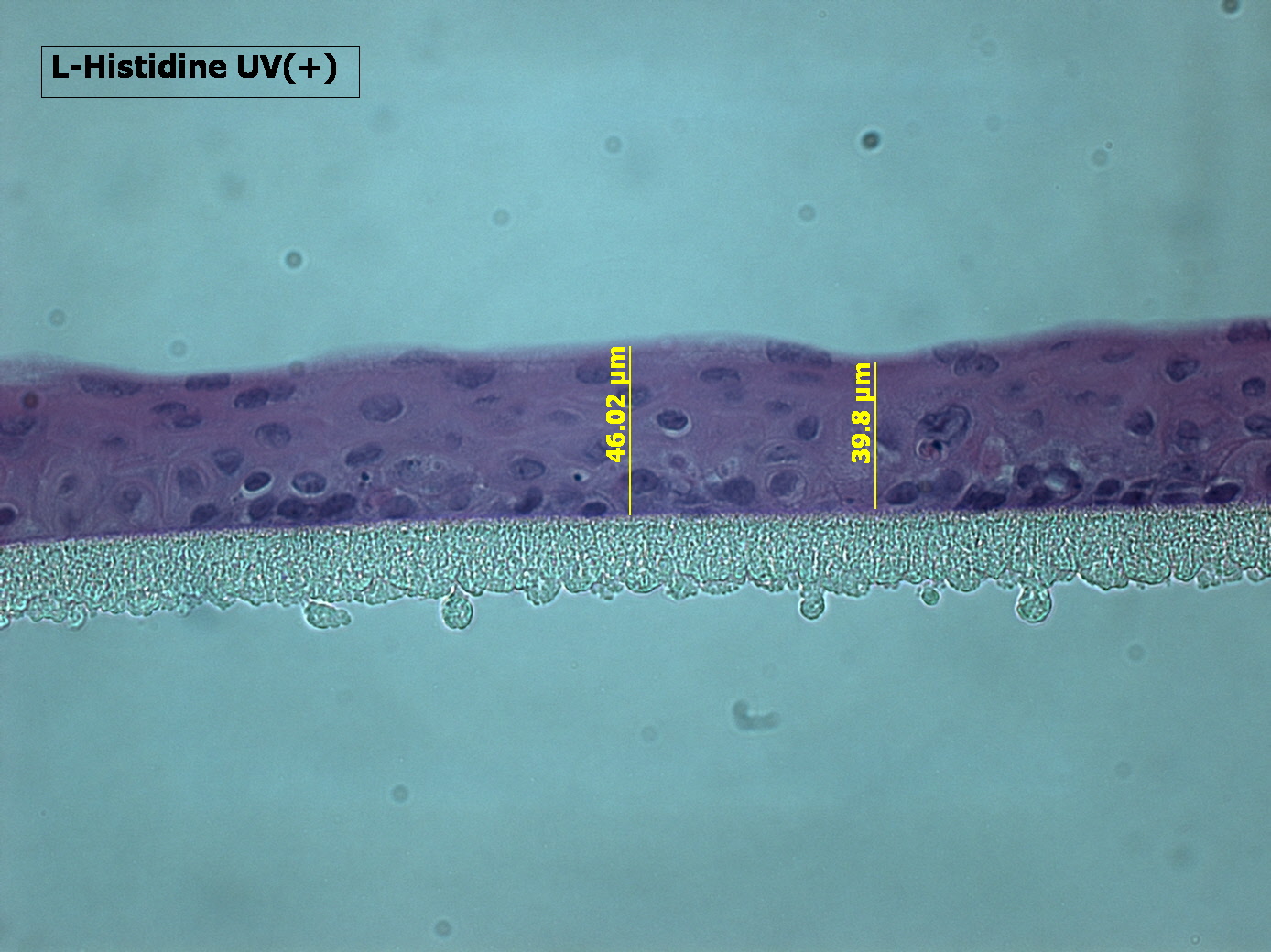

Supplement: S1 Fig — (ZIP) [file pone.0196735.s004.zip › HCM(H&E) staning raw data/HCM/Cornea_L+/SNAP-113936-0124_1.jpg]

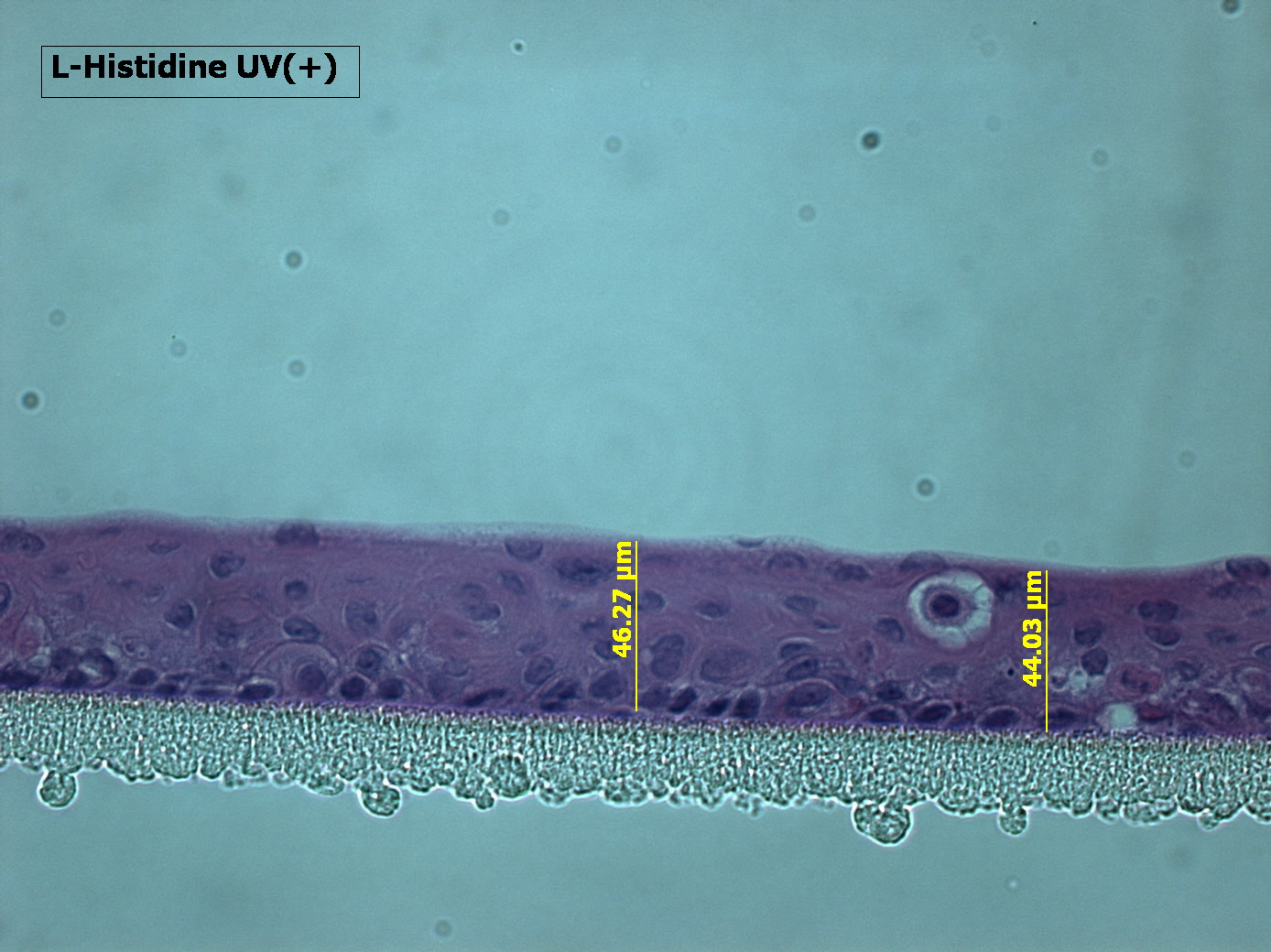

Supplement: S1 Fig — (ZIP) [file pone.0196735.s004.zip › HCM(H&E) staning raw data/HCM/Cornea_L+/SNAP-114053-0128_1.jpg]

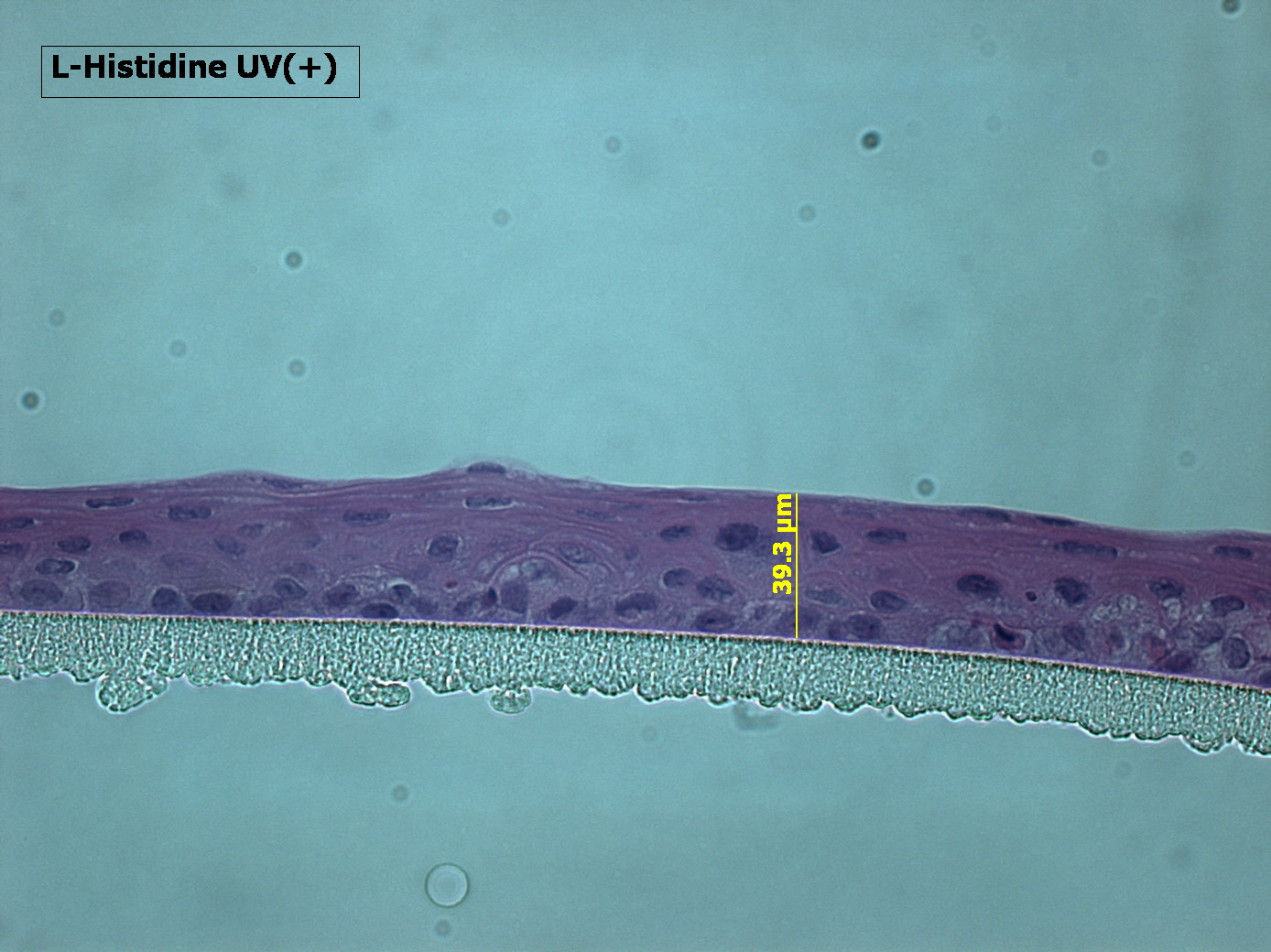

Supplement: S1 Fig — (ZIP) [file pone.0196735.s004.zip › HCM(H&E) staning raw data/HCM/Cornea_L+/SNAP-114220-0133_1.jpg]

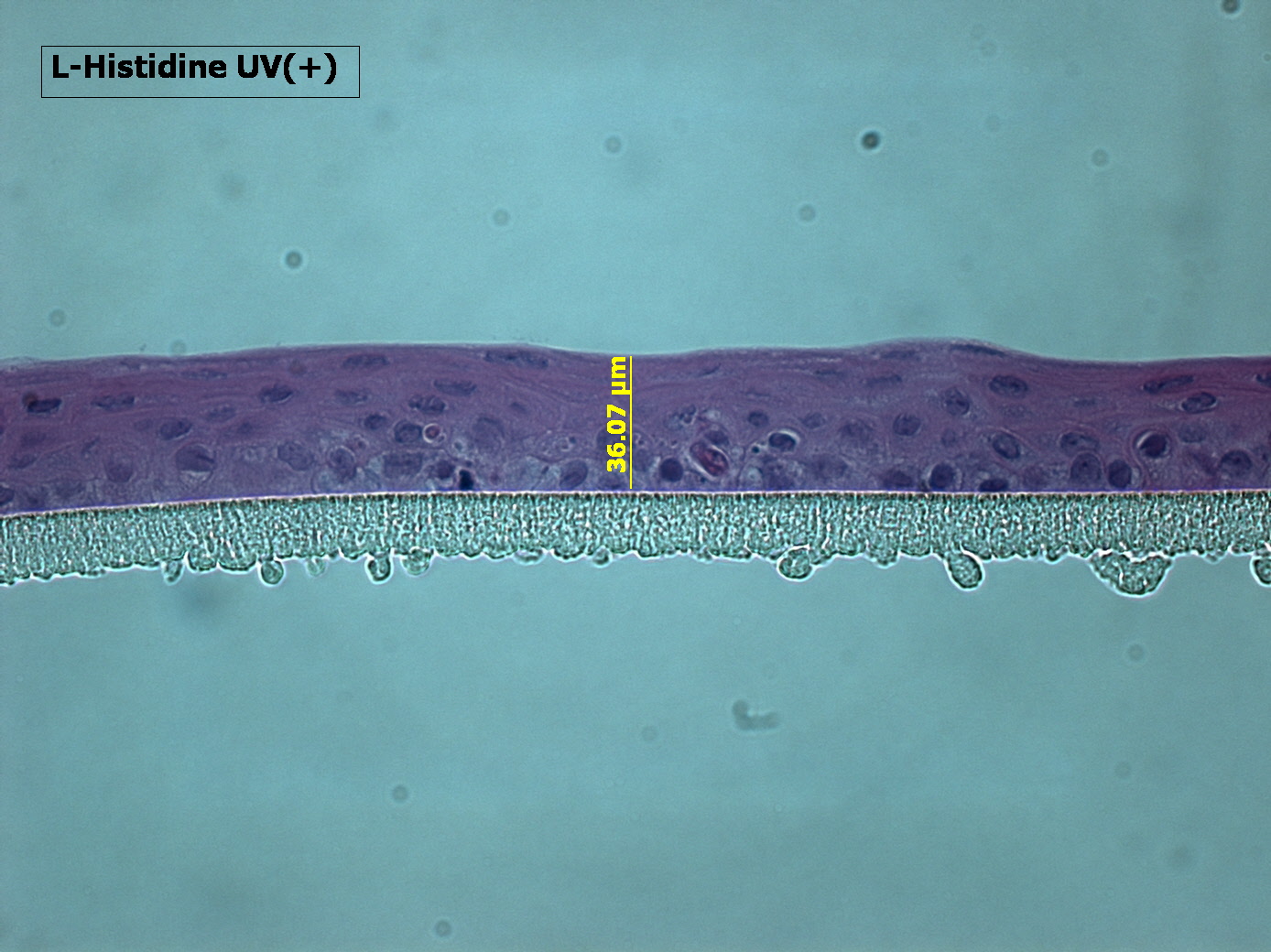

Supplement: S1 Fig — (ZIP) [file pone.0196735.s004.zip › HCM(H&E) staning raw data/HCM/Cornea_L+/SNAP-114521-0136_1.jpg]

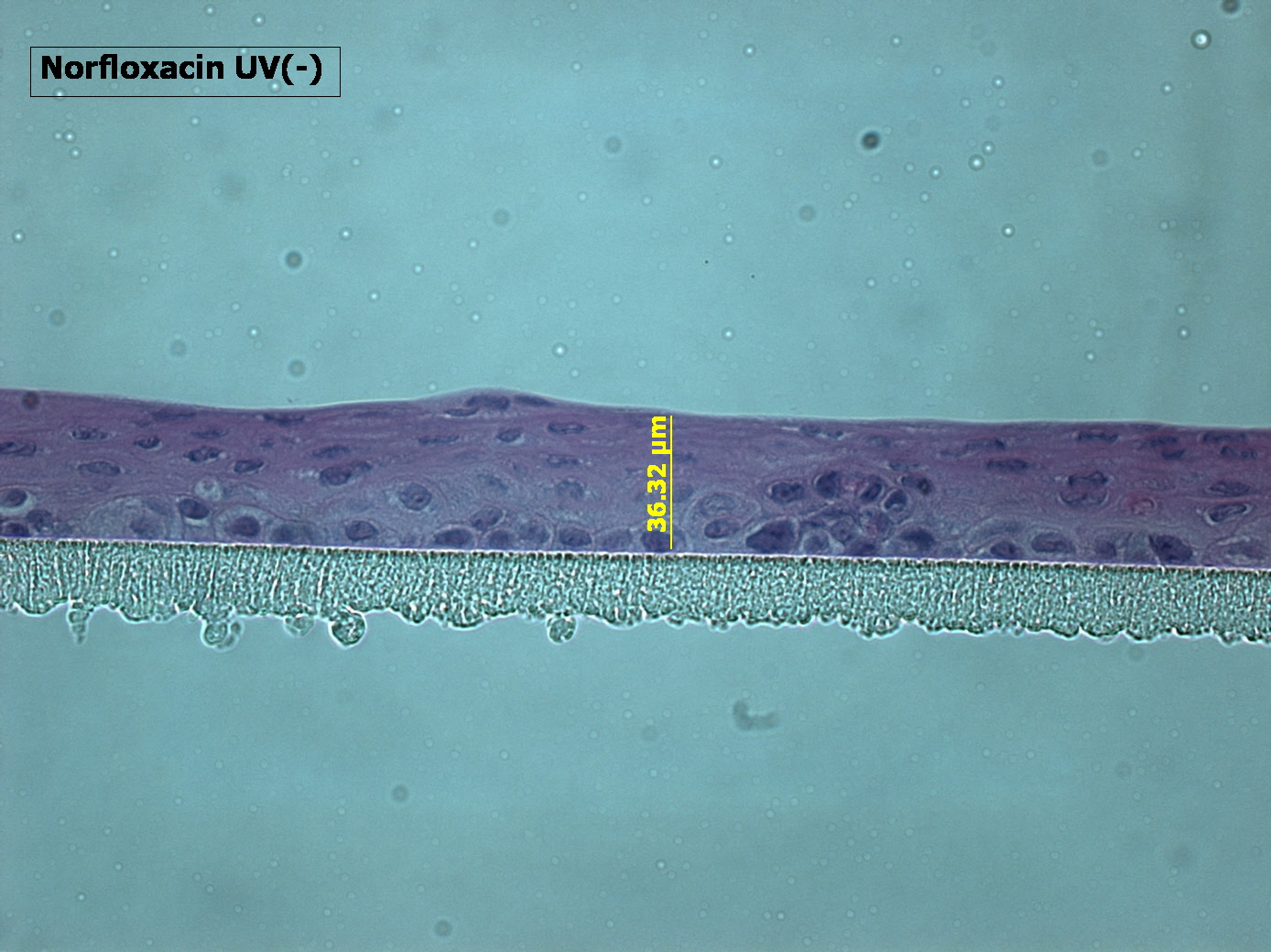

Supplement: S1 Fig — (ZIP) [file pone.0196735.s004.zip › HCM(H&E) staning raw data/HCM/Cornea_N-/SNAP-115253-0145_1.jpg]

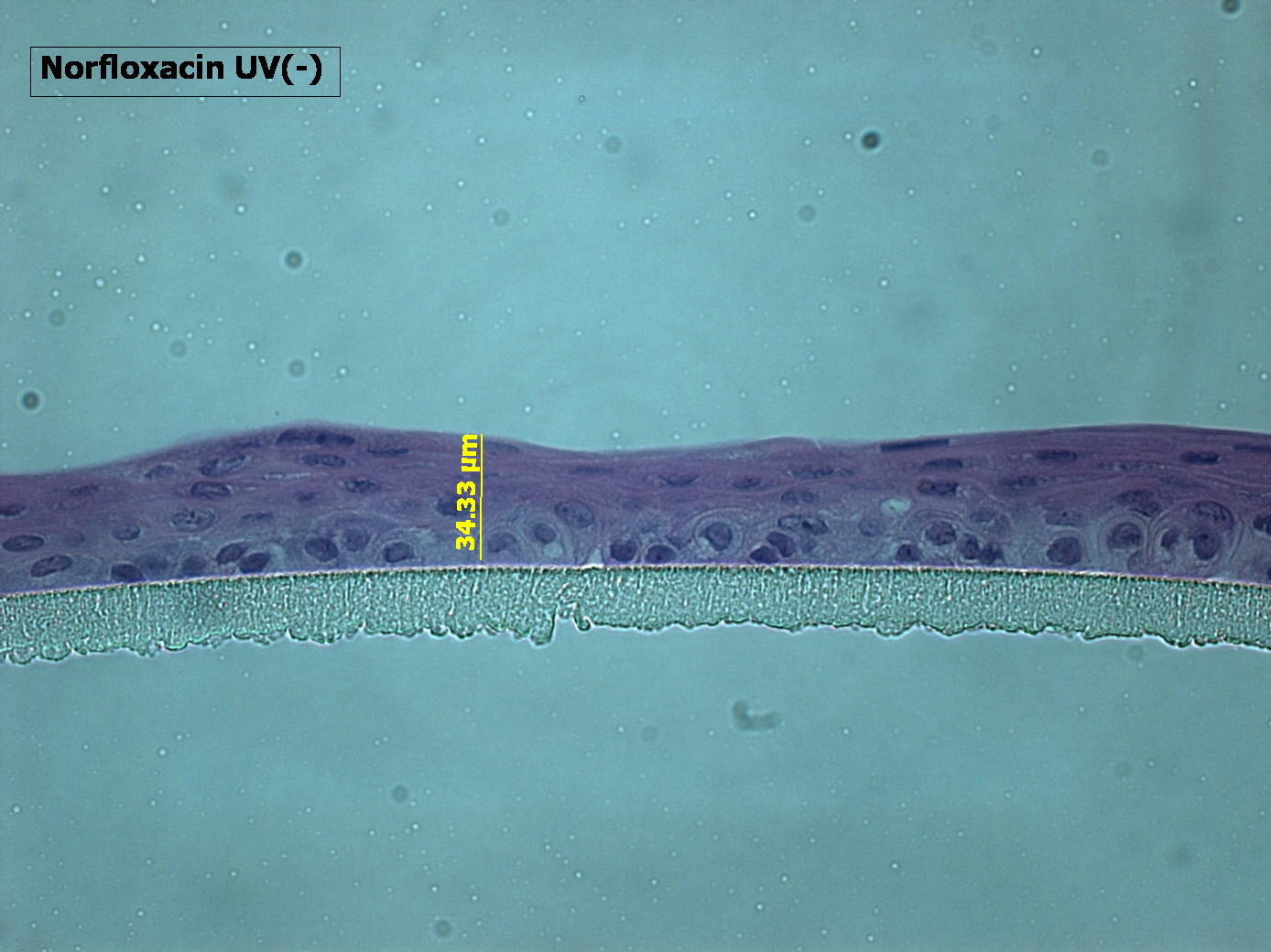

Supplement: S1 Fig — (ZIP) [file pone.0196735.s004.zip › HCM(H&E) staning raw data/HCM/Cornea_N-/SNAP-115351-0149_1.jpg]

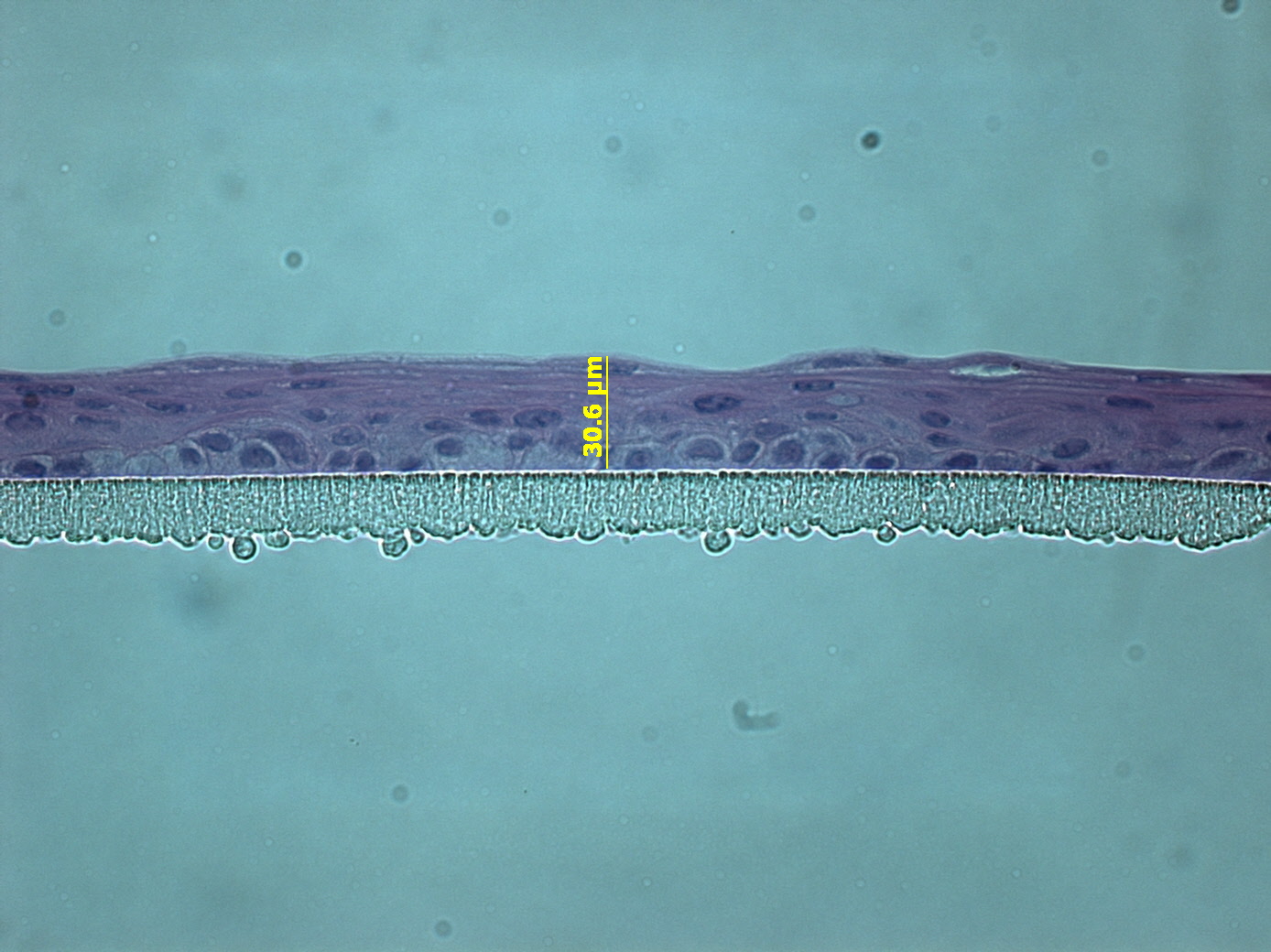

Supplement: S1 Fig — (ZIP) [file pone.0196735.s004.zip › HCM(H&E) staning raw data/HCM/Cornea_N-/SNAP-115444-0154_1.jpg]

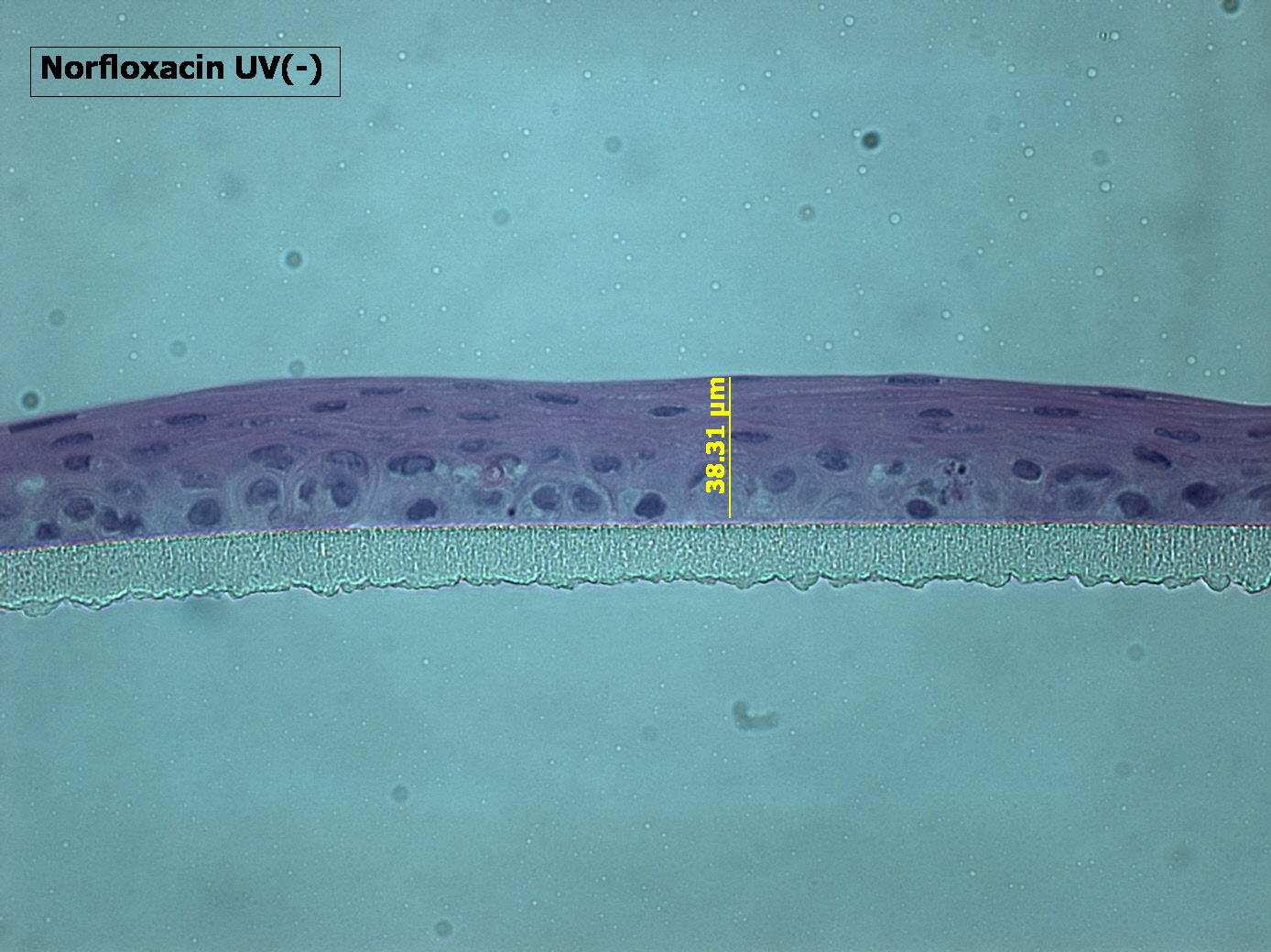

Supplement: S1 Fig — (ZIP) [file pone.0196735.s004.zip › HCM(H&E) staning raw data/HCM/Cornea_N-/SNAP-115912-0161_1.jpg]

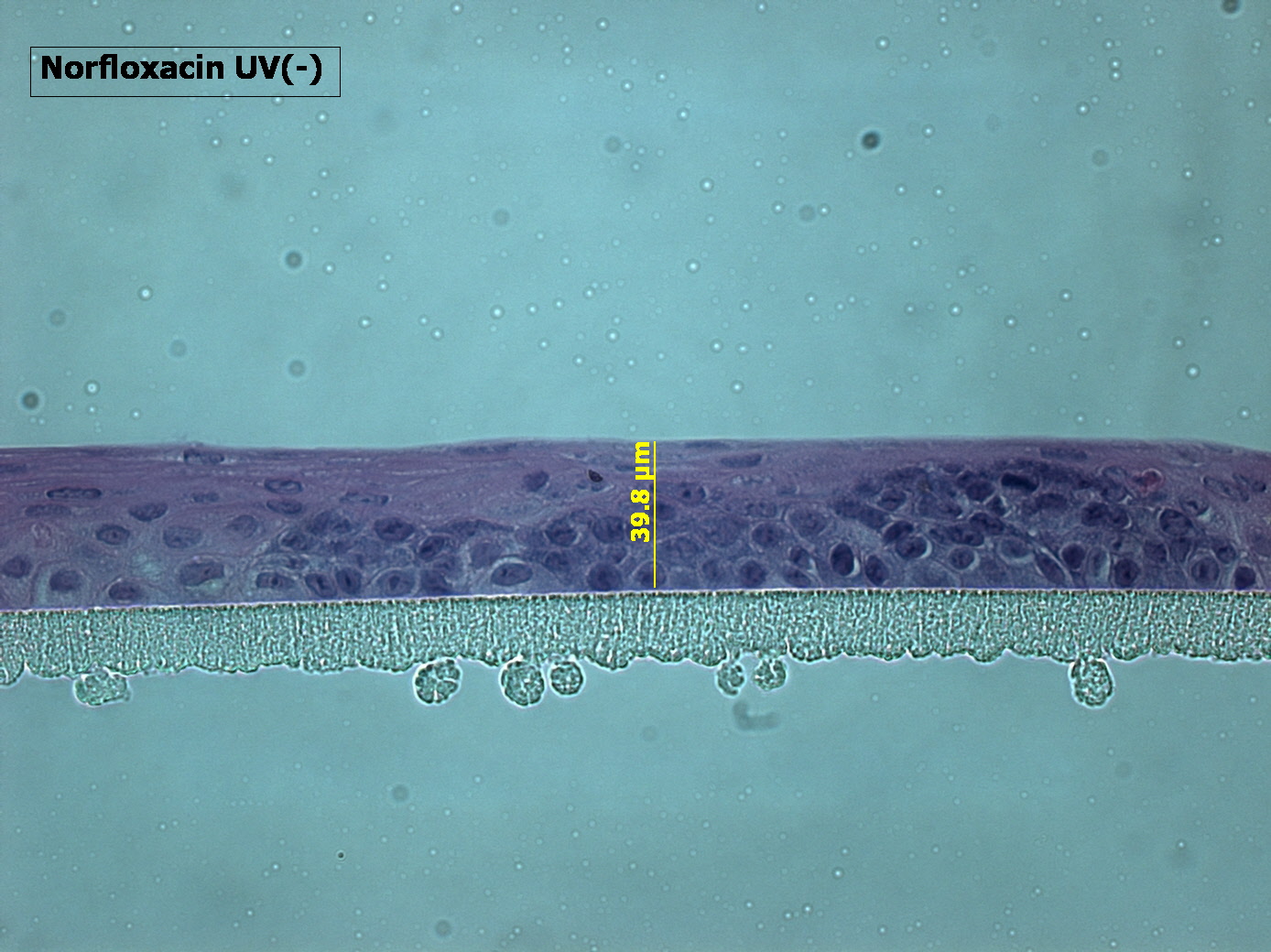

Supplement: S1 Fig — (ZIP) [file pone.0196735.s004.zip › HCM(H&E) staning raw data/HCM/Cornea_N-/SNAP-115939-0163_1.jpg]

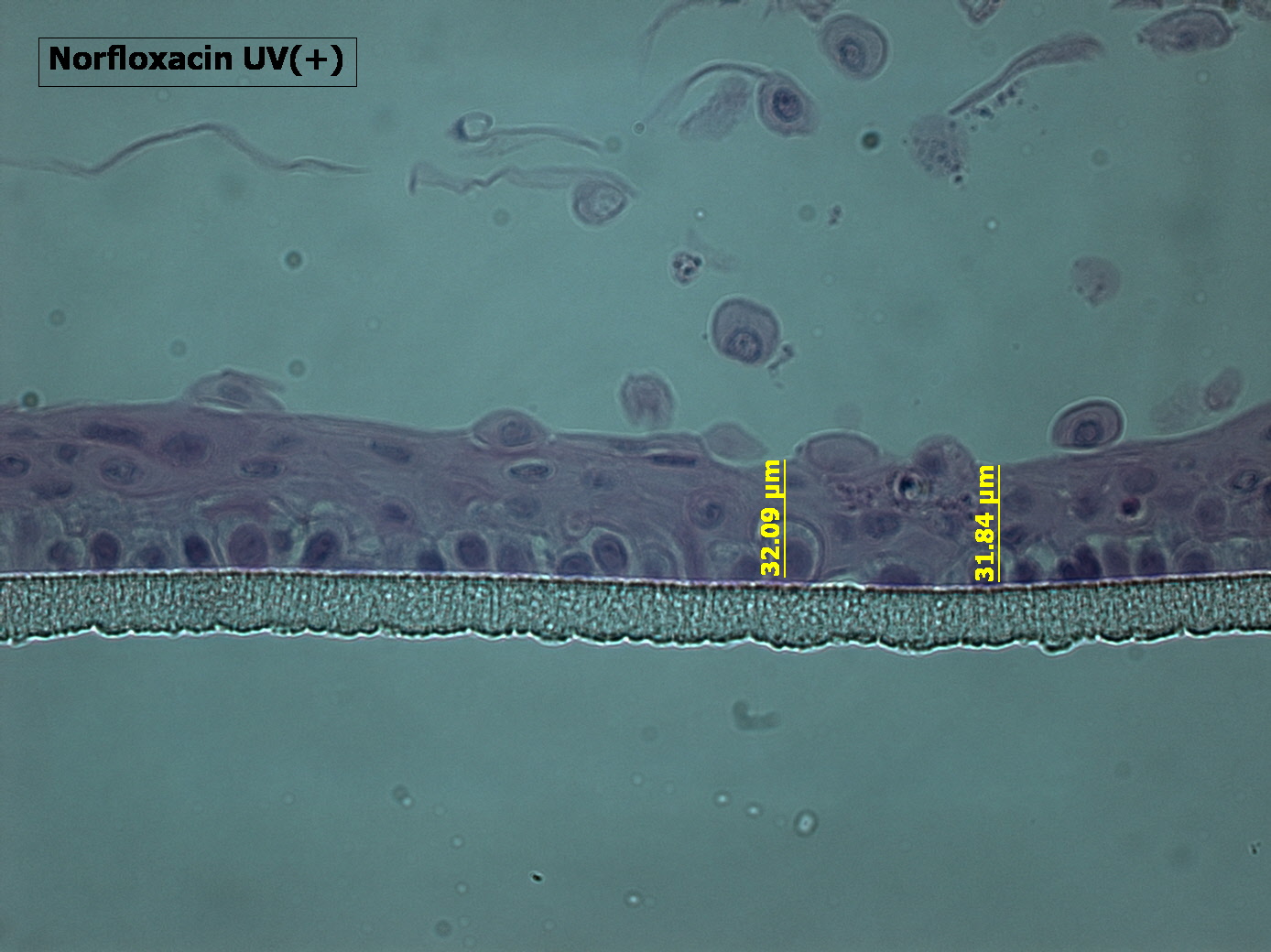

Supplement: S1 Fig — (ZIP) [file pone.0196735.s004.zip › HCM(H&E) staning raw data/HCM/Cornea_N+/SNAP-135426-0002_1.jpg]

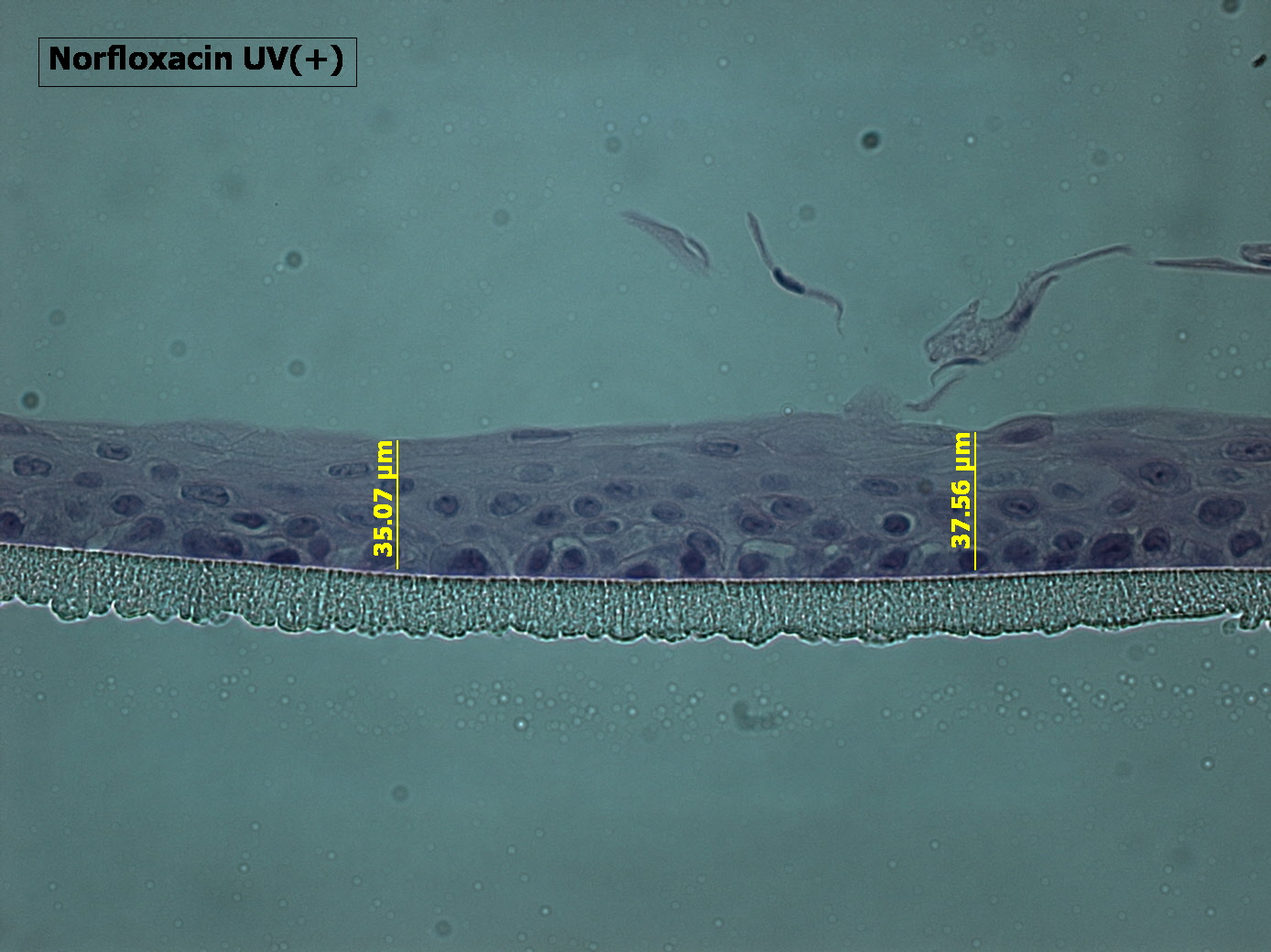

Supplement: S1 Fig — (ZIP) [file pone.0196735.s004.zip › HCM(H&E) staning raw data/HCM/Cornea_N+/SNAP-135827-0009_1.jpg]

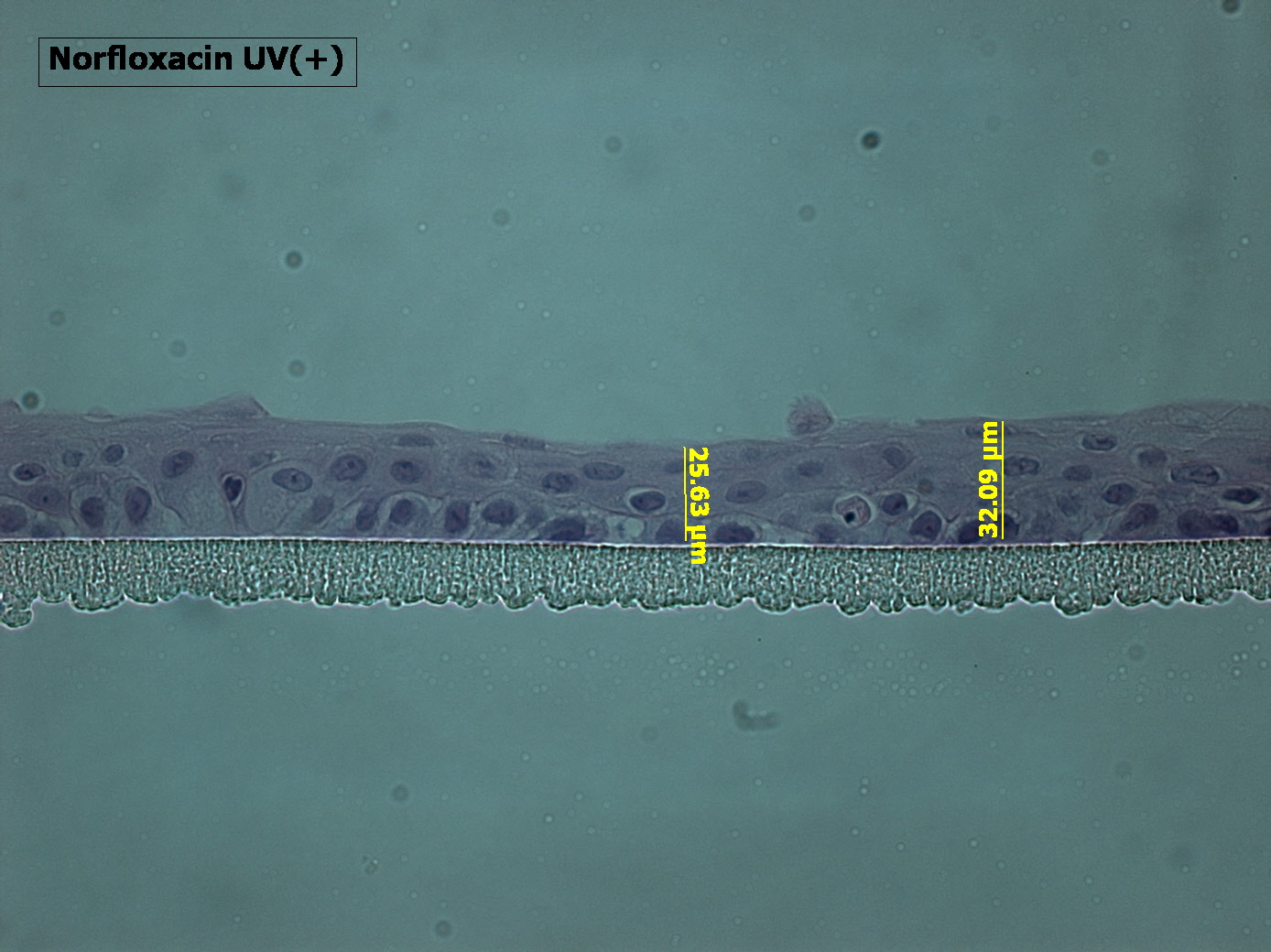

Supplement: S1 Fig — (ZIP) [file pone.0196735.s004.zip › HCM(H&E) staning raw data/HCM/Cornea_N+/SNAP-135909-0010_1.jpg]

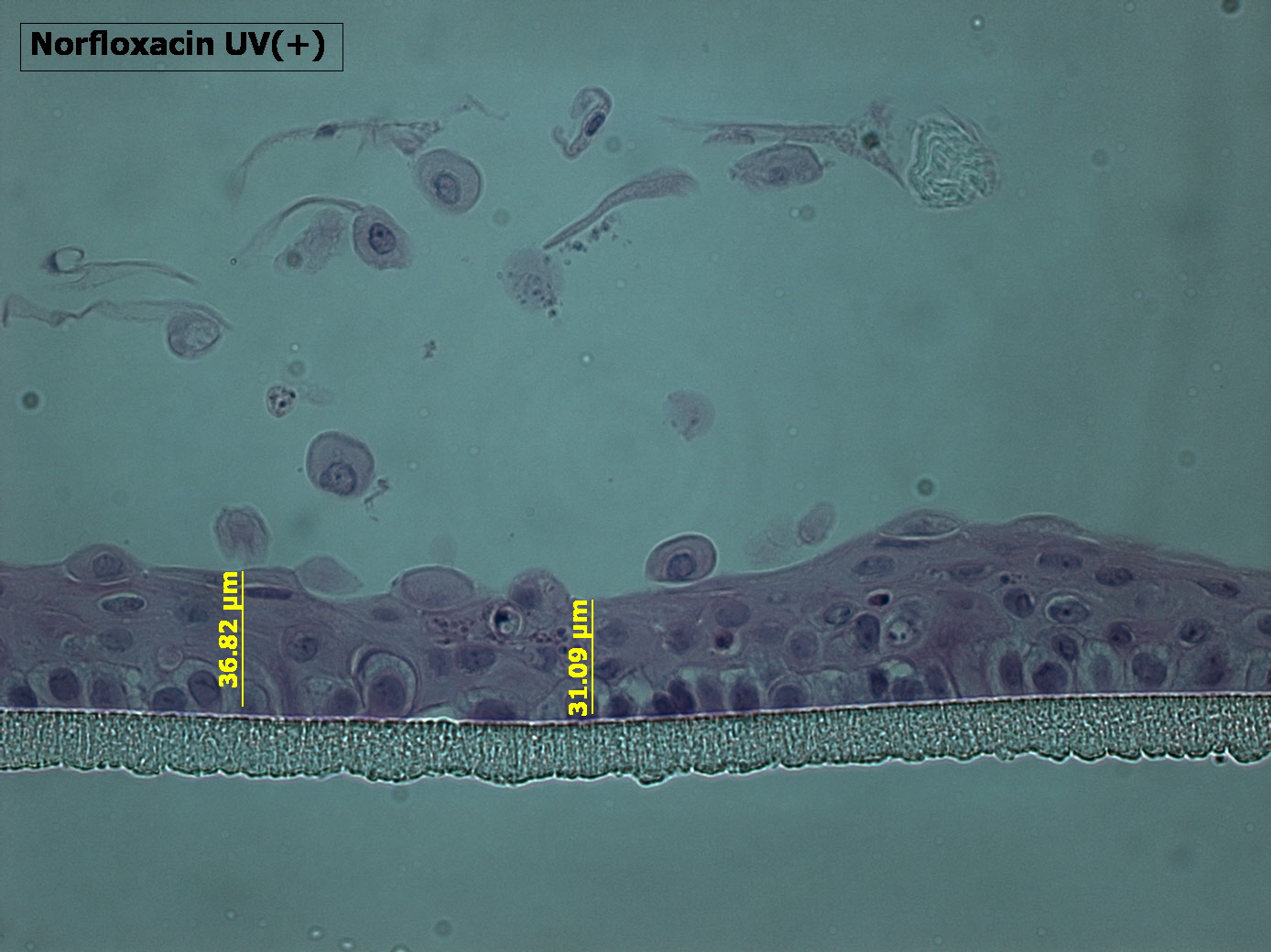

Supplement: S1 Fig — (ZIP) [file pone.0196735.s004.zip › HCM(H&E) staning raw data/HCM/Cornea_N+/SNAP-135925-0011_1.jpg]

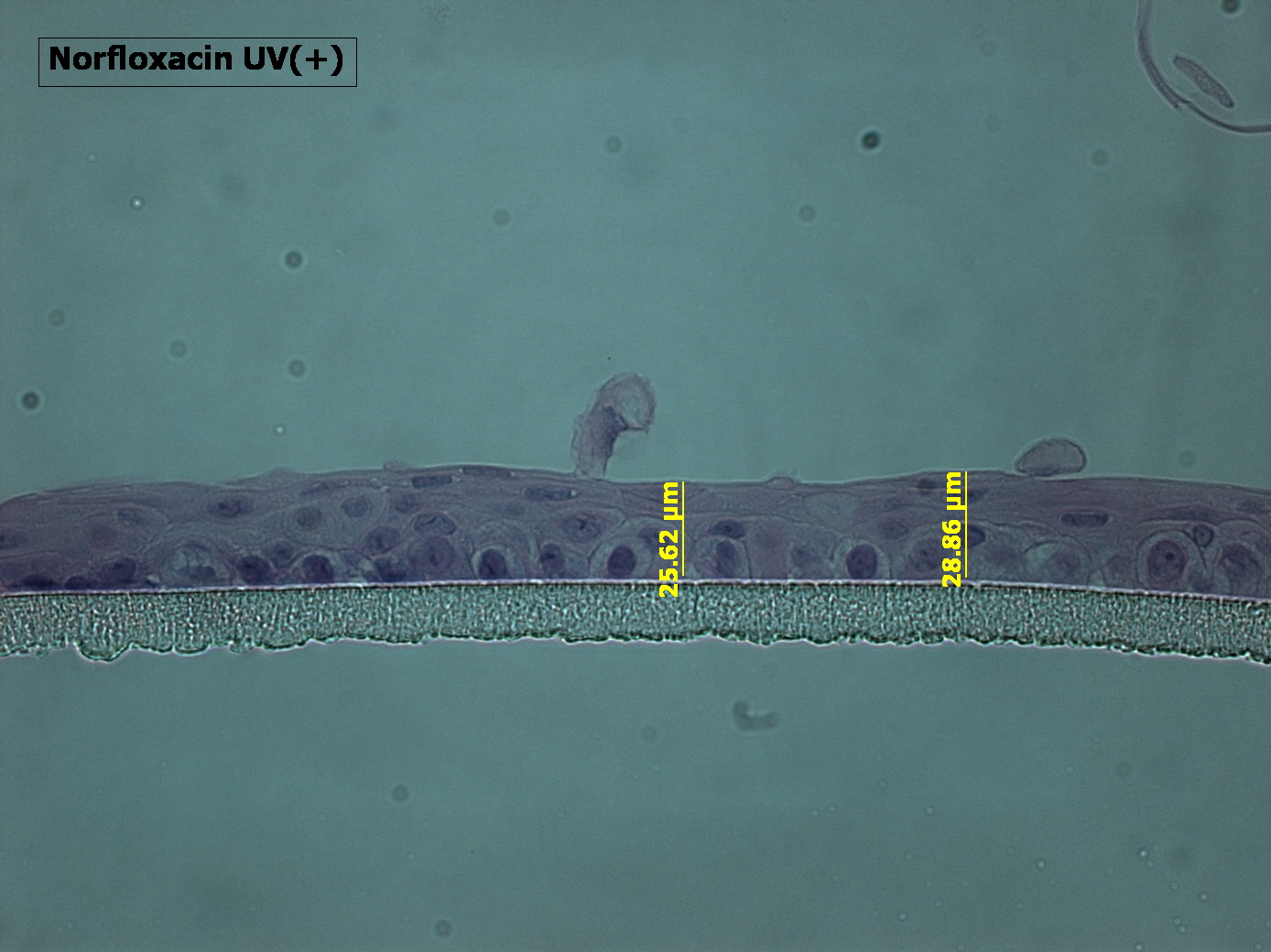

Supplement: S1 Fig — (ZIP) [file pone.0196735.s004.zip › HCM(H&E) staning raw data/HCM/Cornea_N+/SNAP-140119-0013_1.jpg]

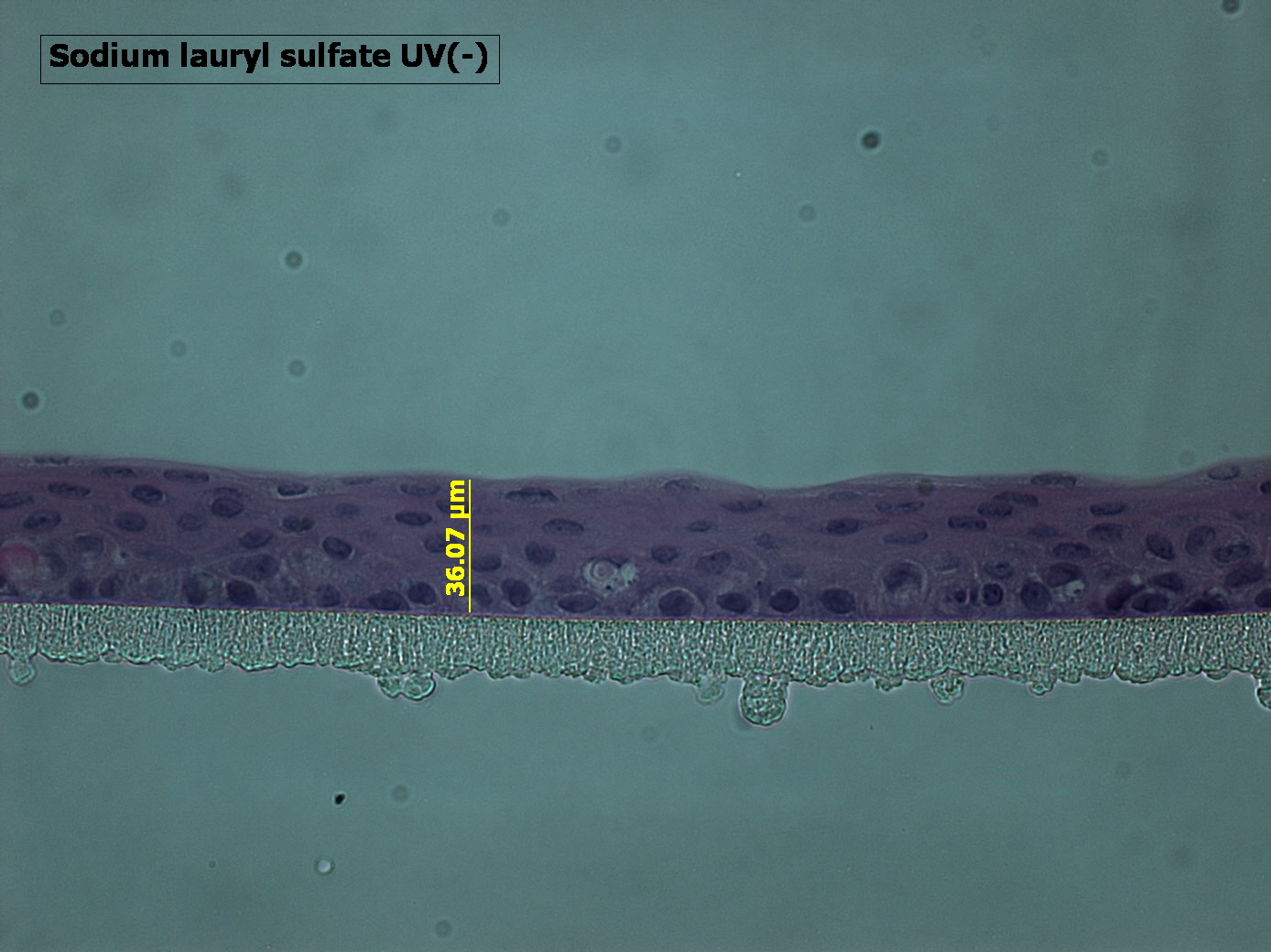

Supplement: S1 Fig — (ZIP) [file pone.0196735.s004.zip › HCM(H&E) staning raw data/HCM/Cornea_S-/SNAP-143147-0042_1.jpg]

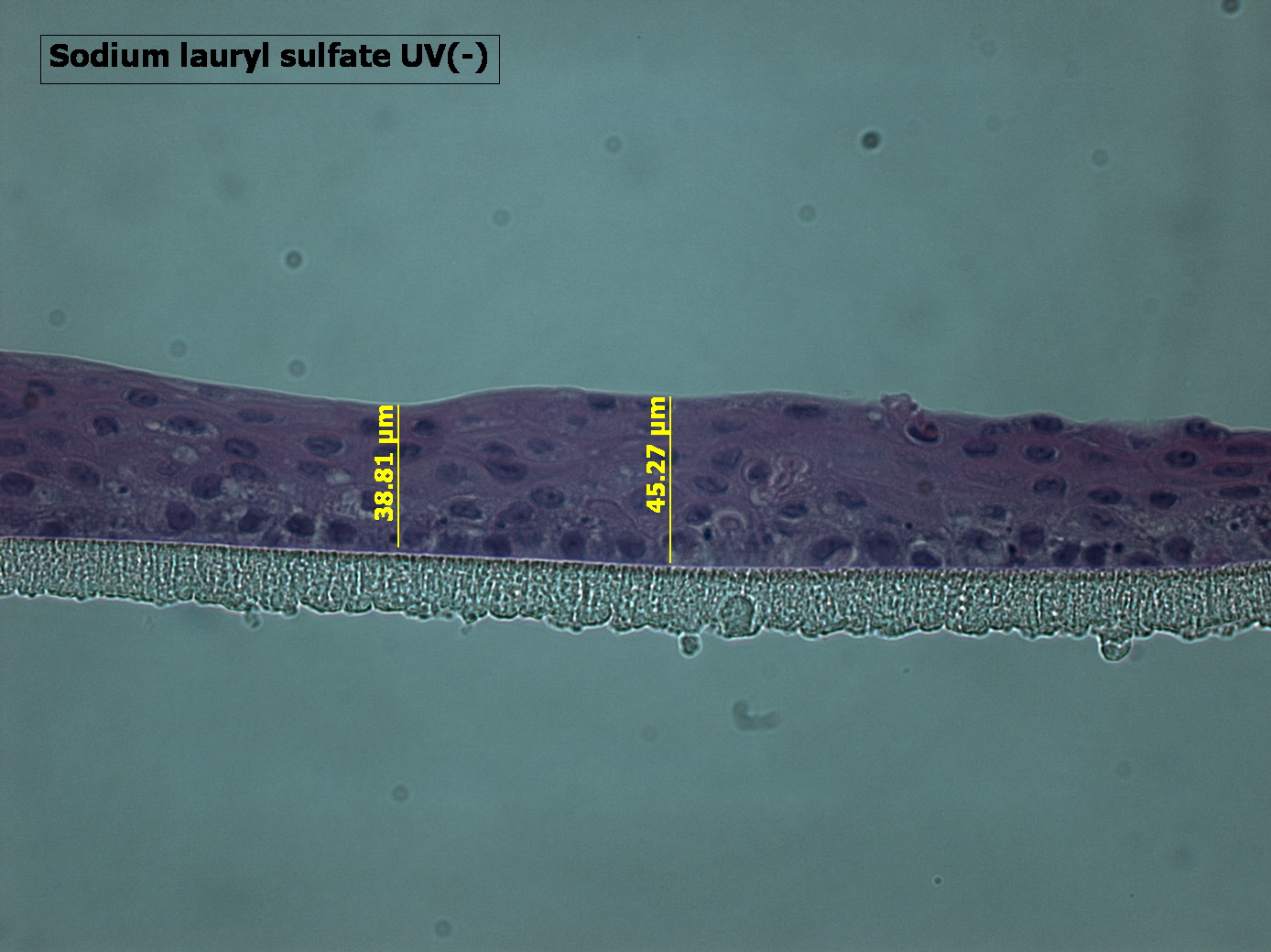

Supplement: S1 Fig — (ZIP) [file pone.0196735.s004.zip › HCM(H&E) staning raw data/HCM/Cornea_S-/SNAP-143328-0046_1.jpg]

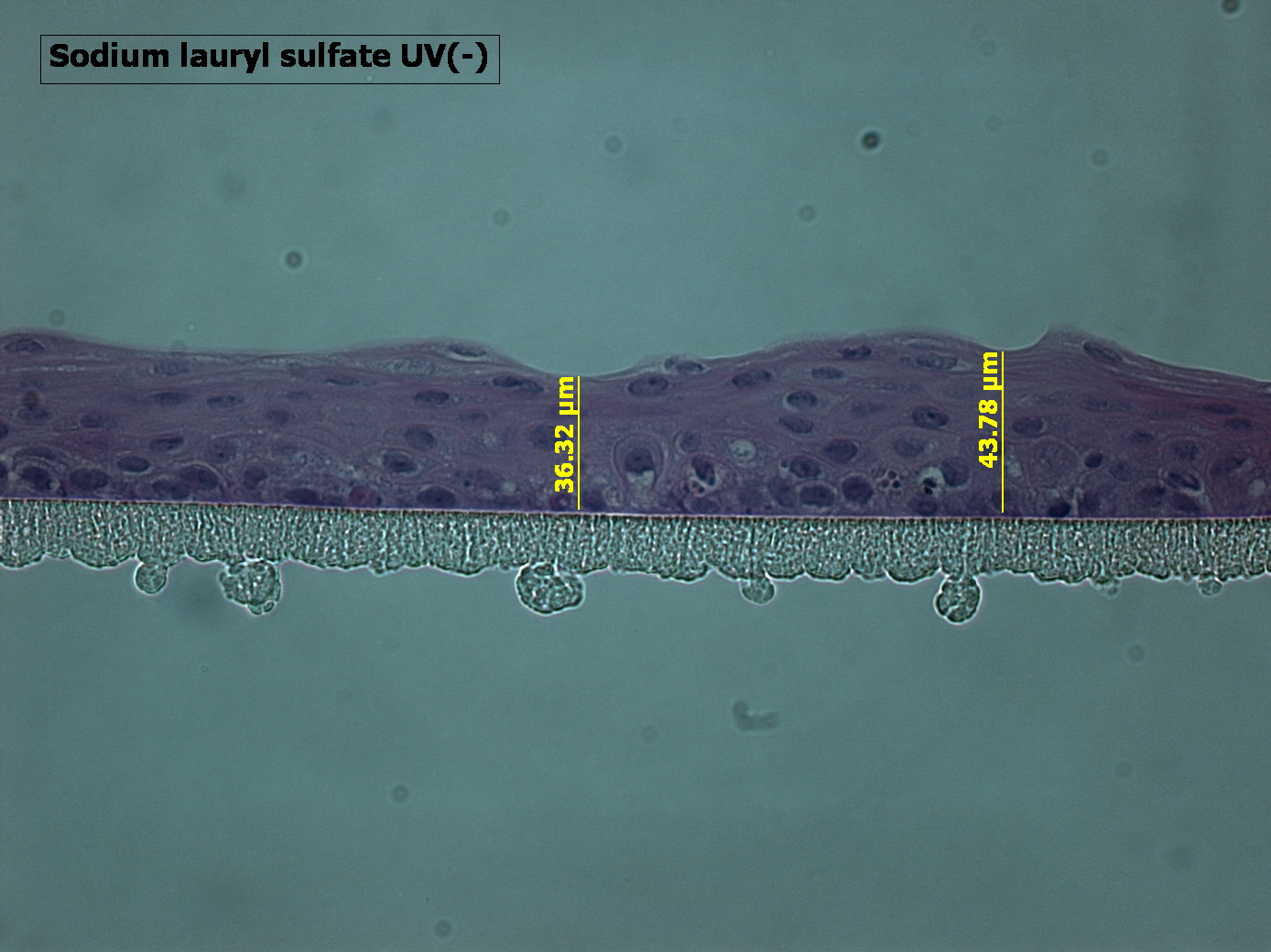

Supplement: S1 Fig — (ZIP) [file pone.0196735.s004.zip › HCM(H&E) staning raw data/HCM/Cornea_S-/SNAP-143352-0048_1.jpg]

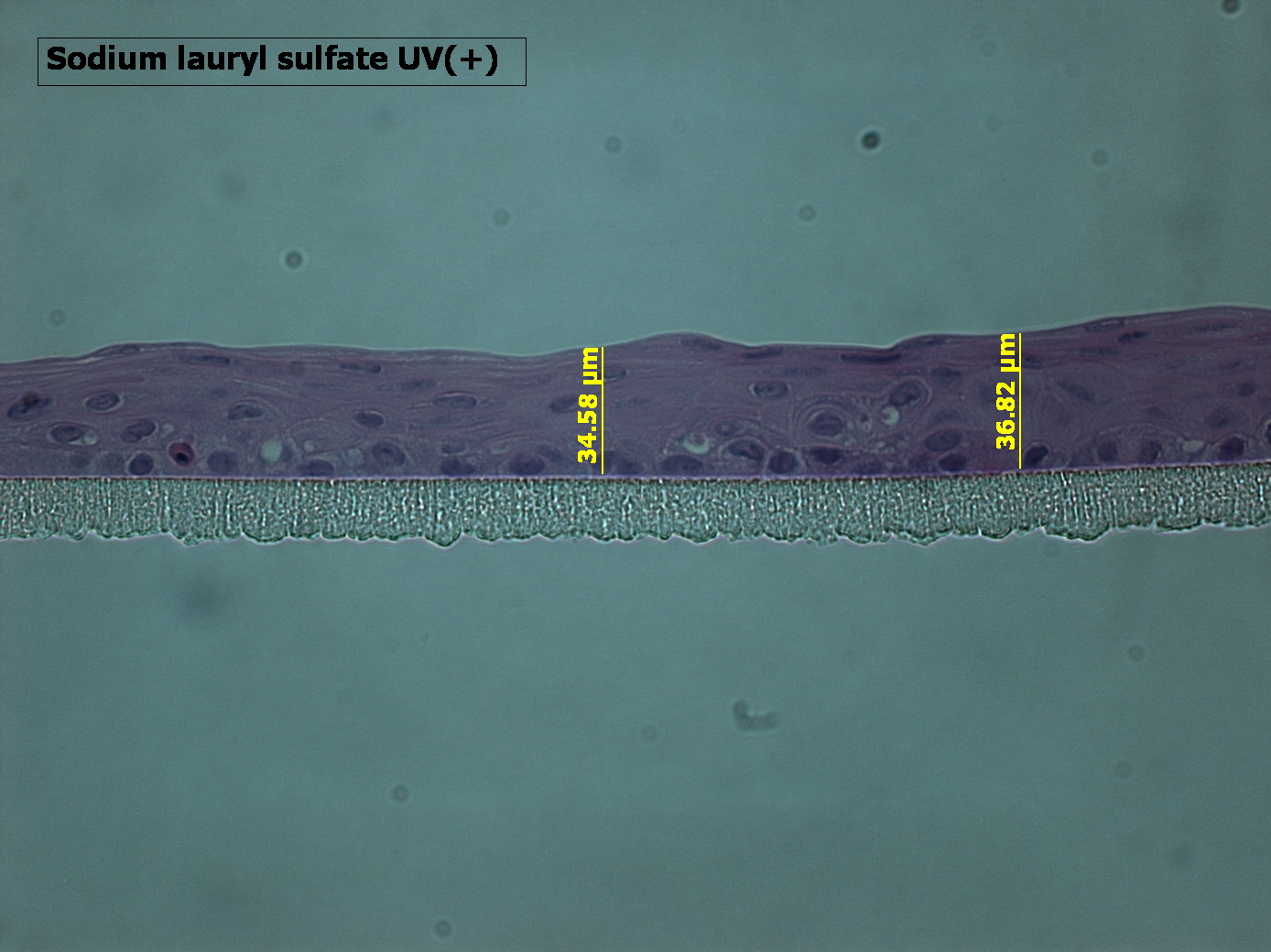

Supplement: S1 Fig — (ZIP) [file pone.0196735.s004.zip › HCM(H&E) staning raw data/HCM/Cornea_S+/SNAP-143726-0050_1.jpg]

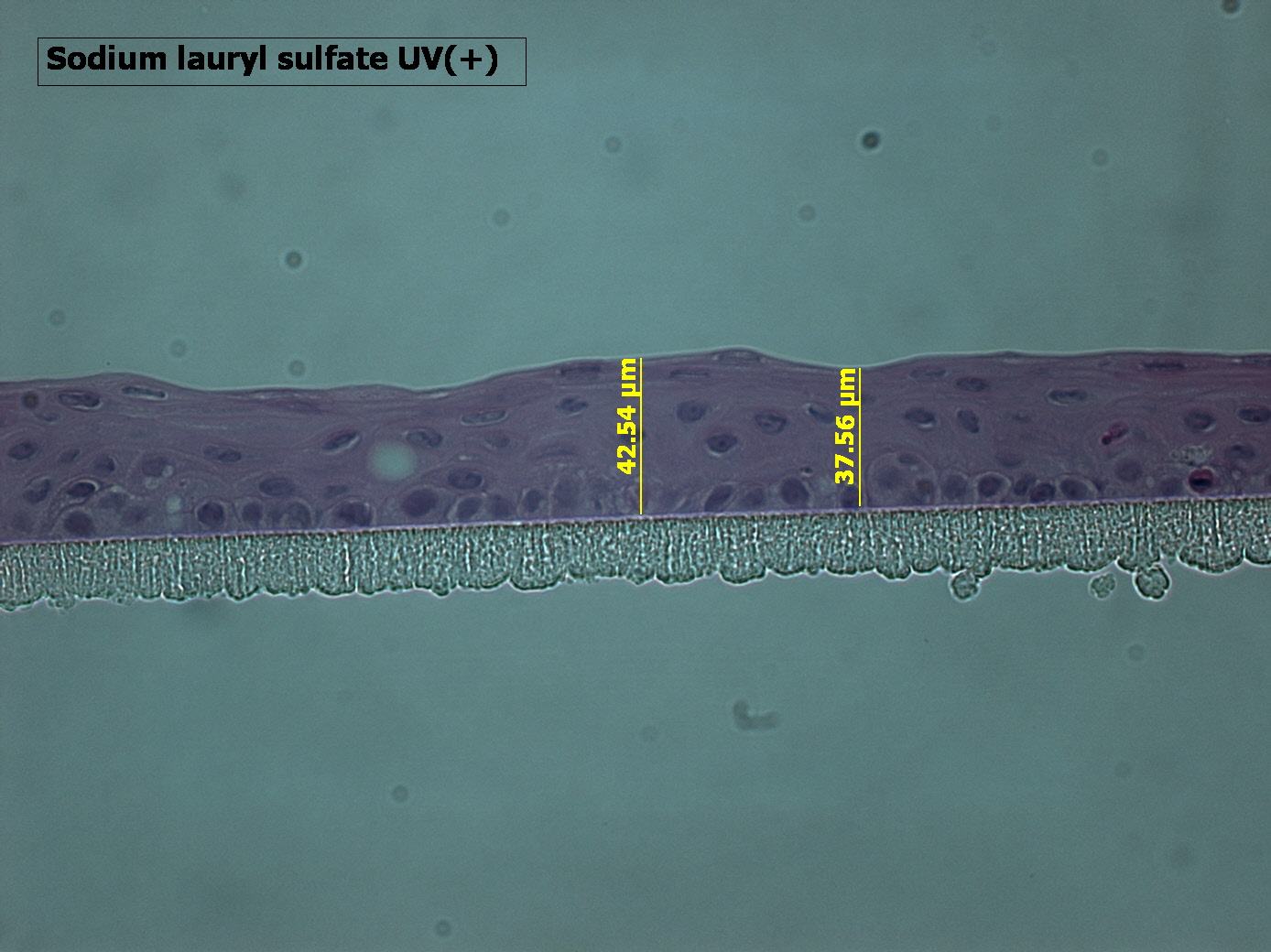

Supplement: S1 Fig — (ZIP) [file pone.0196735.s004.zip › HCM(H&E) staning raw data/HCM/Cornea_S+/SNAP-143742-0051_1.jpg]

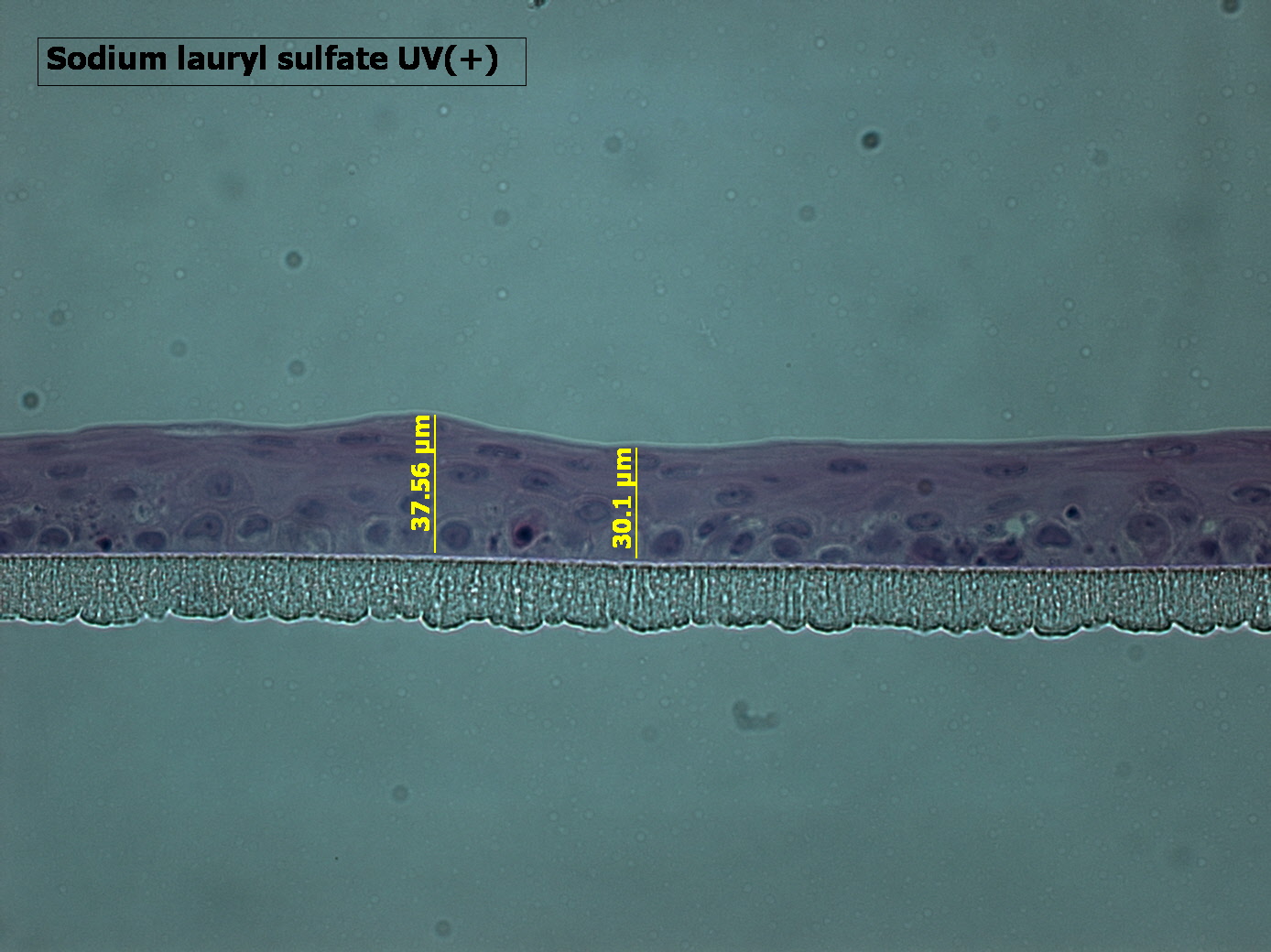

Supplement: S1 Fig — (ZIP) [file pone.0196735.s004.zip › HCM(H&E) staning raw data/HCM/Cornea_S+/SNAP-143802-0053_1.jpg]

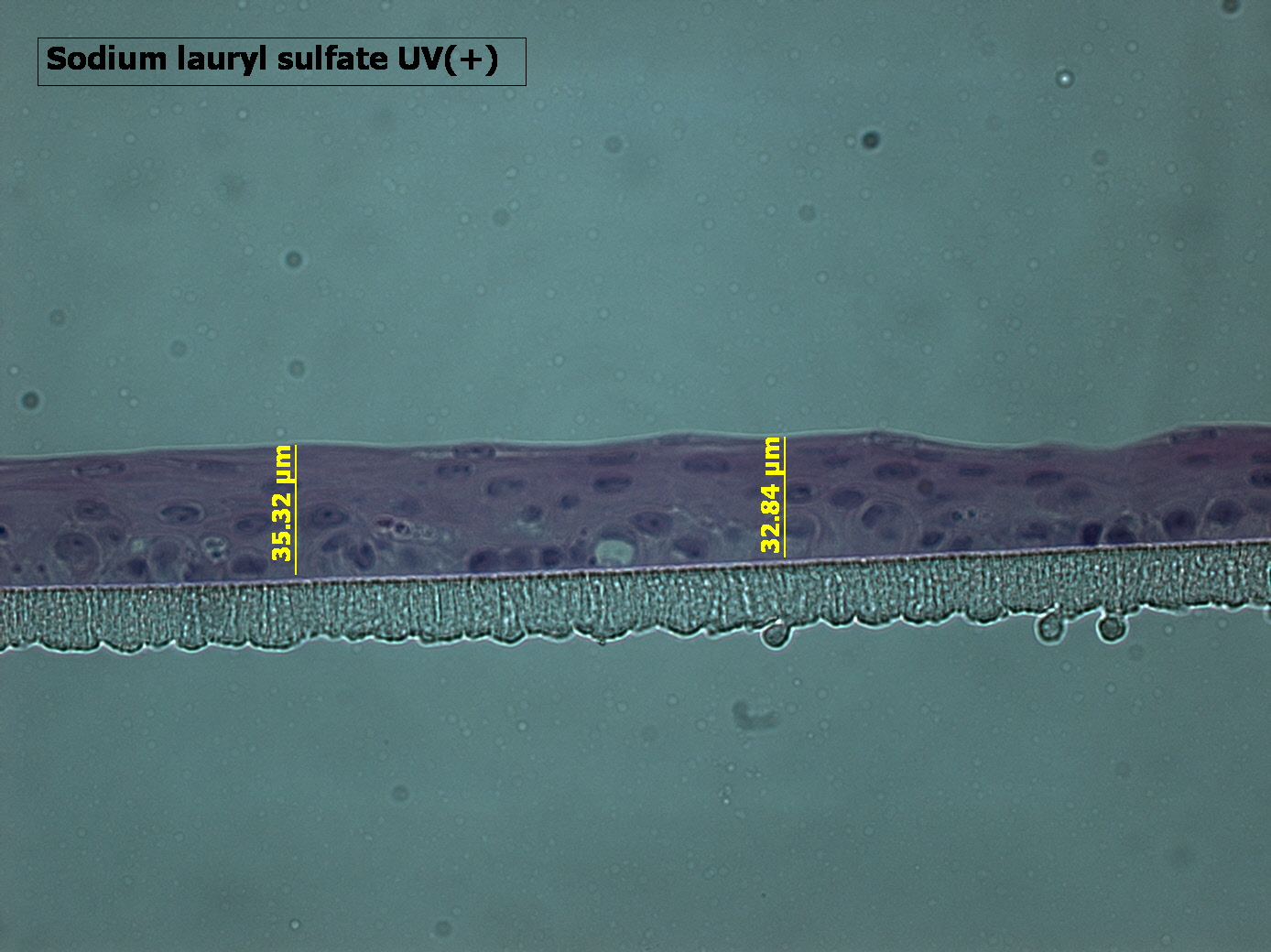

Supplement: S1 Fig — (ZIP) [file pone.0196735.s004.zip › HCM(H&E) staning raw data/HCM/Cornea_S+/SNAP-143808-0054_1.jpg]

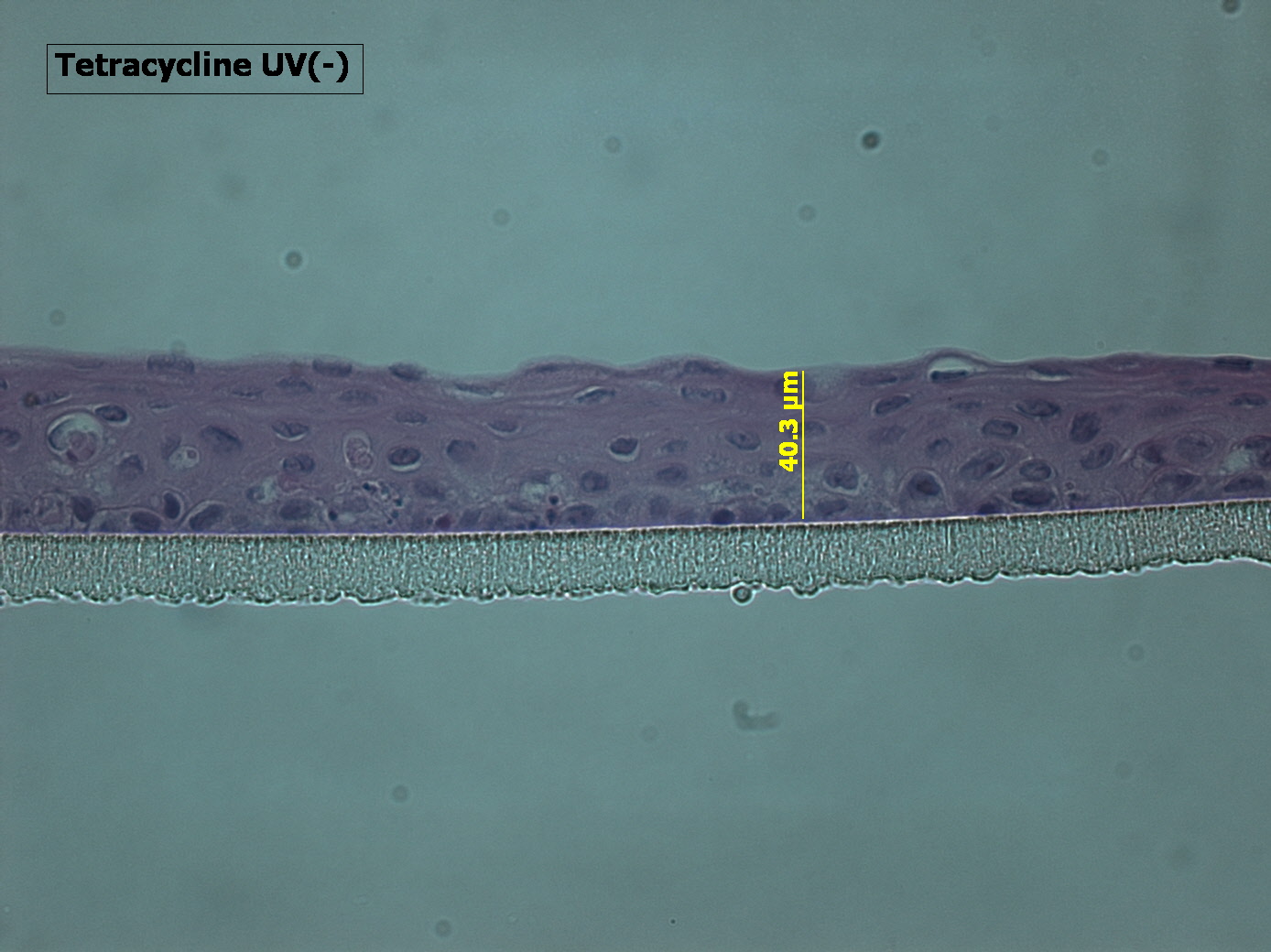

Supplement: S1 Fig — (ZIP) [file pone.0196735.s004.zip › HCM(H&E) staning raw data/HCM/Cornea_T-/SNAP-141617-0017_1.jpg]

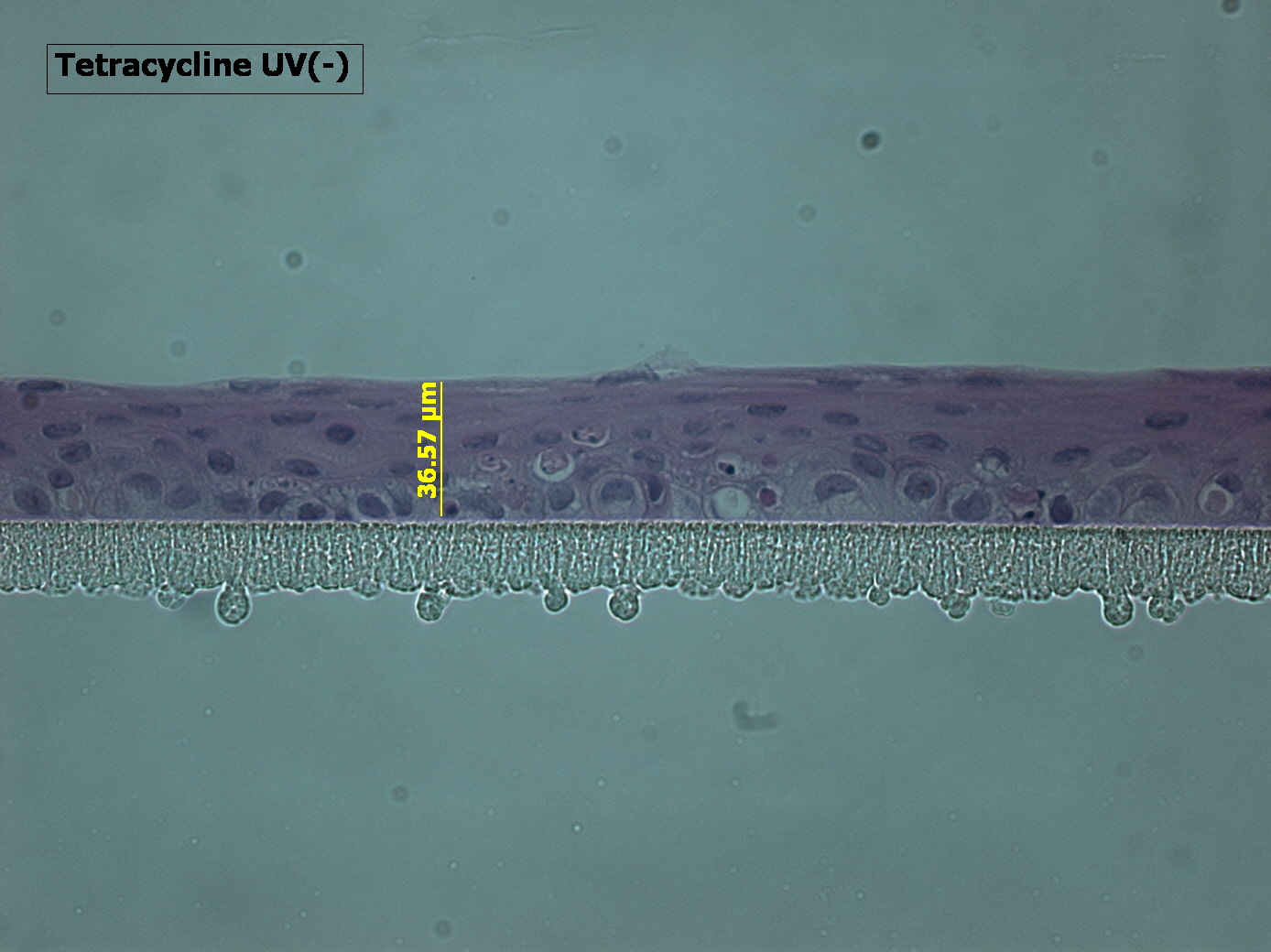

Supplement: S1 Fig — (ZIP) [file pone.0196735.s004.zip › HCM(H&E) staning raw data/HCM/Cornea_T-/SNAP-141722-0020_1.jpg]

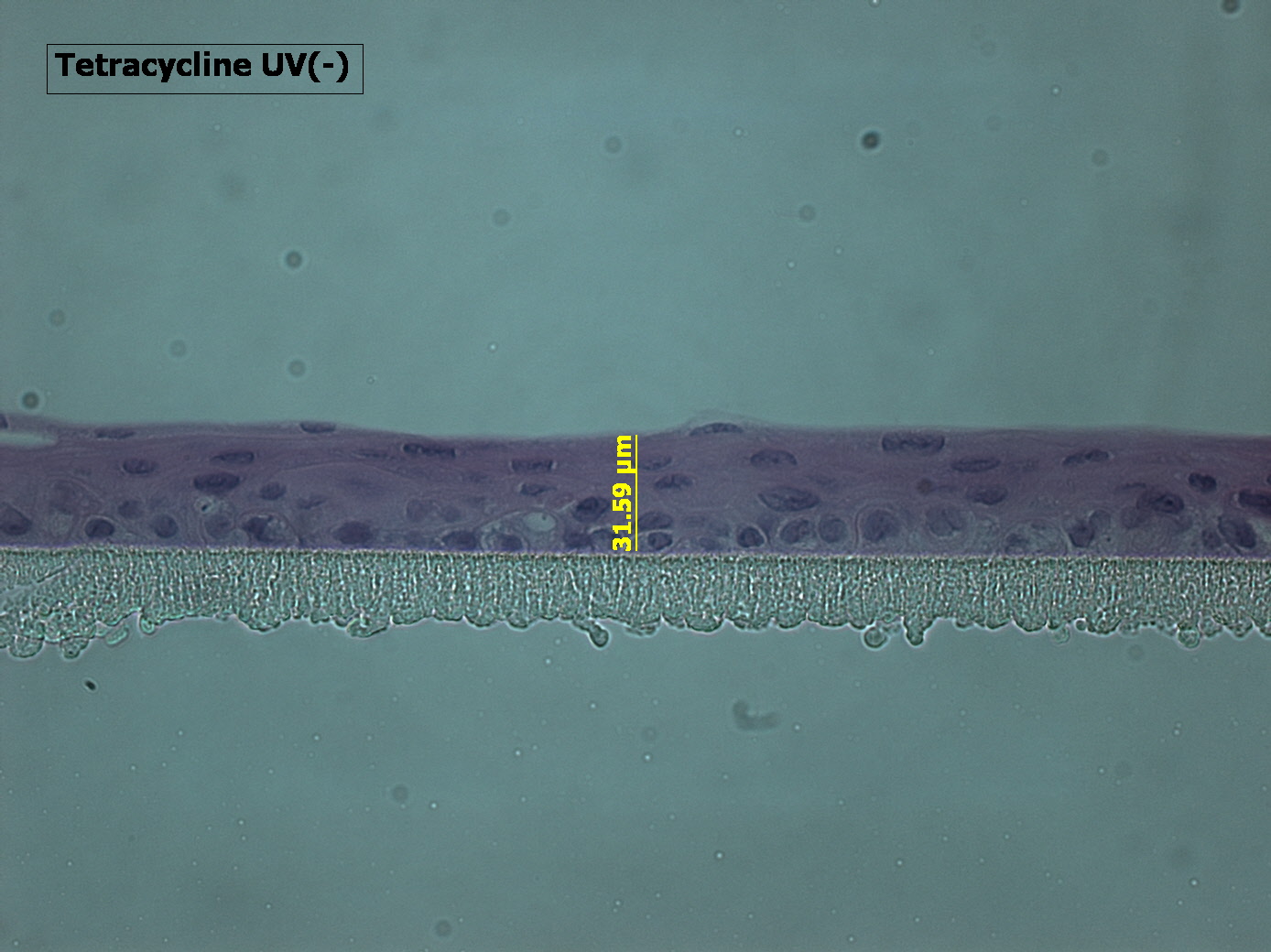

Supplement: S1 Fig — (ZIP) [file pone.0196735.s004.zip › HCM(H&E) staning raw data/HCM/Cornea_T-/SNAP-141740-0021_1.jpg]

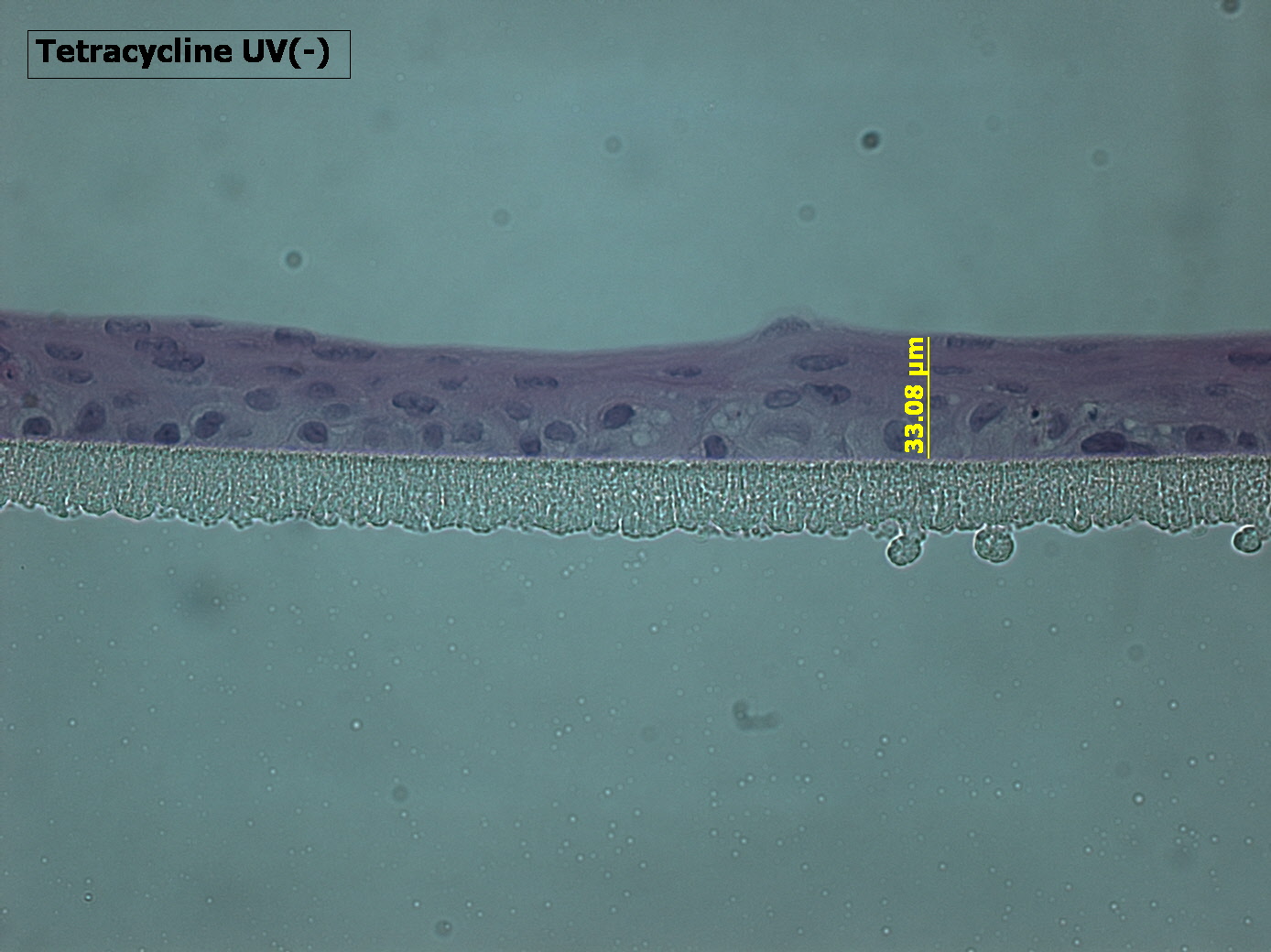

Supplement: S1 Fig — (ZIP) [file pone.0196735.s004.zip › HCM(H&E) staning raw data/HCM/Cornea_T-/SNAP-141750-0022_1.jpg]

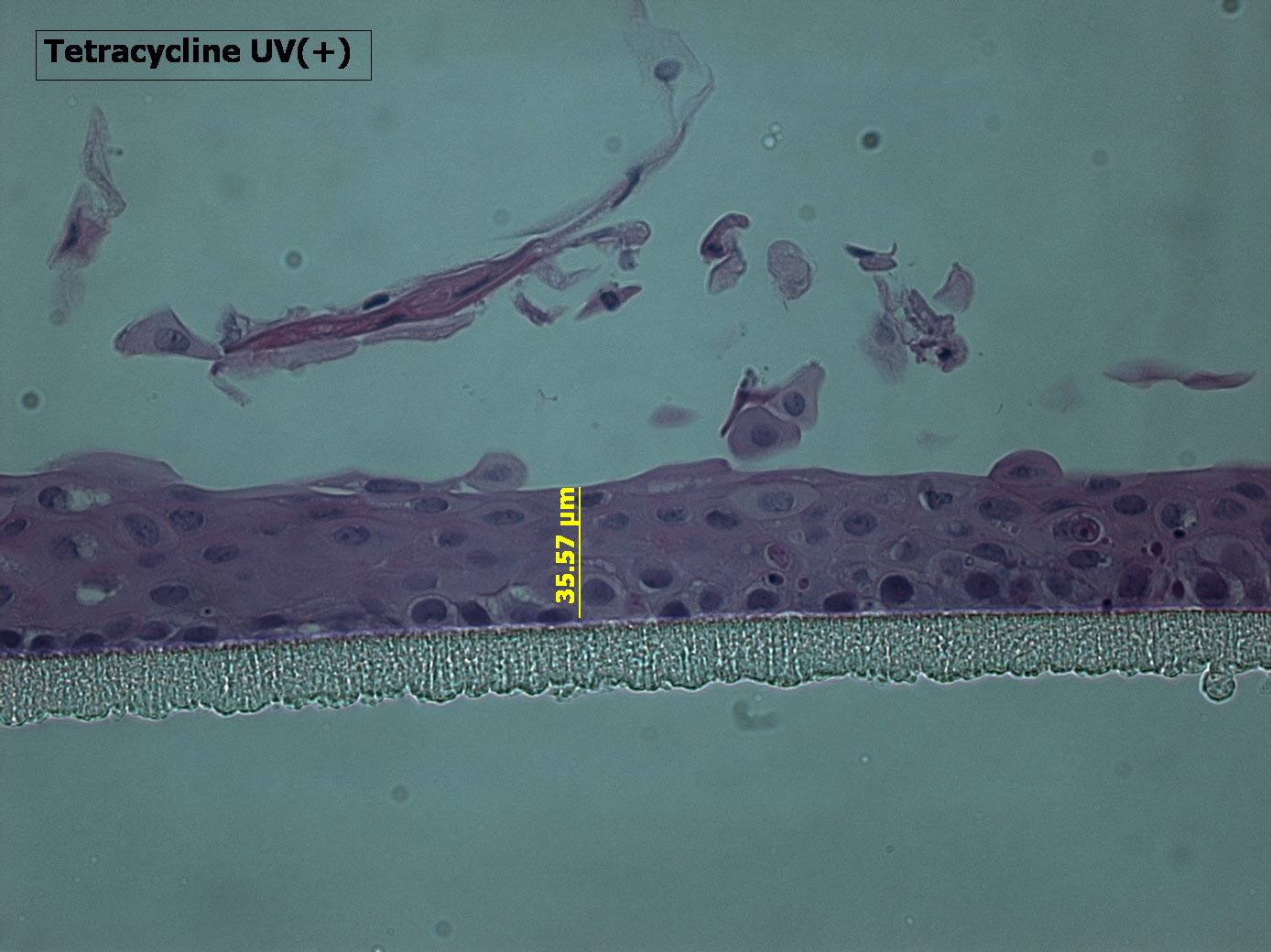

Supplement: S1 Fig — (ZIP) [file pone.0196735.s004.zip › HCM(H&E) staning raw data/HCM/Cornea_T+/SNAP-142055-0026_1.jpg]

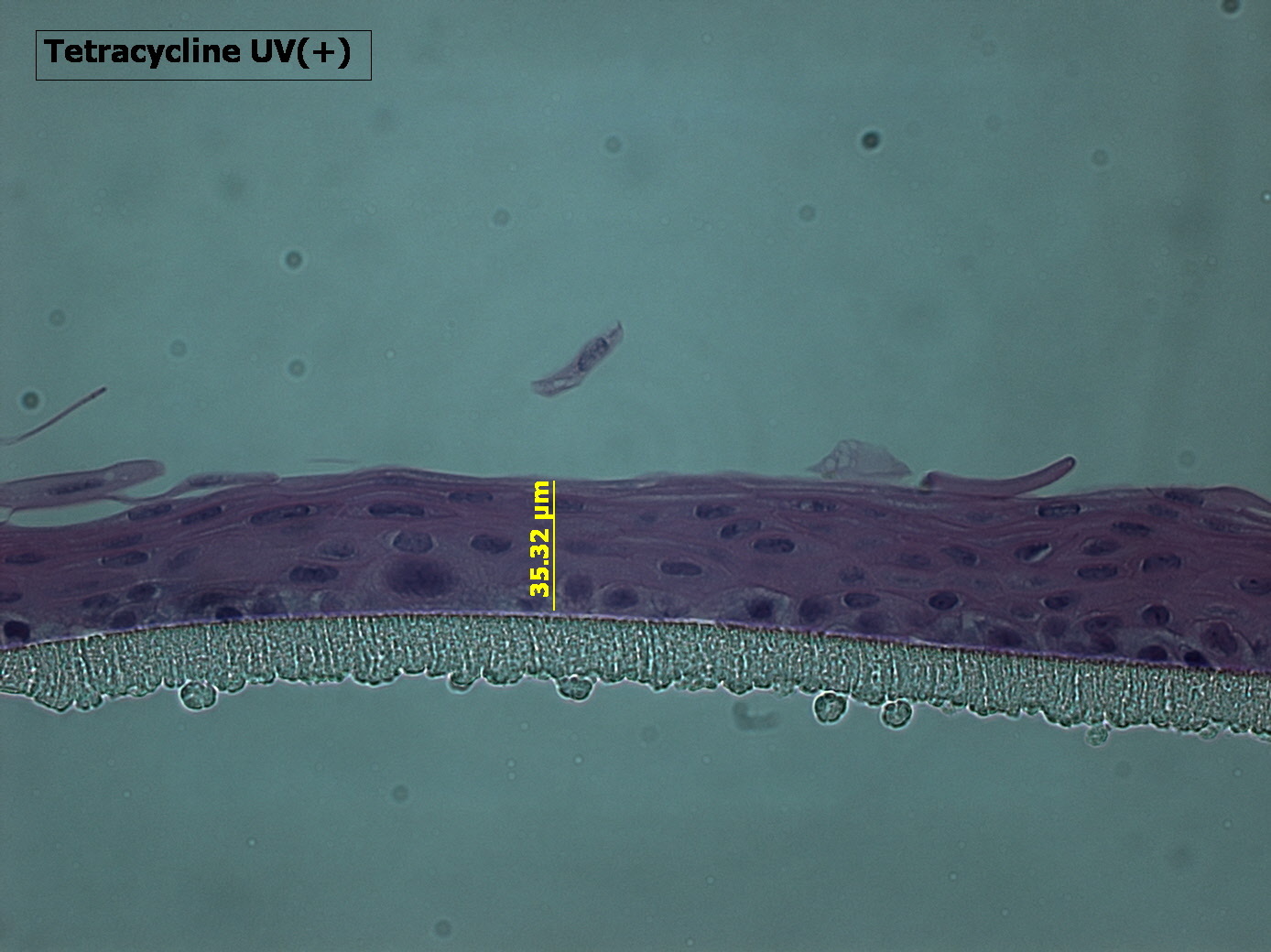

Supplement: S1 Fig — (ZIP) [file pone.0196735.s004.zip › HCM(H&E) staning raw data/HCM/Cornea_T+/SNAP-142111-0028_1.jpg]

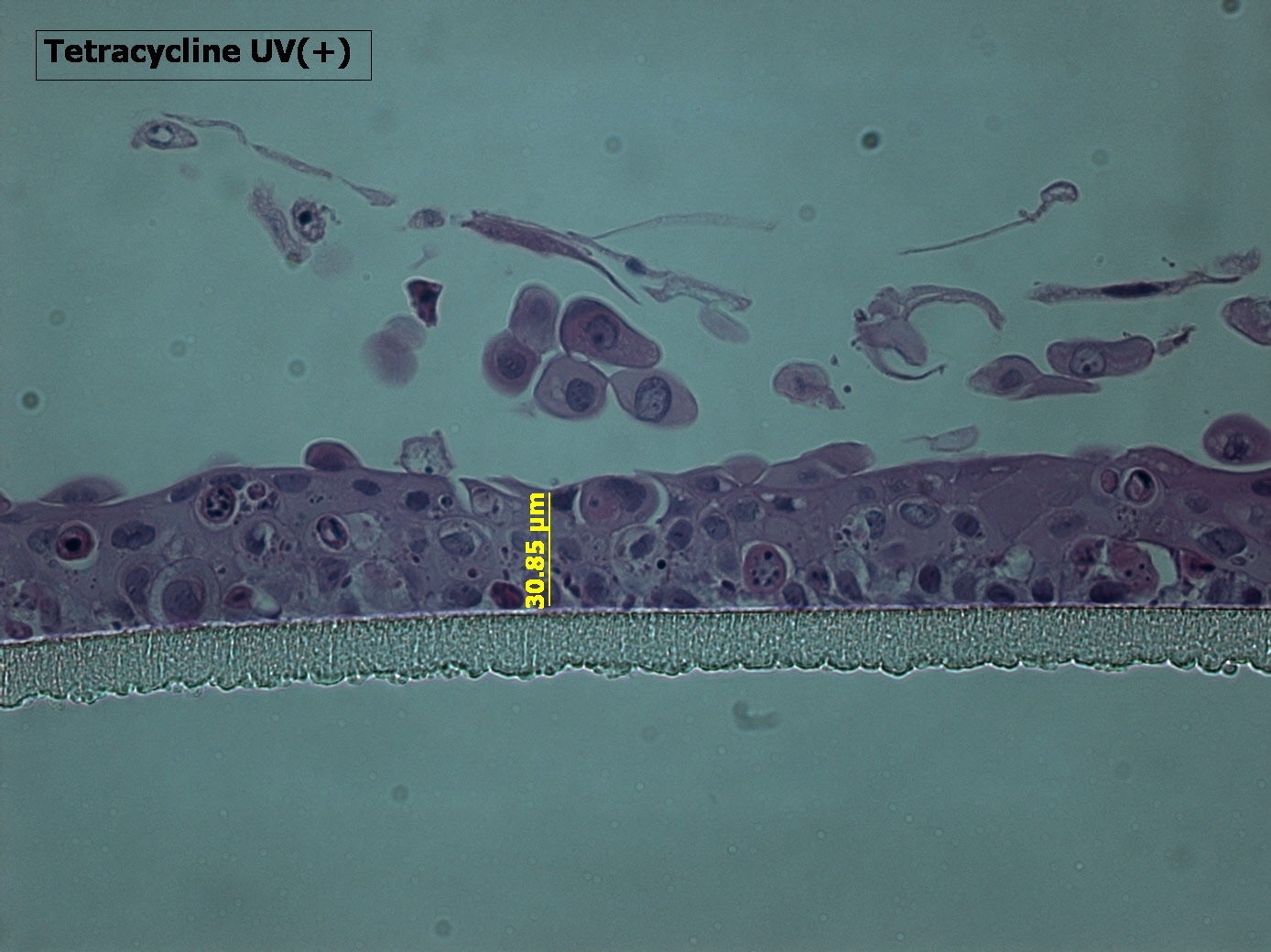

Supplement: S1 Fig — (ZIP) [file pone.0196735.s004.zip › HCM(H&E) staning raw data/HCM/Cornea_T+/SNAP-142200-0031_1.jpg]

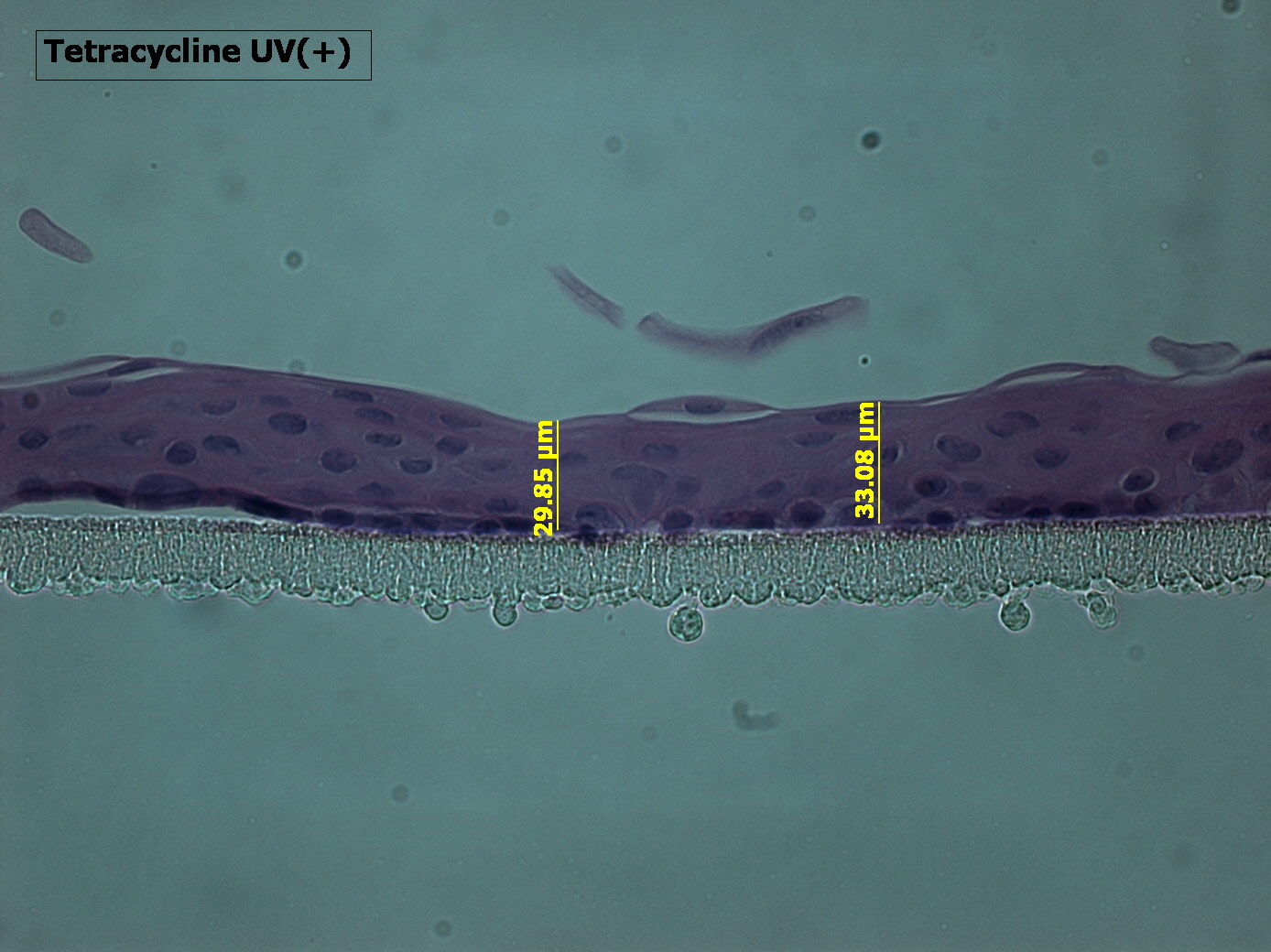

Supplement: S1 Fig — (ZIP) [file pone.0196735.s004.zip › HCM(H&E) staning raw data/HCM/Cornea_T+/SNAP-142525-0035_1.jpg]
